# Evidence of a causal effect of genetic tendency to gain muscle mass on uterine leiomyomata

**DOI:** 10.1038/s41467-023-35974-7

**Published:** 2023-02-01

**Authors:** Eeva Sliz, Jaakko S. Tyrmi, Nilufer Rahmioglu, Krina T. Zondervan, Christian M. Becker, FinnGen, Aarno Palotie, Mark Daly, Bridget Riley-Gills, Howard Jacob, Dirk Paul, Athena Matakidou, Adam Platt, Heiko Runz, Sally John, George Okafo, Nathan Lawless, Heli Salminen-Mankonen, Robert Plenge, Joseph Maranville, Mark McCarthy, Margaret G. Ehm, Kirsi Auro, Simonne Longerich, Caroline Fox, Anders Mälarstig, Katherine Klinger, Clement Chatelain, Matthias Gossel, Karol Estrada, Robert Graham, Robert Yang, Chris O´Donnell, Tomi P. Mäkelä, Jaakko Kaprio, Petri Virolainen, Antti Hakanen, Terhi Kilpi, Markus Perola, Jukka Partanen, Anne Pitkäranta, Taneli Raivio, Raisa Serpi, Tarja Laitinen, Veli-Matti Kosma, Jari Laukkanen, Marco Hautalahti, Outi Tuovila, Raimo Pakkanen, Jeffrey Waring, Bridget Riley-Gillis, Fedik Rahimov, Ioanna Tachmazidou, Chia-Yen Chen, Zhihao Ding, Marc Jung, Shameek Biswas, Rion Pendergrass, David Pulford, Neha Raghavan, Adriana Huertas-Vazquez, Jae-Hoon Sul, Xinli Hu, Åsa Hedman, Manuel Rivas, Dawn Waterworth, Nicole Renaud, Ma´en Obeidat, Samuli Ripatti, Johanna Schleutker, Mikko Arvas, Olli Carpén, Reetta Hinttala, Arto Mannermaa, Katriina Aalto-Setälä, Mika Kähönen, Johanna Mäkelä, Reetta Kälviäinen, Valtteri Julkunen, Hilkka Soininen, Anne Remes, Mikko Hiltunen, Jukka Peltola, Minna Raivio, Pentti Tienari, Juha Rinne, Roosa Kallionpää, Juulia Partanen, Ali Abbasi, Adam Ziemann, Nizar Smaoui, Anne Lehtonen, Susan Eaton, Sanni Lahdenperä, Natalie Bowers, Edmond Teng, Fanli Xu, Laura Addis, John Eicher, Qingqin S. Li, Karen He, Ekaterina Khramtsova, Martti Färkkilä, Jukka Koskela, Sampsa Pikkarainen, Airi Jussila, Katri Kaukinen, Timo Blomster, Mikko Kiviniemi, Markku Voutilainen, Tim Lu, Linda McCarthy, Amy Hart, Meijian Guan, Jason Miller, Kirsi Kalpala, Melissa Miller, Kari Eklund, Antti Palomäki, Pia Isomäki, Laura Pirilä, Oili Kaipiainen-Seppänen, Johanna Huhtakangas, Nina Mars, Apinya Lertratanakul, Marla Hochfeld, Jorge Esparza Gordillo, Fabiana Farias, Nan Bing, Margit Pelkonen, Paula Kauppi, Hannu Kankaanranta, Terttu Harju, Riitta Lahesmaa, Glenda Lassi, Hubert Chen, Joanna Betts, Rajashree Mishra, Majd Mouded, Debby Ngo, Teemu Niiranen, Felix Vaura, Veikko Salomaa, Kaj Metsärinne, Jenni Aittokallio, Jussi Hernesniemi, Daniel Gordin, Juha Sinisalo, Marja-Riitta Taskinen, Tiinamaija Tuomi, Timo Hiltunen, Amanda Elliott, Mary Pat Reeve, Sanni Ruotsalainen, Benjamin Challis, Audrey Chu, Dermot Reilly, Mike Mendelson, Jaakko Parkkinen, Tuomo Meretoja, Heikki Joensuu, Johanna Mattson, Eveliina Salminen, Annika Auranen, Peeter Karihtala, Päivi Auvinen, Klaus Elenius, Esa Pitkänen, Relja Popovic, Jennifer Schutzman, Diptee Kulkarni, Alessandro Porello, Andrey Loboda, Heli Lehtonen, Stefan McDonough, Sauli Vuoti, Kai Kaarniranta, Joni A. Turunen, Terhi Ollila, Hannu Uusitalo, Juha Karjalainen, Mengzhen Liu, Stephanie Loomis, Erich Strauss, Hao Chen, Kaisa Tasanen, Laura Huilaja, Katariina Hannula-Jouppi, Teea Salmi, Sirkku Peltonen, Leena Koulu, David Choy, Ying Wu, Pirkko Pussinen, Aino Salminen, Tuula Salo, David Rice, Pekka Nieminen, Ulla Palotie, Maria Siponen, Liisa Suominen, Päivi Mäntylä, Ulvi Gursoy, Vuokko Anttonen, Kirsi Sipilä, Hannele Laivuori, Venla Kurra, Laura Kotaniemi-Talonen, Oskari Heikinheimo, Ilkka Kalliala, Lauri Aaltonen, Varpu Jokimaa, Marja Vääräsmäki, Laure Morin-Papunen, Maarit Niinimäki, Terhi Piltonen, Katja Kivinen, Elisabeth Widen, Taru Tukiainen, Niko Välimäki, Eija Laakkonen, Heidi Silven, Riikka Arffman, Susanna Savukoski, Triin Laisk, Natalia Pujol, Janet Kumar, Iiris Hovatta, Erkki Isometsä, Hanna Ollila, Jaana Suvisaari, Thomas Damm Als, Antti Mäkitie, Argyro Bizaki-Vallaskangas, Sanna Toppila-Salmi, Tytti Willberg, Elmo Saarentaus, Antti Aarnisalo, Elisa Rahikkala, Kristiina Aittomäki, Fredrik Åberg, Mitja Kurki, Aki Havulinna, Juha Mehtonen, Priit Palta, Shabbeer Hassan, Pietro Della Briotta Parolo, Wei Zhou, Mutaamba Maasha, Susanna Lemmelä, Aoxing Liu, Arto Lehisto, Andrea Ganna, Vincent Llorens, Henrike Heyne, Joel Rämö, Rodos Rodosthenous, Satu Strausz, Tuula Palotie, Kimmo Palin, Javier Garcia-Tabuenca, Harri Siirtola, Tuomo Kiiskinen, Jiwoo Lee, Kristin Tsuo, Kati Kristiansson, Kati Hyvärinen, Jarmo Ritari, Katri Pylkäs, Minna Karjalainen, Tuomo Mantere, Eeva Kangasniemi, Sami Heikkinen, Nina Pitkänen, Samuel Lessard, Clément Chatelain, Perttu Terho, Tiina Wahlfors, Eero Punkka, Sanna Siltanen, Teijo Kuopio, Anu Jalanko, Huei-Yi Shen, Risto Kajanne, Mervi Aavikko, Henna Palin, Malla-Maria Linna, Masahiro Kanai, Zhili Zheng, L. Elisa Lahtela, Mari Kaunisto, Elina Kilpeläinen, Timo P. Sipilä, Oluwaseun Alexander Dada, Awaisa Ghazal, Anastasia Kytölä, Rigbe Weldatsadik, Kati Donner, Anu Loukola, Päivi Laiho, Tuuli Sistonen, Essi Kaiharju, Markku Laukkanen, Elina Järvensivu, Sini Lähteenmäki, Lotta Männikkö, Regis Wong, Auli Toivola, Minna Brunfeldt, Hannele Mattsson, Sami Koskelainen, Tero Hiekkalinna, Teemu Paajanen, Kalle Pärn, Mart Kals, Shuang Luo, Shanmukha Sampath Padmanabhuni, Marianna Niemi, Javier Gracia-Tabuenca, Mika Helminen, Tiina Luukkaala, Iida Vähätalo, Jyrki Tammerluoto, Sarah Smith, Tom Southerington, Petri Lehto, Outi Uimari, Johannes Kettunen

**Affiliations:** 1 Center for Life Course Health Research, Faculty of Medicine, University of Oulu, Oulu, Finland; 2 Biocenter Oulu, Oulu, Finland; 3 Oxford Endometriosis CaRe Centre, Nuffield Department of Women’s and Reproductive Health, University of Oxford, Oxford, UK; 4 Wellcome Centre for Human Genetics, University of Oxford, Oxford, UK; 5 Department of Obstetrics and Gynecology, Oulu University Hospital, Oulu, Finland; 6 PEDEGO Research Unit, University of Oulu and Oulu University Hospital, Oulu, Finland; 7 Medical Research Center Oulu, University of Oulu and Oulu University Hospital, Oulu, Finland; 8 Institute for Molecular Medicine Finland (FIMM), HiLIFE, University of Helsinki, Helsinki, Finland; 9 Broad Institute of MIT and Harvard, Cambridge, MA USA; 10 Massachusetts General Hospital, Boston, MA USA; 11 Abbvie, Chicago, IL US; 12 Astra Zeneca, Cambridge, United Kingdom; 13 Biogen, Cambridge, MA US; 14 Boehringer Ingelheim, Ingelheim am Rhein, Rhein, Germany; 15 Bristol Myers Squibb, New York, NY US; 16 Genentech, San Francisco, CA US; 17 GlaxoSmithKline, Collegeville, PA US; 18 GlaxoSmithKline, Espoo, Finland; 19 Merck, Kenilworth, NJ US; 20 Pfizer, New York, NY US; 21 Translational Sciences, Sanofi R&D, Framingham, MA USA; 22 Maze Therapeutics, San Francisco, CA US; 23 Janssen Biotech, Beerse, Belgium; 24 Novartis Institutes for BioMedical Research, Cambridge, MA US; 25 HiLIFE, University of Helsinki, Finland, Finland; 26 Auria Biobank, University of Turku, Hospital District of Southwest Finland, Turku, Finland; 27 THL Biobank, Finnish Institute for Health and Welfare (THL), Helsinki, Finland; 28 Finnish Red Cross Blood Service, Finnish Hematology Registry and Clinical Biobank, Helsinki, Finland; 29 Helsinki Biobank, Helsinki University and Hospital District of Helsinki and Uusimaa, Helsinki, Finland; 30 Northern Finland Biobank Borealis, University of Oulu, Northern Ostrobothnia Hospital District, Oulu, Finland; 31 Finnish Clinical Biobank Tampere, University of Tampere, Pirkanmaa Hospital District, Tampere, Finland; 32 Biobank of Eastern Finland, University of Eastern Finland, Northern Savo Hospital District, Kuopio, Finland; 33 Central Finland Biobank, University of Jyväskylä, Central Finland Health Care District, Jyväskylä, Finland; 34 FINBB - Finnish biobank cooperative, Turku, Finland; 35 Business Finland, Helsinki, Finland; 36 GlaxoSmithKline, Stevenage, United Kingdom; 37 Janssen Research & Development, LLC, Spring House, PA US; 38 Faculty of Medicine and Health Technology, Tampere University, Tampere, Finland; 39 Northern Savo Hospital District, Kuopio, Finland; 40 Northern Ostrobothnia Hospital District, Oulu, Finland; 41 University of Eastern Finland, Kuopio, Finland; 42 Pirkanmaa Hospital District, Tampere, Finland; 43 Hospital District of Helsinki and Uusimaa, Helsinki, Finland; 44 Hospital District of Southwest Finland, Turku, Finland; 45 Institute for Molecular Medicine Finland, HiLIFE, University of Helsinki, Helsinki, Finland; 46 GlaxoSmithKline, Brentford, United Kingdom; 47 Janssen Research & Development, LLC, Titusville, NJ 08560 US; 48 University of Gothenburg, Gothenburg, Sweden; 49 Seinäjoki Central Hospital, Seinäjoki, Finland; 50 Tampere University, Tampere, Finland; 51 Novartis, Basel, Switzerland; 52 Finnish Institute for Health and Welfare (THL), Helsinki, Finland; 53 Broad Institute, Cambridge, MA US; 54 Janssen Research & Development, LLC, Boston, MA US; 55 Novartis, Boston, MA US; 56 Janssen-Cilag Oy, Espoo, Finland; 57 Helsinki University Hospital and University of Helsinki, Helsinki, Finland; 58 Eye Genetics Group, Folkhälsan Research Center, Helsinki, Finland; 59 Research Unit of Oral Health Sciences Faculty of Medicine, University of Oulu, Oulu, Finland; 60 Medical Research Center, Oulu, Oulu University Hospital and University of Oulu, Oulu, Finland; 61 University of Helsinki, Helsinki, Finland; 62 University of Jyväskylä, Jyväskylä, Finland; 63 University of Oulu, Oulu, Finland; 64 Estonian biobank, Tartu, Estonia; 65 Aarhus University, Aarhus, Denmark; 66 Department of Otorhinolaryngology – Head and Neck Surgery, University of Helsinki and Helsinki University Hospital, Helsinki, Finland; 67 Department of Medical Genetics, Helsinki University Central Hospital, Helsinki, Finland; 68 Transplantation and Liver Surgery Clinic, Helsinki University Hospital, Helsinki University, Helsinki, Finland; 69 University of Helsinki and Hospital District of Helsinki and Uusimaa, Helsinki, Finland; 70 University of Tampere, Tampere, Finland; 71 Finnish Red Cross Blood Service, Helsinki, Finland

**Keywords:** Reproductive disorders, Genome-wide association studies, Cancer genetics, Gynaecological cancer

## Abstract

Uterine leiomyomata (UL) are the most common tumours of the female genital tract and the primary cause of surgical removal of the uterus. Genetic factors contribute to UL susceptibility. To add understanding to the heritable genetic risk factors, we conduct a genome-wide association study (GWAS) of UL in up to 426,558 European women from FinnGen and a previous UL meta-GWAS. In addition to the 50 known UL loci, we identify 22 loci that have not been associated with UL in prior studies. UL-associated loci harbour genes enriched for development, growth, and cellular senescence. Of particular interest are the smooth muscle cell differentiation and proliferation-regulating genes functioning on the myocardin-cyclin dependent kinase inhibitor 1 A pathway. Our results further suggest that genetic predisposition to increased fat-free mass may be causally related to higher UL risk, underscoring the involvement of altered muscle tissue biology in UL pathophysiology. Overall, our findings add to the understanding of the genetic pathways underlying UL, which may aid in developing novel therapeutics.

## Abstract (web summary)

Many genetic factors that contribute to uterine leiomyomata (UL) - the most common tumours of the female genital tract - remain to be discovered. Here, the authors conduct a UL meta-genome-wide association study, and find loci related to altered muscle tissue biology that are associated with UL.

## Introduction

Uterine leiomyomata (UL) are the most common benign tumours of the female genital tract, with an estimated lifetime incidence of up to 70%^1^ and the primary cause of hysterectomy. Female sex hormones stimulate UL growth and, thus, UL are almost exclusively found in females of reproductive age. UL are present in single or multiple numbers, with sizes ranging from millimetres to 20 cm or more in diameter^2^, and they are composed mostly of smooth muscle cells (SMC) and fibroblasts with a profound component of extracellular matrix (ECM). In 25–50% of women with ULs, the enlarged and deformed uterus causes symptoms that reduce the quality of life, such as heavy or prolonged menstrual bleeding resulting in anaemia, reduced fertility and pregnancy complications^3^.

Until recently, the focus in the genetics of UL has been on somatic rearrangements, and key driver variations, for example, in *MED12* and *HMGA2* have been reported^4^. Familial aggregation, the disparity in prevalence between different ethnic groups, and high heritability estimates obtained in twin studies (h^2^ up to 69%) suggest, however, that heritable genetic factors modulate UL risk^5–8^. To date, 11 GWASs on UL have been conducted in populations of European, Japanese, and African ancestries^9–19^. In a recent UL meta-GWAS, the SNP-based heritability of UL was estimated to be 2.8%^12^, suggesting that there may be other genetic variants contributing to UL susceptibility that are yet to be discovered.

Significant GWAS findings provide opportunities for testing causal inferences between UL and traits associated with UL. For instance, the causal relationship between UL and excessive menstrual bleeding has been demonstrated using the Mendelian randomisation method^12^. The causal inferences between UL and metabolic risk factors, such as blood lipid levels or body mass index (BMI), however, have not been extensively studied even if previous cross-sectional studies indicate that those are associated with UL risk^20^.

In this work, we conducted two sets of meta-analyses with data from FinnGen and a previously published UL meta-GWAS^12^ in order to add understanding to the UL-related heritable genetic risk factors. We further utilised the GWAS results to estimate genetic correlations and causal relationships between UL and metabolic and anthropometric traits. Our findings provide a different perspective on UL pathobiology and suggest an involvement of fat-free mass rather than fat mass in the underlying causal pathway.

## Results

### 22 uterine leiomyomata-associated loci that have not been described in prior studies

**Fig. 1:**
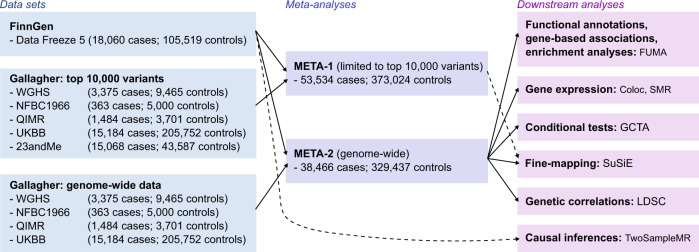
Study setting. The flow diagram illustrates the data usage and analytical steps of our study. We conducted a GWAS of uterine leiomyomata (UL) in 18,060 cases and 150,519 female controls from the FinnGen project. Subsequently, we meta-analysed the FinnGen-based results with summary statistics from a previous UL meta-GWAS^12^. META-1 included 53,534 cases and 373,024 female controls and was restricted to the top 10,000 variants from the previous study^12^. META-2 was conducted genome-widely in 38,466 cases and 329,437 female controls, excluding 23andMe data due to the data usage policy. Downstream analyses assessing functional annotations, gene-based associations, pathway enrichment, conditional association tests, fine-mapping, and genetic correlations were conducted using genome-wide summary statistics from META-2. In addition, fine-mapping was also conducted for the results of META-1. In Mendelian randomisation analyses evaluating causal inferences between UL and other, mostly UKBB-based traits, we extracted instruments for UL from the FinnGen-based summary statistics to avoid possible bias from overlapping UKBB samples.

‘META-1’ comprised data from FinnGen and the previous UL meta-GWAS^12^ with up to 53,534 cases and 373,024 female controls, and the analysis was restricted to publicly available 10,000 variants from the previous study^12^. ‘META-2’ was conducted with data from up to 38,466 cases and 329,437 controls from FinnGen and the genome-wide summary statistics from the same study by ref. ^12^. excluding 23andMe data due to the data usage policy. The study setting is illustrated in Fig. 1.

**Fig. 2:**
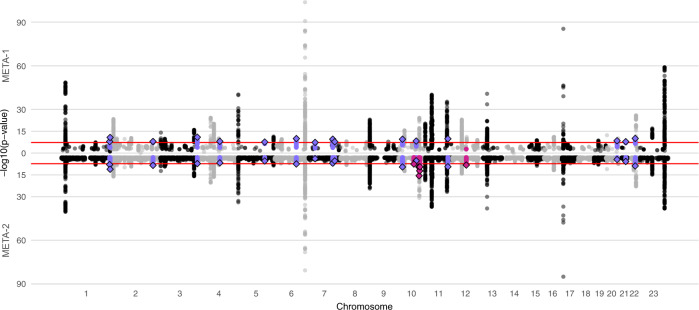
A combined Manhattan plot of uterine leiomyomata (UL) associations in two sets of meta-analyses. We conducted UL GWAS in FinnGen and, subsequently, two sets of meta-analyses with data from a previously published UL meta-analysis:^12^ META-1 (top) was limited to the top 10,000 most significant variants from the previous study^12^, and included up to 53,534 UL cases and 373,024 female controls whereas META-2 (bottom) was conducted genome-widely in 38,466 UL cases and 329,437 female controls. The purple colour denotes UL risk loci identified in META-1 that have not been described in prior studies, and the pink color indicates loci identified in META-2 that were not associated previously with UL risk in prior GWASs or META-1. Black and grey colours indicate odd and even chromosome numbers, respectively. The red dashed lines correspond to the threshold for genome-wide significance (*p* < 5 × 10^−8^).

In META-1, we identified 63 genomic regions located more than 1 Mb apart with at least one variant associating with UL at *p* < 5 × 10^−8^ (Fig. 2, Table S1, and Supplementary Data 1,  2); of these, 16 had not been reported in association with UL in prior UL GWASs (Table 1) while the remaining 47 were in the proximity of known UL risk loci. In META-2, we identified 61 genomic regions, out of which six had not been associated with UL risk in prior GWASs or in META-1 (Fig. 2, Table 1, Table S2, and Supplementary Data 3). However, the association at 10q24.32-10q25.1 likely spans a region larger than the ±1 Mb locus definition overlapping with a previously reported UL association near STN1 subunit of CST complex (*STN1*) and STE20-like kinase (*SLK*)^12^. This expanded association signal appears to be driven by variants enriched in the Finnish population (Finnish enrichment 46x-198x calculated as a ratio of the Finnish allele frequency and the non-Finnish-non-Estonian European allele frequency; Table 1). Regional association plots of the loci that have not been associated with UL risk in prior studies are presented in Figs. S1–S18, and the regional plot of the large signal on chr10 is presented in Fig. S19. Genomic inflation factor of 1.105 suggested minor inflation in the test statistics that was most notably accounted for by a polygenic signal, with the intercept being close to one^21^ (1.0066; Fig. S20). There was very little or no heterogeneity between the results obtained in FinnGen and the previous study^12^ (Table 1 and Fig. S21). We estimated LD score (LDSC) regression-derived SNP-based heritability to be 0.105 (standard error [SE] = 0.011) on the liability scale, which corresponds to an ~7.7 percentage point increase compared with the LDSC-based estimate obtained in the previous study^12^. The SNP-based heritability estimate obtained additionally using SumHer^22^ was 0.034 (SE = 0.003).

**Table 1:** Overview of 22 loci that have not been reported in association with UL in prior studies

Locus	Chr:Pos (hg38)	Nearest gene(s)	Candidate gene(s)	rsID	EA	EAF	OR (95% CI)	*P* value	HetPVal	INFO_FinnGen_	FIN enr.
*META-1*											
1q43	1:241860596	*EXO1*	*EXO1, FH*	rs4149909	G	0.03	1.13 (1.08-1.18)	1.16E-08	0.265	0.996	1.03
1q44	1:244151650	*ZBTB18, C1orf100*	*ZBTB18, AKT3*	rs2183478	G	0.18	1.07 (1.05-1.09)	1.75E-11	0.774	0.969	1.57
2q33.3	2:207258660	*MYOSLID, KLF7*	*MYOSLID*	rs10804157	C	0.44	1.04 (1.03-1.05)	1.04E-08	0.143	0.994	0.87
3q27.2	3:185807411	*IGF2BP2*	*IGF2BP2*	rs13060777	G	0.26	1.05 (1.04-1.07)	1.14E-11	0.595	0.999	1.10
4q23	4:99031559	*METAP1*	*EIF4E, ADH5*	rs1037475	G	0.57	1.04 (1.03-1.05)	8.38E-09	0.707	1.000	0.95
5q31.1	5:133099880	*HSPA4*	*HSPA4*	rs4367292	T	0.27	0.96 (0.94-0.97)	2.49E-08	0.882	0.997	1.15
6q21	6:109054915	*SESN1*	*SESN1*	rs11153158	C	0.13	0.93 (0.92–0.95)	1.05E-10	0.243	0.996	1.19
7p14.3	7:33008785	*FKBP9, NT5C3A*	*NT5C3A, BBS9*	rs4723230	T	0.80	1.05 (1.03-1.07)	4.68E-08	0.946	0.998	1.03
7q31.31	7:121132432	*CPED1*	*WNT16*	rs12706314	A	0.53	1.04 (1.03-1.06)	2.69E-10	0.777	0.998	0.89
7q32.3	7:130935964	*LINC-PINT*	*LINC-PINT*	rs35908158	C	0.08	1.08 (1.05-1.10)	1.60E-08	0.883	0.998	1.41
10p12.31	10:21517903	*SKIDA1*	*DNAJC1*	rs946711	C	0.33	1.05 (1.03-1.06)	2.96E-10	0.460	0.995	0.96
10q23.31	10:88331783	*RNLS*	*RNLS*	rs1426619	T	0.45	1.04 (1.03-1.06)	4.92E-09	0.653	0.998	1.09
11q23.2	11:112703765	*ENSG00000285769*	*ENSG00000285769*	rs10891420	C	0.42	1.05 (1.03-1.06)	1.38E-10	0.019	0.995	1.16
20q13.31	20:57441016	*CTCFL*	*RBM38, BMP7*	rs13039273	C	0.46	1.04 (1.03-1.06)	3.08E-09	0.874	0.995	1.22
21q22.12	21:35072824	*RUNX1*	*RUNX1*	rs2834747	G	0.30	0.96 (0.94-0.97)	1.44E-08	0.711	0.997	1.17
22q12.3	22:36287509	*MYH9, APOL1*	*MYH9*	rs9610482	T	0.19	1.06 (1.04-1.08)	7.89E-11	0.354	0.994	0.94
*META-2*											
10q22.3	10:76884502	*KCNMA1*	*KCNMA1*	rs2082415	T	0.52^f^	1.05 (1.03-1.06)	2.80E-08	0.460	0.949	1.11
10q24.32^a^	10:101726828	*FGF8*	*SLK*	rs189195982	T	0.03^f^	1.26 (1.17-1.36)	2.78E-10	0.285	0.994	198.19
10q24.32^a^	10:102788270	*WBP1L*	*SLK*	rs75731980	T	0.06^f^	1.23 (1.17-1.30)	2.42E-16	0.801	0.992	46.07
10q25.1^a^	10:105587387	*SORCS3*	*SLK*	rs17119191	T	0.97^f^	0.76 (0.70-0.82)	5.81E-13	0.314	0.977	49.89
10q25.1^a^	10:106822067	*SORCS1*	*SLK*	rs1336619	T	0.03^f^	1.25 (1.17-1.34)	3.48E-10	0.127	0.999	7.47
12q15	12:68692314	*NUP107*	*MDM2*	rs142808358	T	0.03^f^	0.87 (0.83-0.91)	5.45E-09	0.958	0.987	0.87

‘Nearest gene(s)’ reports the gene closest to the association lead variant.

‘Candidate gene(s)’ indicates the biologically most relevant gene within a 1  Mb window around the association lead variant.

*Chr* chromosome, *Pos* position (build 38), *EA* effect allele, *EAF* effect allele frequency, *OR* odds ratio, *CI* confidence interval, *P* *p* value, *HetPVal* *p* value for heterogeneity, *INFO*_FinnGen_ imputation info in FinnGen, *FIN enr* Finnish enrichment (calculated as FIN AF/NFEE AF in the Genome Aggregation Database (gnomAD), where FIN AF is the Finnish allele frequency and NFEE AF is the non-Finnish-non-Estonian European allele frequency).

^a^The locus spans a genomic region larger than ±1 Mb.

^f^FinnGen-based effect allele frequency (allele frequencies were not available for the genome-wide summary statistics of the previous study^12^).

The table reports distinct loci (more than 1 Mb apart) that contain at least one variant identified to be associated with UL at *p* < 5 × 10^−8^ and that have not been reported in association with UL in prior studies. META-1 is a meta-analysis of 53,534 UL cases and 373,024 female controls from FinnGen limited to the top 10,000 variants of a previously published meta-GWAS of UL^12^, and META-2 is a meta-analysis of 38,466 UL cases and 329,473 female controls from FinnGen and the genome-wide results of the same meta-GWAS^12^ excluding 23andMe data. All significant loci are listed in Tables S1, S2.

**Fig. 3:**
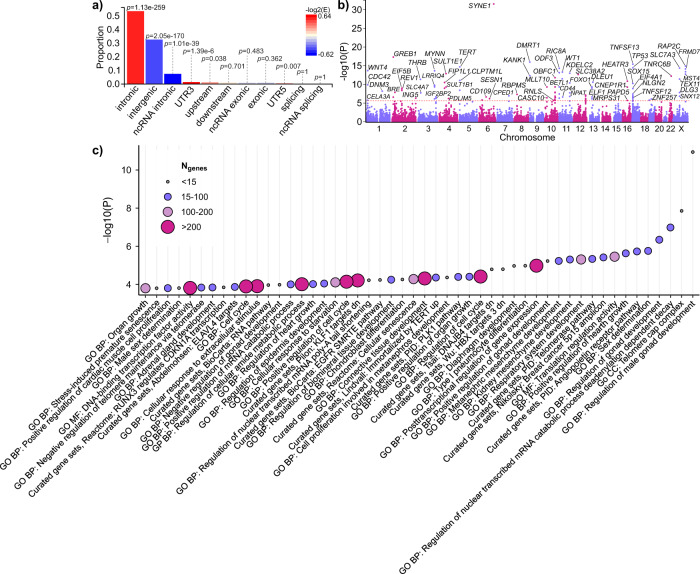
Variant summary and gene set-based results using genome-wide summary statistics from META-2. **a** The proportions of ‘independent genome-wide significant variants’ and ‘variants in LD with independent significant variants’ having corresponding functional annotation. Bars are coloured according to −log_2_(enrichment) relative to all variants in the reference panel. *P* values are obtained using Fisher’s exact test (two-sided). **b** A Manhattan plot of the gene-based test computed by MAGMA^51^. The input variants were mapped to 19,920 protein-coding genes and, thus, significance was considered at *p* < 2.51 × 10^−6^ (0.05/19,920). Purple and pink colours indicate odd and even chromosome numbers, respectively. Thirty-seven gene symbols are omitted. **c** MAGMA^51^ gene-set enrichment analysis was performed for curated gene sets and GO terms available at MsigDB^52^. The plot shows the results for significantly enriched pathways (*p*_FDR_ < 0.05). All data plotted in Fig. 3a–c were produced using FUMA^49^.

Characterisation of the genome-wide results of META-2 suggested that the key UL-associated variants were mostly intronic (Fig. 3a and Supplementary Data 1). We also found enrichment in variants located on 3′ untranslated regions, 5′ untranslated regions, and upstream sequences, whereas the proportions of intergenic and non-coding RNA variants were lower than expected by chance (Fig. 3a).

In the conditional association tests conducted using genome-wide results from META-2, we identified secondary signals in altogether 14 loci (Table S2). Multiple signals were detected in some of the loci, including the well-known UL-risk locus on chr13 near ‘forkhead box O1’ (*FOXO1*), in which we observed four secondary signals in addition to the original association. The lead variants of these secondary signals were either intronic (rs7986407, rs9548898 and rs6563799) or intergenic (rs9576914). Of the UL risk loci that had not been identified in prior studies, we detected a secondary signal in chr2 near ‘myocardin-induced smooth muscle cell lncRNA, an inducer of differentiation’ (*MYOSLID*) where an intronic variant (rs7584910) reached genome-wide significance (*p* = 4.75 × 10^−8^) after conditioning the association to the original lead variant (Table S2).

The results of fine-mapping on 146 association signals, including all UL associations in META-1 and META-2 as well as the independent associations observed in the conditional tests, suggested that 6 signals (near Meis homeobox 1 [*MEIS1*], inositol 1,4,5-trisphosphate receptor type 1 [*ITPR1*], spectrin repeat containing nuclear envelope protein 1 [*SYNE1*], forkhead box O1 [*FOXO1*], tumour protein p53 [*TP53*] and minichromosome maintenance 8 homologous recombination repair factor [*MCM8*]) had a single variant in the 99% credible set concordantly in both META-1 and META-2 (Tables S3, S4). Of these, the missense variant rs16991615 in *MCM8*, the 3′UTR variant rs78378222 in *TP53*, and the intron variant rs117245733 in LINC00598 near *FOXO1* have been reported previously^12^. The remaining variants, i.e. rs17631680 near *MEIS1*, rs3804984 near *ITPR1* and rs58415480 near *SYNE1* are intergenic or intronic variants with no strong evidence of regulatory consequences according to RegulomeDB^23^ and, thus, the association-driving mechanism remains unclear. In addition, 6 secondary signals were found to have a single variant in the 99% credible set (Table S5), all of which were intronic/intergenic.

### Description of the key loci

Previous GWAS findings have indicated that genetic factors altering pathways involved in oestrogen signalling, Wnt signalling, transforming growth factor (TGF)-β signalling, and cell cycle progression are associated with UL risk^10–19^. The loci identified in this study further underscore the involvement of pathways regulating SMC proliferation in the modulation of UL risk. Many of these pathways are interrelated: for example, both oestrogen and progesterone increase the secretion of Wnt ligands from myometrial or leiomyoma SMC, which promotes cell proliferation and tumorigenesis via activation of β-catenin^24^. Steroid hormones also influence the production of ECM via signalling through the TGF-β family of ligands and receptors that are highly expressed in multiple fibrotic conditions and contribute to the fibrotic phenotype seen in UL^25^. We identified multiple loci with potential candidate genes functioning in one or more of these pathways, and, in the following, we describe some of our key findings with a focus on loci involved in the regulation of SMC proliferation.

A central finding is an association at 17p12 harbouring myocardin (*MYOCD*; Tables S1,S2). Myocardin is a transcription factor expressed in smooth muscle tissues, including most prominently arteries and colon, but also the uterus (Fig. S22), and it is required for SMC differentiation^26^. The expression of myocardin has been shown to be downregulated in UL tissue compared with normal myometrium^27^. Also, it has been proposed that the loss of myocardin function may be a key factor in driving SMC proliferation in UL^27^; however, prior to our findings, only one study^9^ has reported GWAS association implicating myocardin. The lead variants near *MYOCD* are intergenic variants with no strong evidence of altered regulatory consequences (Table S6), and, thus, a possible association-driving mechanism remains inconclusive. We identified another myocardin-related UL risk association at 2q33.3 near *MYOSLID*, a transcriptional target of myocardin^28^ (Fig. S3)—this locus has not been reported in prior studies. The association lead variant (rs10804157) is a regulatory variant (Table S7) altering the binding of multiple transcription factors (Table S8), including Fos proto-oncogene (FOS) that has been shown to be downregulated in UL^29^. To add yet another example of a myocardin-related UL risk locus, a well-established association at 22q13.1^10,12,15,16^ locates near ‘myocardin-related transcription factor A’ (*MRTFA*; also known as *MKL1*), a gene interacting with myocardin.

Others have suggested that loss of myocardin function may account for the differentiation defects of human leiomyosarcoma cells during malignant transformation^30^: downregulation of myocardin resulted in lower expression of cyclin-dependent kinase inhibitor 1 A (*CDKN1A*; also known as p21), a mediator of cell cycle G1 phase arrest, which facilitated cell cycle progression. The lead variants of the UL association at 6p21.2^9^ near *CDKN1A* locate in an intergenic region with possible regulatory consequences (Table S9). Previous evidence suggests that *CDKN1A* is among the genes, the expression of which correlates with UL size^31^. The UL association at 20q13.31^9^ harbours RNA binding motif protein 38 (*RBM38*; Fig. S14) that binds to and regulates the stability of *CDKN1A* transcripts^32^. In this locus, the UL risk-increasing rs13039273-C is associated with lower *RBM38* expression in the ovary (*p* = 8.7 × 10^−6^; Fig. S23; nominal significance in the uterus, *p* = 2.2 × 10^−3^). Interestingly, oestrogen receptor (ER)α has been shown to inhibit the expression of myocardin^27^, suggesting that the ability of myocardin-CDKN1A-signalling to inhibit cell cycle progression may be impaired in tissues enriched with ERα. Taking together our findings and previous evidence, it seems highly probable that downregulation of myocardin-CDKN1A signalling increases the risk of UL.

### Enrichment for genes regulating development, growth, and cellular senescence

In a gene-based association test, we identified 97 genes associated with UL risk (Fig. 3b and Supplementary Data 1) that were enriched for 50 curated gene sets and/or Gene Ontology (GO) terms (Fig. 3c and Table S10). These included multiple terms related to developmental processes such as, most notably, gonad development (‘regulation of male gonad development’, false discovery rate (FDR)-corrected *p* value (*p*_FDR_) = 1.78 × 10^−7^; ‘regulation of gonad development’, *p*_FDR_ = 0.0017; ‘positive regulation of gonad development’, *p*_FDR_ = 0.0065; ‘negative regulation of gonad development’, *p*_FDR_ = 0.042) but also others, including the development of kidney, respiratory system, biomineral tissue, and adrenal gland (‘kidney mesenchyme development’, *p*_FDR_ = 0.0063; ‘metanephric mesenchyme development’, *p*_FDR_ = 0.0065; ‘cell proliferation involved in metanephros development’, *p*_FDR_ = 0.028; ‘respiratory system development’, *p*_FDR_ = 0.0063; ‘diaphragm development’, *p*_FDR_ = 0.0097; ‘regulation of biomineral tissue development’, *p*_FDR_ = 0.031; ‘adrenal gland development’, *p*_FDR_ = 0.049). UL-associated genes were also enriched for the regulation of cell cycle and senescence (‘regulation of cell cycle’, *p*_FDR_ = 0.028; ‘positive regulation of cell cycle’, *p*_FDR_ = 0.034; ‘cell cycle’, *p*_FDR_ = 0.048; ‘cellular senescence, *p*_FDR_ = 0.031; ‘stress-induced premature senescence’, *p*_FDR_ = 0.049). Enrichment for a curated gene set ‘RUNX3 regulates CDKN1A transcription’ (*p*_FDR_ = 0.049) provided further evidence that *CDKN1A*-related signalling may play a key role in UL. In addition, four of the terms, namely ‘positive regulation of hearth growth’ (*p*_FDR_ = 0.0052), ‘positive regulation of organ growth’ (*p*_FDR_ = 0.028), ‘regulation of hearth growth’ (*p*_FDR_ = 0.041), and ‘organ growth’ (*p*_FDR_ = 0.049) indicated enrichment for genes that function in processes activating growth rate and increasing the size or mass of organs and heart in particular.

### Gene expression colocalization and mediation effects

**Fig. 4:**
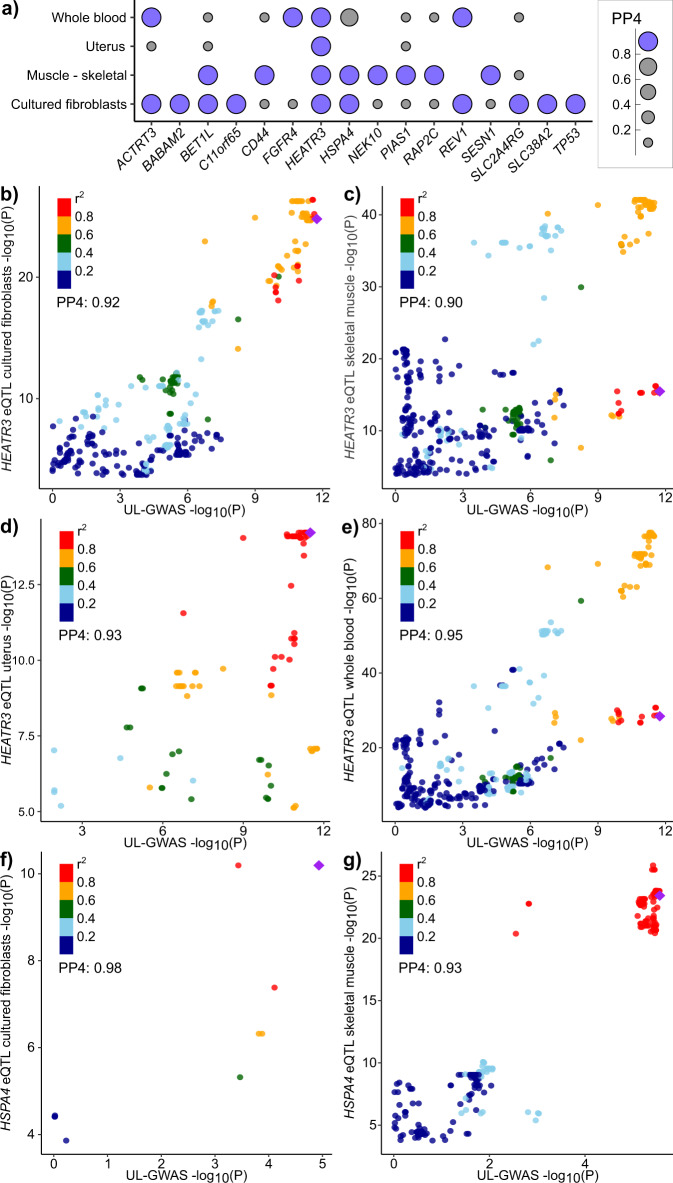
Colocalizations between UL-GWAS signals and eQTL signals. We estimated approximate Bayes factor colocalizations of UL association signals from META-2 and gene expression in GTEx v8^62^ (cultured fibroblasts, skeletal muscle, uterus, and whole blood) using coloc.abf function from the coloc R library^54^. Altogether 92 genes, including the genes closest to the association lead variant at each UL-associated locus and biologically plausible candidate genes, when different from the closest genes, were included in the analysis (Table S2). The figure illustrates **a** all genes, the expression of which colocalizes with UL signal (posterior probability for a single causal variant [PP4] >0.8) in at least one of the studied tissues, as well as colocalization signals for *HEATR3* in **b** cultured fibroblasts, **c** skeletal muscle, **d** uterus, and **e** whole blood, and for *HSPA4* in **f** cultured fibroblasts, and **g** skeletal muscle.

Expectedly, the strongest positive relationships between the expression of UL-associated genes and disease-gene associations were seen in the uterus and cervix (Fig. S24). We found evidence of the colocalization of UL signals with gene expression of 16 genes in one or more of the four studied tissues (posterior probability (PP) for a shared variant ≥0.8; Fig. 4a and Supplementary Data 1,  4). At 16q12.1, a well-known UL risk locus, the UL association signal colocalized with the expression of *HEATR3* in all studied tissues (PP_cultured fibroblasts_ = 0.92; PP_skeletal muscle_ = 0.90; PP_uterus_ = 0.93; PP_whole blood_ = 0.95; Fig. 4b–e). Of the loci that had not been described in association with UL in prior studies, the association signal at 5q31.1 colocalized with the expression of heat shock protein family A (Hsp70) member 4 (*HSPA4*) in cultured fibroblasts (PP = 0.98) and skeletal muscle (PP = 0.93; Fig. 4f, g). Previous studies have shown HSPA4 to associate with ERα and thus to play a role in oestrogen signalling^33^ as well as to enhance the angiogenesis ability of vessel endothelial cells in placenta accreta, a condition where the placenta grows too deeply in the uterine wall^34^. Both oestrogen signalling^35^ and angiogenic growth factor dysregulation^36^ are also involved in UL, which makes *HSPA4* a highly plausible candidate to drive the UL association at 5q31.1. We further tested if the UL-risk associations are mediated by gene expression in the significant loci. The results of the mediation tests were mostly inconclusive, and we found no genome-wide significant mediation effects that would have passed the test for heterogeneity in dependent instruments (HEIDI; *p*_HEIDI_ ≥0.05) (Supplementary Data 5−8). In our study, the previously reported result suggesting that the expression of *HEATR3* mediates UL risk association at 16q12.1^11^ reached genome-wide significance in whole blood (*p* = 1.49 × 10^−9^), skeletal muscle (*p* = 4.78 × 10^−9^), and transformed fibroblasts (*p* = 3.21 × 10^−8^) but none of the mediation effects passed the HEIDI test (respective *p* values: *p*_HEIDI.whole.blood_ = 2.71 × 10^−27^, *p*_HEIDI.skeletal.muscle_ = 2.51 × 10^−25^, *p*_HEIDI.transformed.fibroblasts_ = 5.71 × 10^−10^).

### Genetic correlations with metabolic and anthropometric traits

**Fig. 5:**
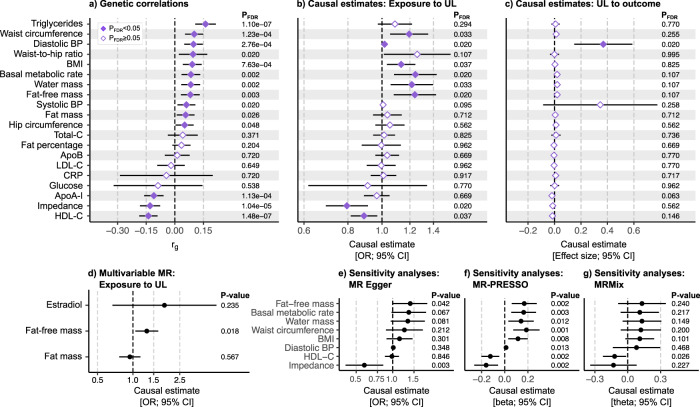
Genetic correlations and causal relationships between uterine leiomyomata and metabolic and anthropometric traits. We estimated **a** genetic correlations (*r*_g_) between uterine leiomyomata (UL) and 20 metabolic and anthropometric traits using UL-GWAS data from META-2 (*n* = 367,903) and summary statistics for other traits as provided by the MRC Integrative Epidemiology Unit (IEU) GWAS database (*n* ranges from 33,231 to 757,601; the trait-specific sample sizes are provided in Table S11). The analysis software was LDSC^21^. To dissect the causal relationships, we performed bi-directional two-sample Mendelian randomisation (MR) implemented in the TwoSampleMR R library^60,63^; the plots (**b**, **c**) show the causal estimates obtained using the inverse variance-weighted (IVW) method. We further estimated **d** the multivariable effects of whole-body fat-free mass, whole-body fat mass, and estradiol level on UL risk using the same TwoSampleMR R library^60,63^. In sensitivity analyses, we derived causal estimates using **e** MR Egger (as implemented in TwoSampleMR), **f** outlier-corrected MR-PRESSO^43^, and **g** MRMix^44^ methods for the traits showing a significant IVW-based causal effect on UL. For all MR analyses, genetic instruments for UL were extracted from the GWAS completed in FinnGen (*n* = 123,579) and for other, mostly UKBB-based, traits from the MRC IEU GWAS database (*n* ranges from 33,231 to 757,601; Table S11) except for estradiol, for which the instruments were extracted from a study by ref. ^61^. (*n* = 206,927). In all Mendelian randomisation analyses, LD pruning was completed using a European population reference, the threshold of *r*^2^ = 0.001, and a clumping window of 10 kb. False discovery rate (FDR)-corrected^64^ *p* values <0.05 were considered significant in primary analyses (**a**–**c**). Multivariable MR and sensitivity analyses (**d**–**g**) were considered exploratory, and no multiple testing correction was applied. The error bars represent the corresponding 95% confidence intervals (CI). Numerical details are provided in Tables S12–S15, and scatter plots and the results of the leave-one-out analyses are shown in Figs. S25, 26 and S28–35, respectively.

We used LDSC software^21^ to evaluate the genetic correlations (*r*_g_) of UL with 20 metabolic and anthropometric traits (Fig. 5a, Table S11, and Supplementary Data 1). In line with previous observational studies reporting associations between cardiometabolic risk factors and UL risk^20,37^, we found UL to show a positive genetic correlation with serum triglyceride level (*r*_g_ = 0.161, *p*_FDR_ = 1.10 × 10^−7^), waist circumference (*r*_g_ = 0.101, *p*_FDR_ = 1.23 × 10^−4^), diastolic blood pressure (*r*_g_ = 0.098, *p*_FDR_ = 2.76 × 10^−4^), waist-to-hip ratio (*r*_g_ = 0.095, *p*_FDR_ = 0.020), body mass index (BMI; *r*_g_ = 0.091, *p*_FDR_ = 7.63 × 10^−4^), systolic blood pressure (*r*_g_ = 0.061, *p*_FDR_ = 0.020), whole-body fat mass (*r*_g_ = 0.057, *p*_FDR_ = 0.026), and hip circumference (*r*_g_ = 0.051, p_FDR_ = 0.048), and negative genetic correlation with concentrations of high-density lipoprotein cholesterol (HDL-C; *r*_g_ = −0.139, *p*_FDR_ = 1.48 × 10^−7^) and apolipoprotein A-I (ApoA-I; *r*_g_ = −0.110, *p*_FDR_ = 1.13 × 10^−4^). Somewhat unexpectedly, we found UL to be closely genetically correlated with basal metabolic rate (*r*_g_ = 0.084, *p*_FDR_ = 0.002), whole-body water mass (*r*_g_ = 0.083, *p*_FDR_ = 0.002), and whole-body fat-free mass (*r*_g_ = 0.083, *p*_FDR_ = 0.003). Compatible with these findings, UL showed a negative genetic correlation with the impedance of whole-body (*r*_g_ = −0.130, *p*_FDR_ = 1.04 × 10^−5^) (i.e. a bioelectrical measure used for estimating body composition; higher muscle mass leads to lower impedance). Compared with whole-body fat mass, the genetic correlations of UL with these anthropometric traits indicating good physical health (i.e. basal metabolic rate, water mass, and fat-free mass) tended to be more robust in terms of both larger *r*_g_ values and smaller *p* values.

### Causal evidence underscores the involvement of altered muscle tissue biology

To further evaluate the causal relations between UL and the same 20 metabolic and anthropometric traits, we applied bi-directional two-sample Mendelian randomisation. Regarding circulating lipids, we found higher HDL-C to be causally associated with a lower risk of UL (inverse variance-weighted [IVW] method-based odds ratio [OR] = 0.89 [0.82, 0.97], *p*_FDR_ = 0.037; Fig. 5b, Table S12, and Supplementary Data 1). There was no evidence of a causal relationship between UL and blood triglyceride level (Fig. 5b) even if, among the studied traits, triglycerides showed the most robust genetic correlation with UL in terms of both *r*_g_ and *p* value (Fig. 5a). Likewise, atherogenic cholesterol measures, total-C and low-density lipoprotein (LDL)-C, and apolipoprotein B (ApoB) concentration were not causally related to UL risk (Fig. 5b).

We found multiple causal associations between anthropometric traits and UL risk (Fig. 5b). Of the traits commonly linked with compromised health, waist circumference (OR = 1.19 [1.05,1.35], *p*_FDR_ = 0.033) and BMI (OR = 1.13 [1.03–1.24], *p*_FDR_ = 0.037) were causally associated with UL risk. Compared with these, the causal associations between UL and traits implying good physical health were somewhat more robust (basal metabolic rate: OR = 1.24 (1.08, 1.43], *p*_FDR_ = 0.020; whole-body water mass: OR = 1.22 [1.06, 1.40], *p*_FDR_ = 0.033; whole-body fat-free mass: OR = 1.24 [1.08, 1.42], *p*_FDR_ = 0.020; impedance of whole body: OR = 0.79 [0.69, 0.91], *p*_FDR_ = 0.020). When considering the null causal effect of whole-body fat mass on UL risk (*p*_FDR_ = 0.712), it seems apparent that the causal effect of BMI on UL arises from the increased lean body mass rather than fat mass.

Taken together, it seems that obesity-related cardiometabolic risk factors may not play a causal role in the pathophysiology of UL even if those are associated with UL risk on a population level^20,37^. Our findings are in line with a previous report suggesting obesity to be causal for uterine endometrial cancer, but not for the other four studied gynaecologic diseases, including UL^38^. Of note, the causal relationship between UL and diastolic blood pressure remained inconclusive as the causal estimate was significant in both directions (Fig. 5b, c and Supplementary Data 1).

UL are considered oestrogen-dependent, and UL have higher ERα expression compared with normal uterine myometria^35^. ERs are expressed in a variety of tissues, including all musculoskeletal tissues^39^. In females, muscle mass and strength are closely coupled with oestrogen status: girls begin to gain muscle mass after the onset of puberty^40^, whereas in older age, during perimenopausal and postmenopausal periods, muscle strength declines considerably^41^. If oestrogen enhances muscle growth^42^, the observed causal relationship between fat-free mass and UL risk could arise secondary to high oestrogen contributing to muscle growth. Therefore, we further tested the multivariable effects of whole-body fat-free mass, whole-body fat mass, and estradiol on UL risk. The results of the multivariable model (Fig. 5d and Table S13) indicate that, among the three traits, only whole-body fat-free mass has a nominally significant causal effect on UL risk (*p* = 0.018) and, thus, support the original findings.

We note that the results of Mendelian randomisation should be interpreted with caution: although we did not observe horizontal pleiotropy (Table S12), the causal estimates were typically heterogenic (Table S12; scatter plots in Figs. S25, 26). Funnel plots did not suggest major asymmetry indicative of directional pleiotropy; however, minor asymmetry due to outliers was present for some exposures (Fig. S27). We further obtained outlier-corrected estimates using MR-PRESSO^43^ (Table S14) and an outlier-robust MRMix method^44^ (Table S15). The results were highly matching to the original findings (Fig. 5f, g), thus providing assurance of the validity of the evidence obtained in the primary analyses. Also, in the leave-one-out sensitivity analyses (Figs. S28–35), all causal estimates were consistently positive (higher fat-free mass was causally associated with a higher risk of UL; Fig. S34) or negative (higher impedance was causally associated with a lower risk of UL; Fig. S32) suggesting that there is no single variant driving the causal associations.

### Strengths and limitations

Compared with the previous UL GWASs, our study had a larger sample size, which facilitated discoveries of multiple association signals at loci that had not been described in prior studies and also confirmed a high number of previously reported UL risk loci. Importantly, careful manual curation of the biological function of the genes in the UL-associated loci was highly beneficial in providing an understanding of UL-related biology. Due to the limitations in data availability, we needed to conduct two distinct meta-analyses to maximise the sample size in META-1 (including 23andMe but limited to the top 10,000 variants from the previous study^12^) and to obtain genome-wide results in META-2 (including genome-wide data from the previous study^12^ but excluding 23andMe). Multiple analyses conducted downstream of the GWAS provided further insights into the key genetic pathways. We found only minimal evidence suggesting that the UL risk associations would be mediated by gene expression; it must be acknowledged, however, that the currently available gene expression data is limited in terms of the number of relevant tissue samples (the number of samples with genotype data is only 129 for the uterus in GTEx Analysis Release V8) and the low statistical power may interfere the discovery of significant effects. Regarding the multivariable Mendelian randomisation, the genetic instruments for estradiol are weaker than the instruments for body composition measures, which may contribute to poor statistical power to detect a causal effect—it would be beneficial to reassess the multivariable effects once a larger estradiol GWAS, preferably conducted in females, will be available potentially providing stronger instruments for MR. Given that our work only includes computational approaches, further functional studies would be warranted to provide molecular evidence for our findings. Finally, the replication of our findings in other non-European ethnicities would be of high value.

## Discussion

The numerous UL risk loci identified in the present study provide valuable insights into the architecture of heritable genetic risk factors in UL. Multiple aspects of our study, including the results of gene-based enrichment analyses and LDSC regression-derived genetic correlations, indicate altered muscle tissue biology in UL. Most notably, Mendelian randomisation-based evidence suggesting a causal relationship between genetic tendency to accumulate fat-free mass and UL risk provides an alternative perspective on UL-related pathophysiology. When considering the oestrogen-dependency of UL, it remains possible that the oestrogen-rich environment, due to sexual maturity, may trigger excess SMC growth resulting in UL in women who are genetically inclined to build up muscle.

Currently, the only essentially curative treatment for UL is hysterectomy, which underscores the high demand for the development of alternative effective therapies^2^. The herein presented results provide several potential targets for translational research to develop pharmacologic interventions for UL. Therapies targeted at myocardin-CDKN1A signalling or, considering the causal evidence, other factors regulating muscle growth may hold the greatest potential.

## Methods

Our research complies with all relevant ethical regulations. FinnGen participants provided written informed consent for biobank research, based on the Finnish Biobank Act. Alternatively, older research cohorts, collected before the start of FinnGen (in August 2017), were collected based on study-specific written informed consents and later transferred to the Finnish biobanks after approval by Fimea, the National Supervisory Authority for Welfare and Health. Recruitment protocols followed the biobank protocols approved by Fimea. The Coordinating Ethics Committee of the Hospital District of Helsinki and Uusimaa (Helsingin ja Uudenmaan Sairaanhoitopiiri, HUS) approved the FinnGen study protocol Nr HUS/990/2017. The FinnGen study is approved by Finnish Institute for Health and Welfare (Terveyden ja hyvinvoinnin laitos, THL), approval number THL/2031/6.02.00/2017, amendments THL/1101/5.05.00/2017, THL/341/6.02.00/2018, THL/2222/6.02.00/2018, THL/283/6.02.00/2019, THL/1721/5.05.00/2019, Digital and population data service agency VRK43431/2017−3, VRK/6909/2018-3, VRK/4415/2019-3 the Social Insurance Institution (Kansaneläkelaitos, KELA) KELA 58/522/2017, KELA 131/522/2018, KELA 70/522/2019, KELA 98/522/2019, and Statistics Finland TK-53-1041-17. The Biobank Access Decisions for FinnGen samples and data utilised in FinnGen Data Freeze 5 include THL Biobank BB2017_55, BB2017_111, BB2018_19, BB_2018_34, BB_2018_67, BB2018_71, BB2019_7, BB2019_8, BB2019_26, Finnish Red Cross Blood Service Biobank 7.12.2017, Helsinki Biobank HUS/359/2017, Auria Biobank AB17-5154, Biobank Borealis of Northern Finland_2017_1013, Biobank of Eastern Finland 1186/2018, Finnish Clinical Biobank Tampere MH0004, Central Finland Biobank 1-2017 and Terveystalo Biobank STB 2018001.

### Study populations

FinnGen (www.finngen.fi/en) is a public-private partnership project launched in 2017 with an aim to improve human health through genetic research. The project utilises genome information from a nationwide network of Finnish biobanks that are linked with digital health records from national hospital discharge (available from 1968), death (1969-), cancer (1953-) and medication reimbursement (1995-) registries using the unique national personal identification codes. Ultimately, the data resource will cover roughly 10% of the Finnish population. We studied data from 123,579 female participants (18,060 UL cases and 105,519 female controls) from FinnGen Preparatory Phase Data Freeze 5. UL cases were required to have an entry of ICD-10: D25, ICD-9: 218, or ICD-8: 21899, and participants who had no records of these entries were deemed as controls. The mean age at the diagnosis was 46.8 years.

FibroGENE is a consortium of conventional, population-based and direct-to-consumer cohorts that was assembled to replicate and identify UL risk variants. In the study by Gallagher et al., they studied data from 35,474 UL cases and 267,505 female controls, including participants from four population-based cohorts (Women’s Genome Health Study, WGHS; Northern Finland Birth Cohort, NFBC; QIMR Berghofer Medical Research Institute, QIMR; the UK Biobank, UKBB) and one direct-to-consumer cohort (23andMe). Detailed descriptions of cohorts and sample selections are available in Supplementary Methods of the original publication^12^.

### Genotyping, imputation, and quality control

In FinnGen, genotyping of the samples was performed using Illumina and Affymetrix arrays (Illumina Inc., San Diego, and Thermo Fisher Scientific, Santa Clara, CA, USA). Sample quality control (QC) was performed to exclude individuals with high genotype missingness (>5%), ambiguous gender, excess heterozygosity (±4 SD) and non-Finnish ancestry. Regarding variant QC, all variants with low Hardy–Weinberg equilibrium (HWE) *p* value (<1e-6), high missingness (>2%) and minor allele count (MAC) <3 were excluded. Chip genotyped samples were pre-phased with Eagle 2.3.5 with the number of conditioning haplotypes set to 20,000. Genotype imputation was carried out by using the Finnish population-specific SISu v3 reference panel with Beagle 4.1 (version 08Jun17.d8b) as described in the following protocol: dx.doi.org/10.17504/protocols.io.nmndc5e. In post-imputation QC, variants with imputation INFO <0.6 were excluded.

Genotyping and subsequent imputation and QC procedures conducted in the previous study have been described in detail elsewhere^12^. Shortly, in four of the study populations, namely WGHS, NFBC, QIMR and 23andMe, genotyping was performed using Illumina or Affymetrix platforms, and individuals with a genotyping call rate <0.98 were excluded from the study. Imputation was performed using the reference panel from the 1000 Genomes Project European data Phase 3. In variant QC, variants with call rates of <99% or with deviation from HWE equilibrium (*p* < 1 × 10^−6^) were excluded. UKBB data QC and imputation were performed centrally and are described elsewhere^45^. Additional QC filters were applied to exclude poorly imputed (*r*^2^ < 0.4) and rare (minor allele frequency [MAF] <1%) variants^12^.

### Genome-wide associations

The UL GWAS in FinnGen was completed using the Scalable and Accurate Implementation of Generalised (SAIGE) software version 0.36.3.2^46^. The association models were adjusted for age, the first ten genetic principal components, and genotyping batch, and only variants with a minimum allele count of five were included in the analysis.

### Meta-analyses

Two sets of fixed-effect, inverse variance-weighted meta-analyses (implemented in METAL^47^ V.2011-03-25) were performed: the results obtained in FinnGen were meta-analyzed with (1) the top 10,000 most significant variants associating with UL in a published GWAS^12^ (META-1) and (2) the genome-wide summary statistics of the same study^12^. Statistical significance was considered at the standard genome-wide significance level (*p* < 5 × 10^−8^). The genomic inflation factor was estimated using an automated LD score (LDSC) regression pipeline^48^ using the genome-wide results from META-2.

### Characterisation of association signals

We used a web-based platform, FUMA^49^ (accessed on 05/18/2022), to perform functional annotations of the GWAS results: we completed functional gene mapping and gene-based association and enrichment tests using the genome-wide UL associations from META-2 and predefined lead variants as reported in Table S2. FUMA identifies variants showing genome-wide significant association (*p* < 5 × 10^−8^) with the study trait and, among the significant variants, identifies variants in low LD (r^2^ < 0.6) as ‘independent significant variants’ and further identifies variants in LD (r^2^ > 0.6) with the ‘independent significant variants’; ANNOVAR^50^ annotations are performed for all these variants to obtain information on the functional consequences of the key variants. MAGMA^51^, also implemented in FUMA, was used to perform gene-based association testing and gene-set enrichment analyses: gene-based *p* values were computed for protein-coding genes by mapping variants to genes and subsequent enrichment analyses were performed for the significant genes using 4728 curated gene sets and 6166 GO terms as reported in MsigDB^52^.

To further identify the potential UL candidate gene(s) with biologically relevant functions, we annotated all genes within a 1 Mb window from the association lead variant. We explored information provided by GenBank^32^ and UniProt^53^ to determine the functions of the genes. To complement the information available in these databases, a broad literature search was performed to identify previous work published regarding the genes of interest.

We further tested the colocalization of UL association signals and gene expression in GTEx v8 (accessed on 05/19/2022). To do this, we used genome-wide UL associations from META-2 and gene expression data (significant variant-gene pairs) from four tissues: cultured fibroblasts, muscle (skeletal), uterus, and whole blood. Colocalizations were performed per gene for altogether 92 genes covering the gene closest to the association lead variant at each UL-associated locus and biologically plausible candidate genes if different from the closest genes (Table S2). Approximate Bayes Factor (ABF) analyses were completed using ‘coloc.abf’ from the ‘coloc’ R library (5.1.0.1)^54^ with default priors (i.e., *p*_1_ = *p*_2_ = 1 × 10^−4^, *p*_12_ = 1 × 10^−5^). Colocalizations with posterior probability >0.8 for a shared causal variant were considered significant. To test if altered gene expression mediates UL risk associations, we used a method proposed by ref. ^55^ as implemented in Complex Traits Genetics Virtual Lab (CTG-VL; beta-0.4)^56^; we performed these tests using genome-wide UL results from META-2 and tissue-specific gene expression data (GTEx, V7) for cultured fibroblasts, skeletal muscle, uterus, and whole blood. We further used RegulomeDB (2.0.3)^23^ to discover regulatory elements overlapping with the intergenic variants in the genome-wide significant UL risk loci that had not been associated with UL risk in prior studies.

To assess if the UL-associated loci harbour secondary association signals, we performed conditional association tests using Genome-wide Complex Trait Analysis (GCTA) software (1.93.0 beta Linux)^57^ and genome-wide summary statistics from META-2. Here, FinnGen was used as a reference sample to estimate linkage disequilibrium (LD) corrections. The associations were first conditioned on the most significant variant (i.e. the variant with the smallest *p* value) at each genome-wide significant locus, and conditioning was continued until no variant attained *p* < 5 × 10^-8^. Using GCTA, we also conditioned the associations on a nearly 6 Mb region on chromosome 10 to estimate if the association signal near *STN1* spans a genomic region larger than the ±1 Mb locus definition.

To further characterise the loci, we fine-mapped each locus discovered in the two meta-analyses, including all independent association signals discovered in the conditional analyses. We first extracted the summary statistics of each locus, and then applied the FinnGen fine-mapping pipeline (available at https://github.com/FINNGEN/finemapping-pipeline, accessed on 2/2/2022) with default parameters. In brief, the pipeline calculates linkage disequilibrium within the regions of interest with LDstore2^58^ using FinnGen samples, generates 99% credible sets using the SUm of Single Effects (SuSiE)^59^ and provides a summary of the results.

### SNP-based heritability and genetic correlations

The SNP-based heritability (h^2^_SNP_) of UL was estimated using LDSC regression implemented in LDSC software (v1.0.1)^21^ and genome-wide summary statistics from META-2. A population prevalence of 0.30 (as in ref. ^12^) and a sample prevalence of 0.11 were used to estimate h^2^_SNP_ on the liability scale. In addition, SNP-based heritability was also estimated using SumHer^22^ software (ldak5.2.linux), genome-wide summary statistics from META-2, the pre-computed UK Biobank-based BLD-LDAK model (–tagfile bld.ldak.hapmap.gbr.tagging), and population and sample prevalences of 0.30 and 0.11 as above. The–check-sums option was set to ‘NO’, because only ~2% of the variants present in the tagfile were missing from the META-2 summary statistics. To use the same set of variants in both heritability estimations, insertions and deletions were excluded from these analyses, as SumHer analyses only single nucleotide variations.

We further applied LDSC to estimate genetic correlations (r_g_) of UL with 20 metabolic and anthropometric traits extracted from the GWAS database provided by the MRC Integrative Epidemiology Unit (IEU) (https://gwas.mrcieu.ac.uk/). The 20 traits and their corresponding GWAS-IDs at the MRC IEU database were as follows: apolipoprotein A-I (ApoA-I; ieu-b-107), apolipoprotein B (ApoB; ieu-b-108), basal metabolic rate (ukb-b-16446), body fat percentage (ukb-b-8909), body mass index (BMI; ukb-b-19953), C-reactive protein (CRP; bbj-a-14), diastolic blood pressure (ieu-b-39), fasting blood glucose adjusted for BMI (ebi-a-GCST007858), high-density lipoprotein cholesterol (HDL-C; ieu-b-109), hip circumference (ukb-b-15590), an impedance of whole body (ukb-b-19921), low-density lipoprotein cholesterol (LDL-C; ieu-b-110), systolic blood pressure (ieu-b-38), total cholesterol (ieu-a-301), triglycerides (ieu-b-111), waist circumference (ukb-b-9405), waist-to-hip ratio (ieu-a−72), whole-body fat mass (ukb-b-19393), whole-body fat-free mass (ukb-b-13354) and whole-body water mass (ukb-b-14540).

### Mendelian randomisation

To test for causal inferences between UL and the above-described 20 metabolic and anthropometric traits, we performed bi-directional two-sample Mendelian randomisation. These analyses were completed using ‘TwoSampleMR’ R library (0.5.6)^60^ (https://mrcieu.github.io/TwoSampleMR/). To avoid possible bias from overlapping samples, we extracted genetic instruments for UL from the GWAS results obtained in FinnGen, and for other, mostly UKBB-based traits from the GWAS database provided by the MRC IEU and integrated them into TwoSampleMR. LD pruning was completed using European population reference, a threshold of *r*^2^ = 0.001, and a clumping window of 10 kb, as set as default in ‘clump_data’ function; the numbers of SNPs available for the analyses are listed in Table S12. The inverse variance-weighted (IVW) method was considered the primary analysis. In sensitivity analyses, we derived causal estimates using MR Egger (implemented in TwoSampleMR), MR-PRESSO (1.0)^43^, and MRMix (0.1.0)^44^ methods for the traits showing FDR-significant causal effects on UL in the primary analysis. The sensitivity analyses were conducted using the same sets of instruments that were used in the primary IVW analysis using an identical LD pruning approach. The estimates obtained in the sensitivity analyses were required to be in a matching direction with the IVW estimates to conclude a reliable causal effect. Egger intercepts were evaluated to assess horizontal pleiotropy. Cochran’s Q statistics were derived using ‘mr_heterogeneity’ function to test for heterogeneity. To screen for highly influential variants that could drive the association, for example, due to horizontal pleiotropy, we performed leave-one-out analyses using ‘mr_leaveoneout’ function. We also estimated the multivariable effects of fat-free mass, fat mass, BMI, and estradiol level on UL risk using TwoSampleMR. LD pruning was conducted with the same settings as described above. We used data from FinnGen to extract variant associations with UL, from the MRC IEU GWAS database to extract variant associations with fat-free mass, fat mass, and BMI, and from a Study by ref. ^61^. to extract variant associations with estradiol.

### Reporting summary

Further information on research design is available in the Nature Portfolio Reporting Summary linked to this article.

## Supplementary information

Supplementary Information

Description of Additional Supplementary Files

Supplementary Data 1

Supplementary Data 2

Supplementary Data 3

Supplementary Data 4

Supplementary Data 5

Supplementary Data 6

Supplementary Data 7

Supplementary Data 8

Reporting Summary

## Data Availability

The individual-level data are available under restricted access for legal and ethical reasons. Formal approval for the researchers is required to access the data: please see https://www.finngen.fi/en/access_results for more details. Access to FinnGen GWAS summary statistics can be applied through an online form at https://elomake.helsinki.fi/lomakkeet/102575/lomake.html. Access to individual-level data and genotype data is managed by the Finnish Biobank Cooperative at the Fingenious portal [https://site.fingenious.fi/en/]). The expected response timeframe for access requests to individual-level data is 1-2 months, and the planned account termination date is December 31, 2027. The results of META-1 (UL associations limited to the top 10,000 variants from the previous study) are provided in Supplementary Data 2 and the results limited to the top 10,000 variants from META-2 are provided in Supplementary Data 3. The genome-wide association data generated in this study (META-2) have been deposited in the NHGRI-EBI GWAS Catalogue database under accession code GCST90239856. The summary-level data other than the genetic associations generated in this study are provided in the Supplementary Information. The genome-wide data from the previous UL-GWAS by Gallagher et al. used in this study are available in the NHGRI-EBI GWAS Catalogue database under accession code GCST009158. The genome-wide data of the 20 metabolic and anthropometric traits used in calculating genetic correlations and causal inferences are available at the MRC IEU GWAS database [https://gwas.mrcieu.ac.uk/] (the trait-specific data can be extracted using the trait IDs listed in Table S11).
