# Supplementary material for: Evidence of a causal effect of genetic tendency to gain muscle mass on uterine leiomyomata
Source: Nat Commun. 2023 Feb 1;14:542. doi: 10.1038/s41467-023-35974-7 (PMC9892568; doi:10.1038/s41467-023-35974-7)
Supplement: Supplementary file 1 — Supplementary Information [file 41467_2023_35974_MOESM1_ESM.pdf]

Supplemental Material

Evidence of a causal effect of genetic tendency to gain muscle mass on uterine leiomyomata

Eeva Sliz, Jaakko Tyrmi, Nilufer Rahmioglu, Krina T. Zondervan, Christian M. Becker, FinnGen, Outi Uimari*, Johannes Kettunen*

Content

Figure S1. Regional plot of the novel UL association on chr1 near *EXO1*. 1
Figure S2. Regional plot of the novel UL association on chr1 near *ZBTB18*. 2
Figure S3. Regional plot of the novel UL association on chr2 near *MYOSLID*. 3
Figure S4. Regional plot of the novel UL association on chr3 near *IGF2BP2*. 4
Figure S5. Regional plot of the novel UL association on chr4 near *METAP1* (*EIF4E*, *ADH5*). 5
Figure S6. Regional plot of the novel UL association on chr5 near *HSPA4*. 6
Figure S7. Regional plot of the novel UL association on chr6 near *SESN1*. 7
Figure S8. Regional plot of the novel UL association on chr7 near *NT5C3A*. 8
Figure S9. Regional plot of the novel UL association on chr7 near *CPED1* (*WNT16*). 9
Figure S10. Regional plot of the novel UL association on chr7 near *LINC-PINT*. 10
Figure S11. Regional plot of the novel UL association on chr10 near *SKIDA1* (*DNAJC1*). 11
Figure S12. Regional plot of the novel UL association on chr10 near *RNLS*. 12
Figure S13. Regional plot of the novel UL association on chr11 near *ENSG00000285769*. 13
Figure S14. Regional plot of the novel UL association on chr20 near *CTCFL*/*RBM38*. 14
Figure S15. Regional plot of the novel UL association on chr21 near *RUNX1*. 15
Figure S16. Regional plot of the novel UL association on chr22 near *MYH9*. 16
Figure S17. Regional plot of the novel UL association on chr10 near *KCNMA1* [META-2]. 17
Figure S18. Regional plot of the novel UL association on chr12 near *NUP107* [META-2]. 17
Figure S19. Regional plot of the association signal at 10q24.32 spanning to a 6MB region [META-2]. 18
Figure S20. Quantile-quantile plots of the P-values. 18
Figure S21. Forest plot of the effect estimates of the association lead variants in the novel loci. 19
Figure S22. *MYOCD* expression in tissues. 20
Figure S23. The association of rs13039273-C with *RBM38* expression. 21
Figure S24. Results of MAGMA tissue expression analysis. 22
Figure S25. MR scatter plots: UL as outcome, other traits as exposure. 26
Figure S26. MR scatter plots: UL as exposure, other traits as outcome. 30
Figure S27. Funnel plots of MR causal estimates vs. their precision. 31
Figure S28. Leave-one-out: exposure basal metabolic rate, outcome UL. 32
Figure S29. Leave-one-out: exposure body mass index (BMI), outcome UL. 33
Figure S30. Leave-one-out: exposure diastolic blood pressure, outcome UL. 34
Figure S31: Leave-one-out: exposure HDL cholesterol, outcome UL. 35
Figure S32: Leave-one-out: exposure impedance of whole body, outcome UL. 36
Figure S33: Leave-one-out: exposure waist circumference, outcome UL. 37
Figure S34: Leave-one-out: exposure whole body fat-free mass, outcome UL. 38
Figure S35: Leave-one-out: exposure whole body water mass, outcome UL. 39
Table S1. Genome-wide significant ($p<5x10^{-8}$) loci in META-1. 40
Table S2. Genome-wide significant ($p<5x10^{-8}$) loci in META-2. 41
Table S3. Fine-mapping results of META-1. 43
Table S4. Fine-mapping results of META-2. 44
Table S5. Fine-mapping results of the secondary signals. 45
Table S6. RegulomeDB annotation of the association lead variants near *MYOCD*. 46
Table S7. RegulomeDB annotation of the association lead variants near *MYOSLID*. 47
Table S8. ChIP-seq data of rs10804157. 48
Table S9. RegulomeDB annotation of the association lead variants near *CDKN1A*. 50
Table S10. Results of MAGMA enrichment analysis. 51
Table S11. Genetic correlations of UL with 20 metabolic and anthropometric traits. 52
Table S12. Results of the bi-directional two-sample Mendelian randomization. 53
Table S13. Results of multivariable MR. 57
Table S14. Results of outlier-corrected MR-PRESSO. 58
Table S15. Results of MRMix. 59
References 60

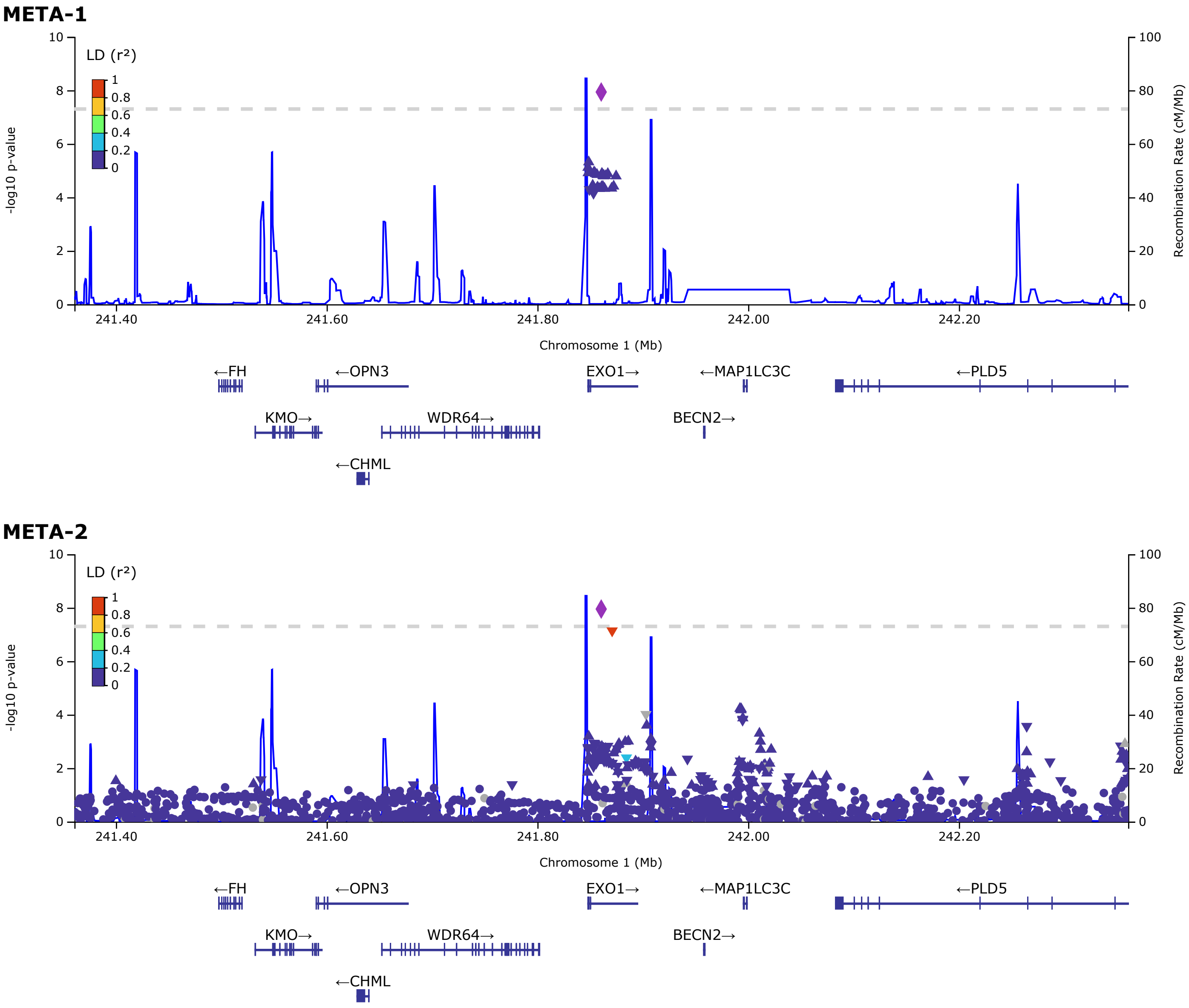

Figure S1. Regional plot of the novel UL association on chr1 near *EXO1*.
The plot on the top shows the result in META-1, and the plot on the bottom shows the result in META-2.

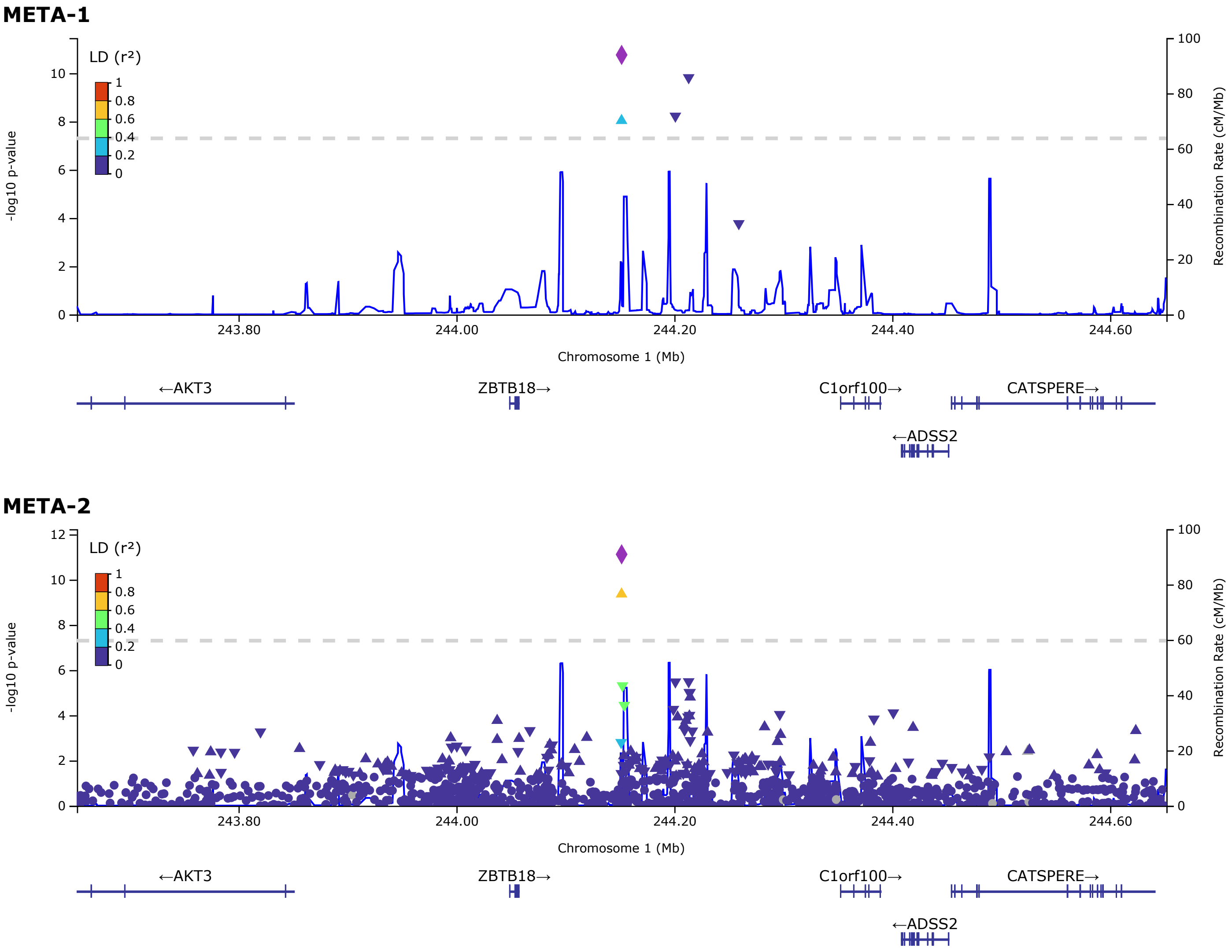

Figure S2. Regional plot of the novel UL association on chr1 near *ZBTB18*.
The plot on the top shows the result in META-1, and the plot on the bottom shows the result in META-2.

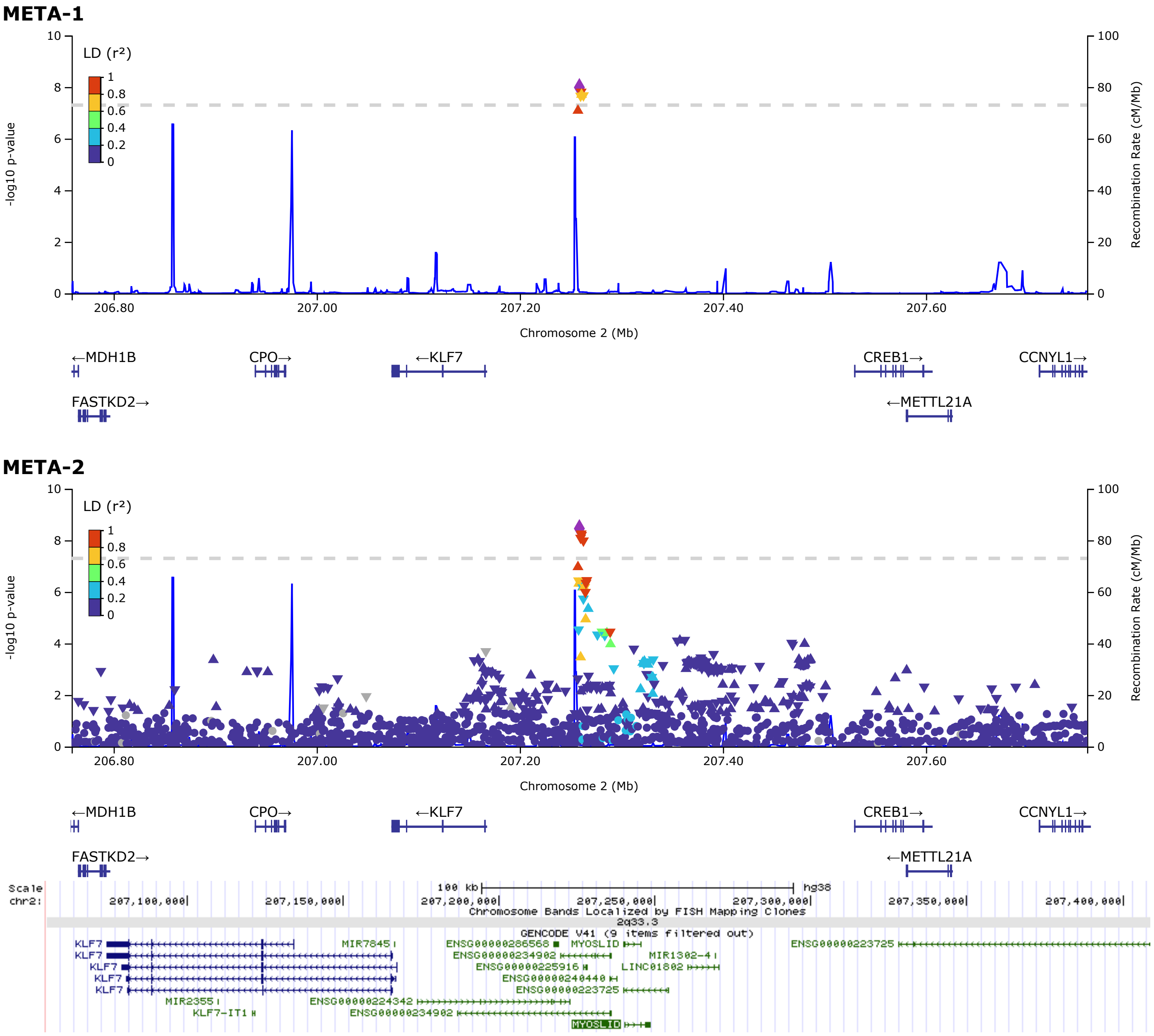

Figure S3. Regional plot of the novel UL association on chr2 near *MYOSLID*.
The plot on the top shows the result in META-1, and the plot in the middle shows the result in META-2. *MYOSLID* location was unavailable for LocusZoom: it locates on chr2: 207,239,811-207,245,887 which is ~78kb downstream from *KLF7*, as indicated in the image extracted from UCSC Genome Browser (bottom).

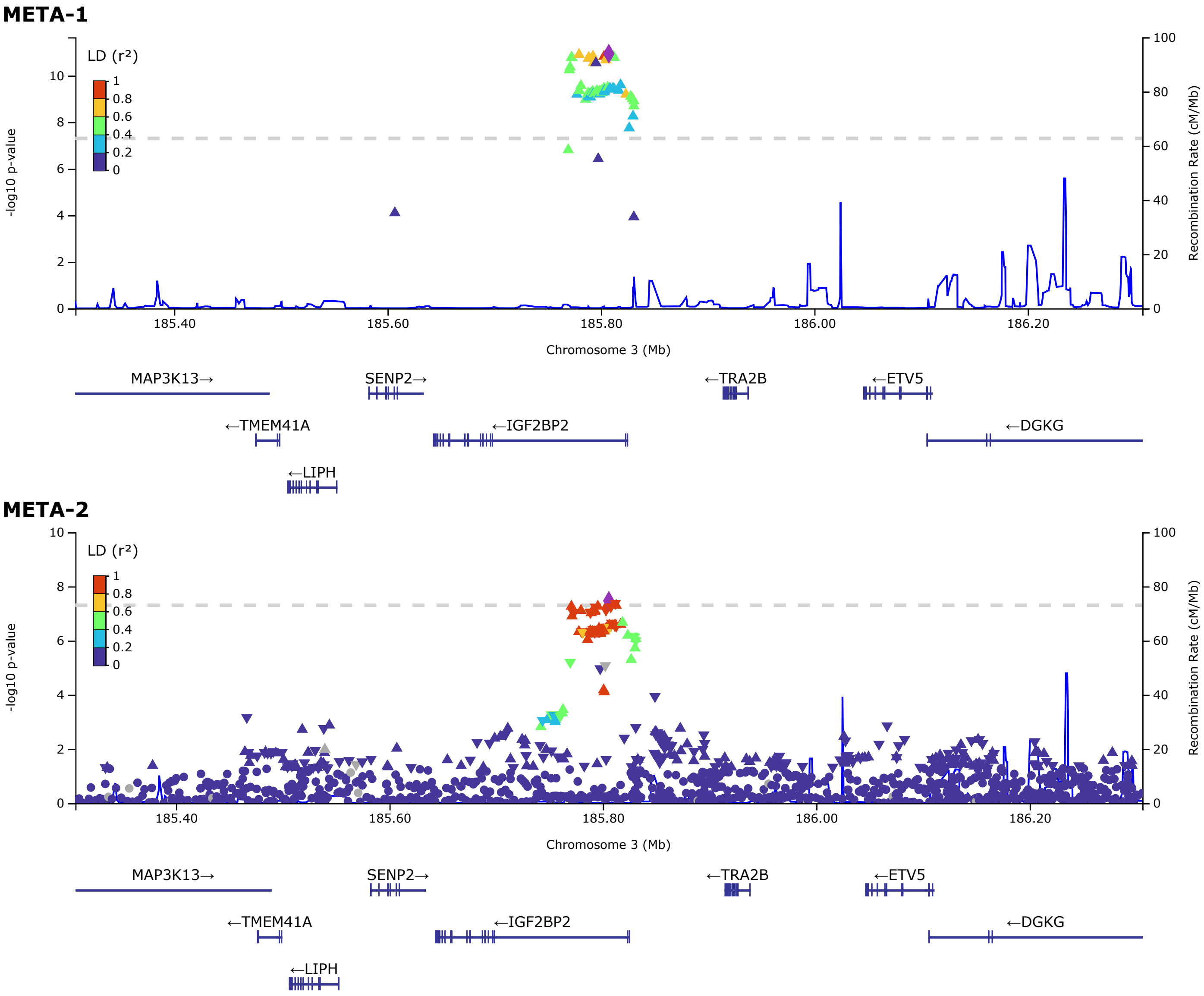

Figure S4. Regional plot of the novel UL association on chr3 near *IGF2BP2*.
The plot on the top shows the result in META-1, and the plot on the bottom shows the result in META-2.

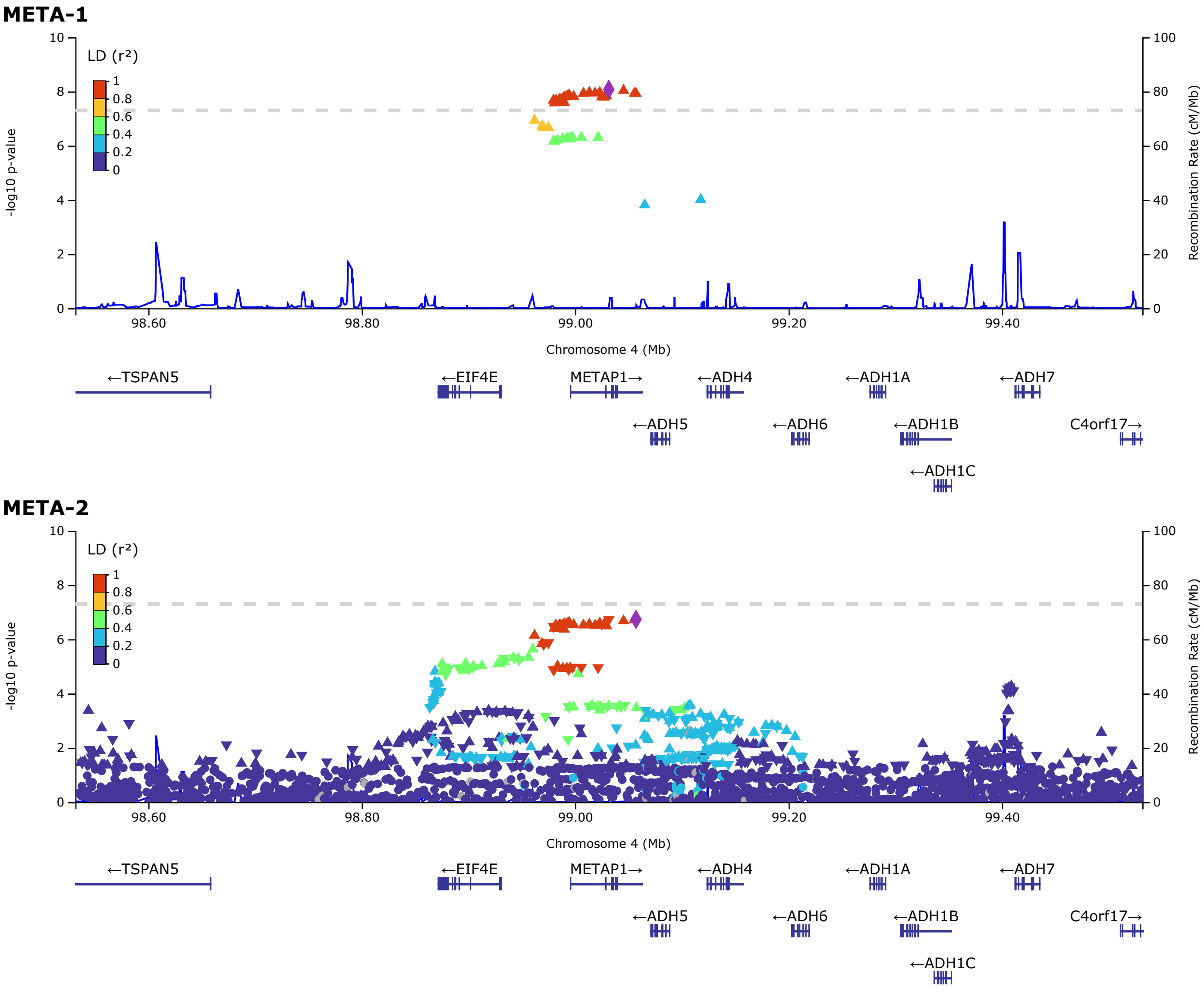

Figure S5. Regional plot of the novel UL association on chr4 near *METAP1* (*EIF4E*, *ADH5*).
The plot on the top shows the result in META-1, and the plot on the bottom shows the result in META-2.

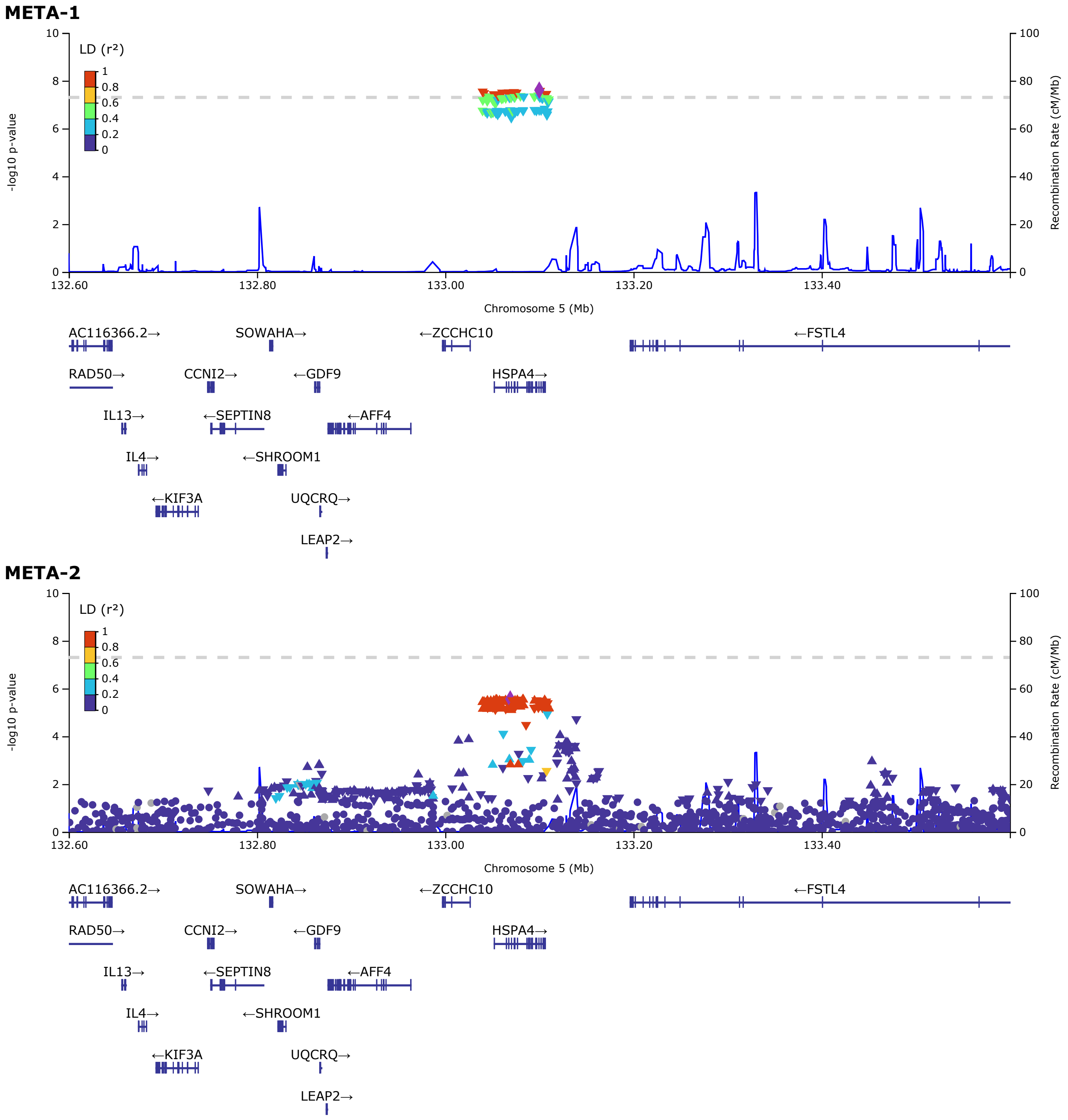

Figure S6. Regional plot of the novel UL association on chr5 near *HSPA4*.
The plot on the top shows the result in META-1, and the plot on the bottom shows the result in META-2.

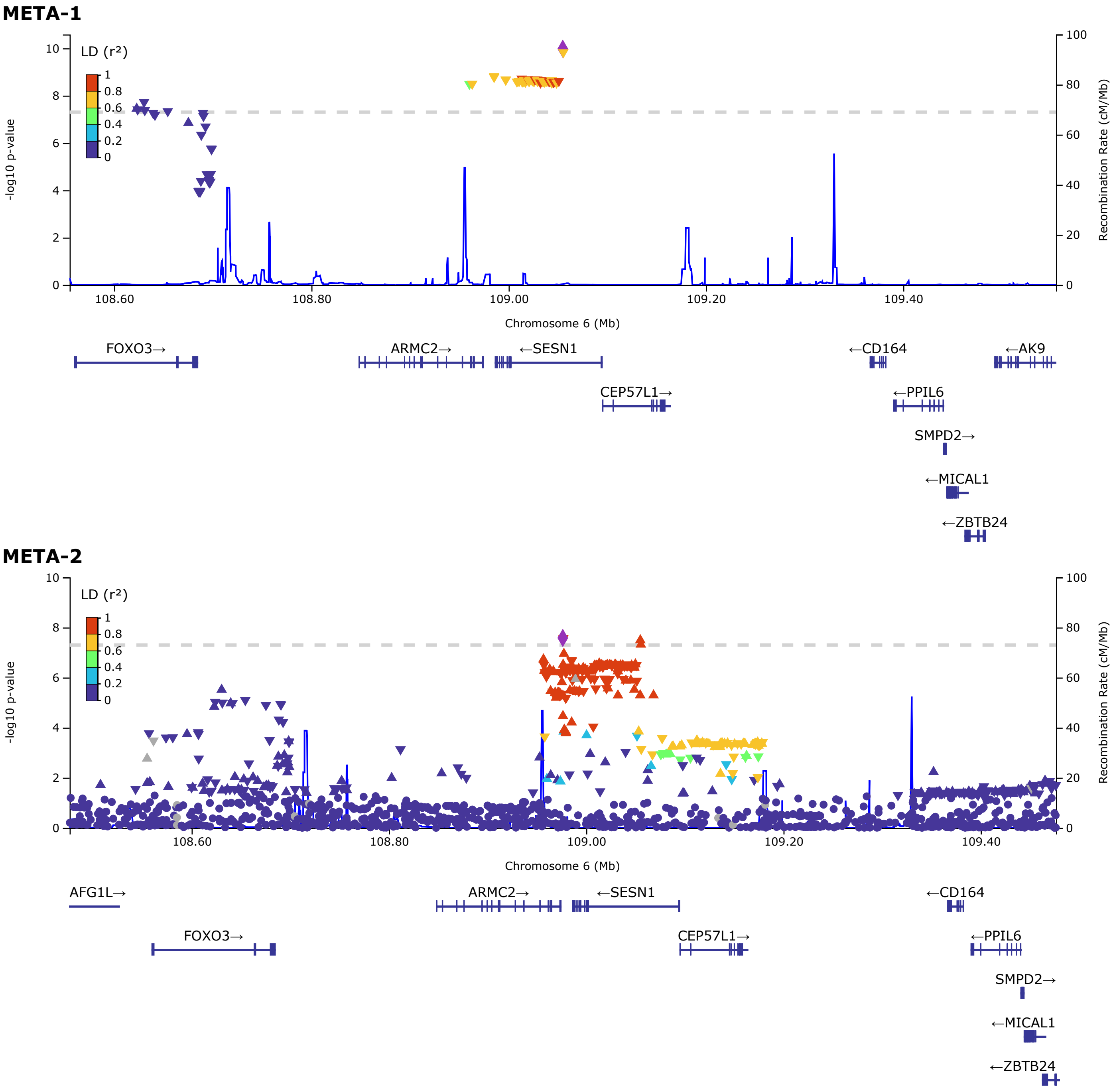

Figure S7. Regional plot of the novel UL association on chr6 near *SESN1*.
The plot on the top shows the result in META-1, and the plot on the bottom shows the result in META-2.

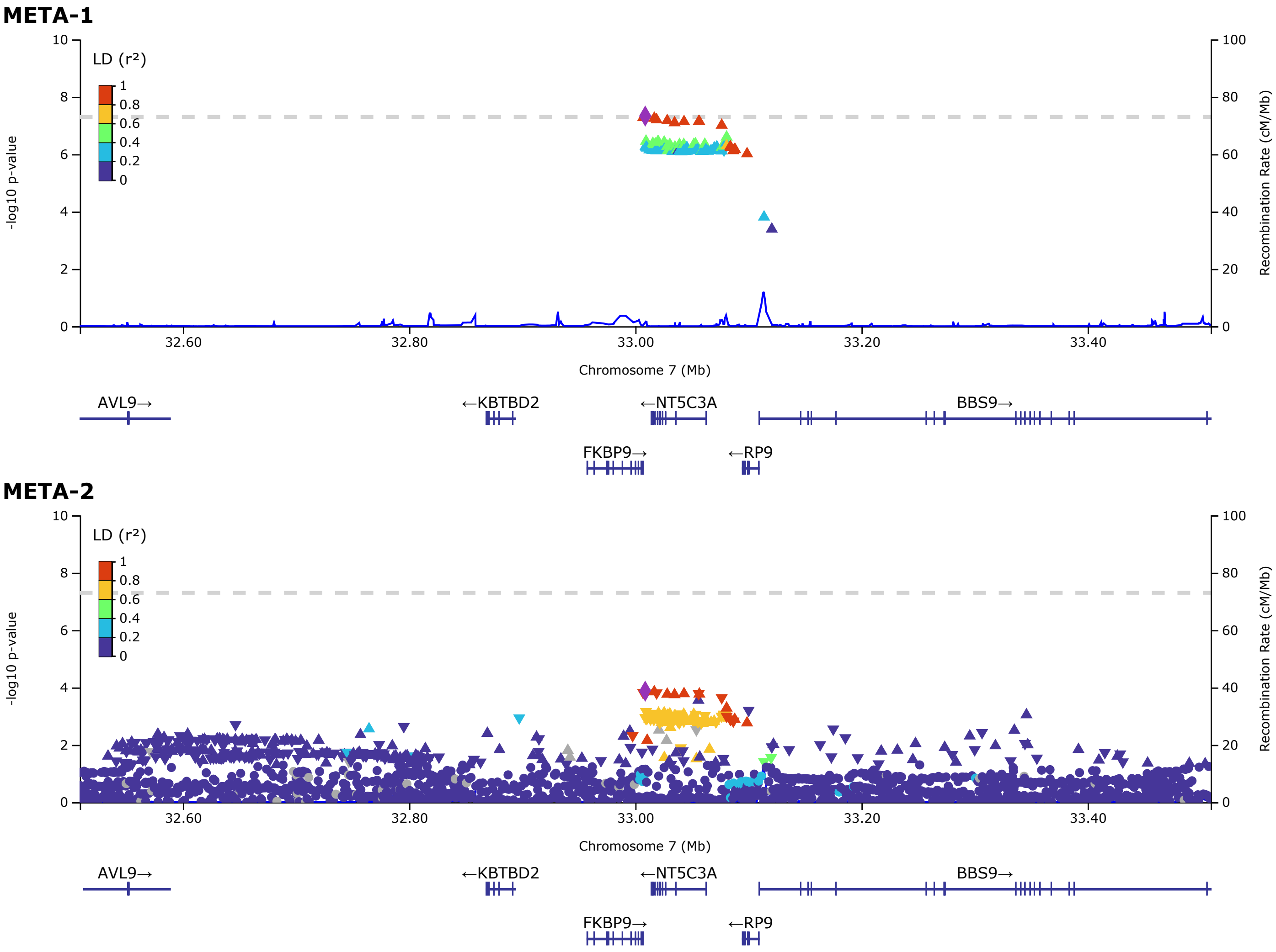

Figure S8. Regional plot of the novel UL association on chr7 near *NT5C3A*.
The plot on the top shows the result in META-1, and the plot on the bottom shows the result in META-2.

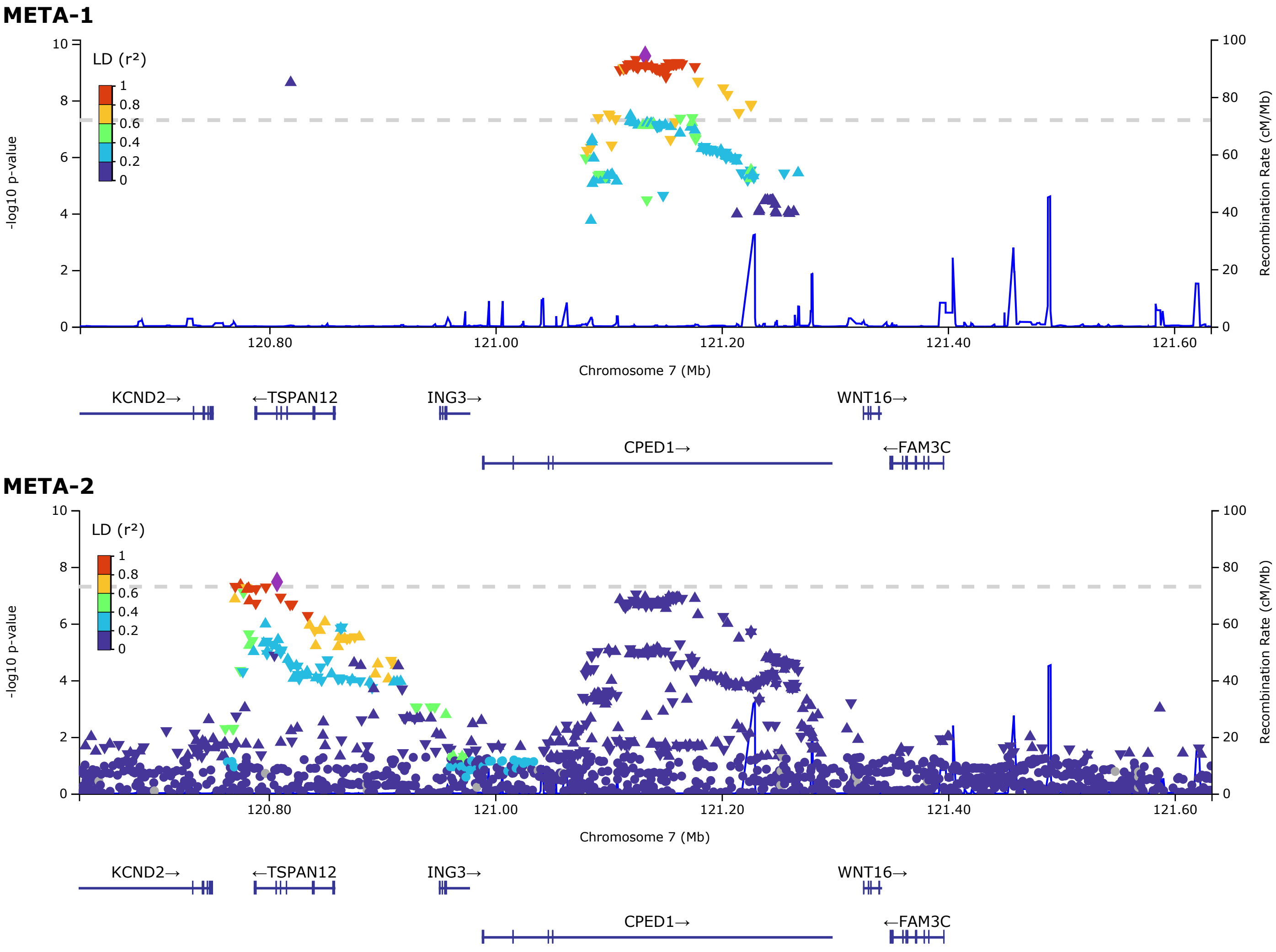

Figure S9. Regional plot of the novel UL association on chr7 near *CPED1* (*WNT16*).
The plot on the top shows the result in META-1, and the plot on the bottom shows the result in META-2.

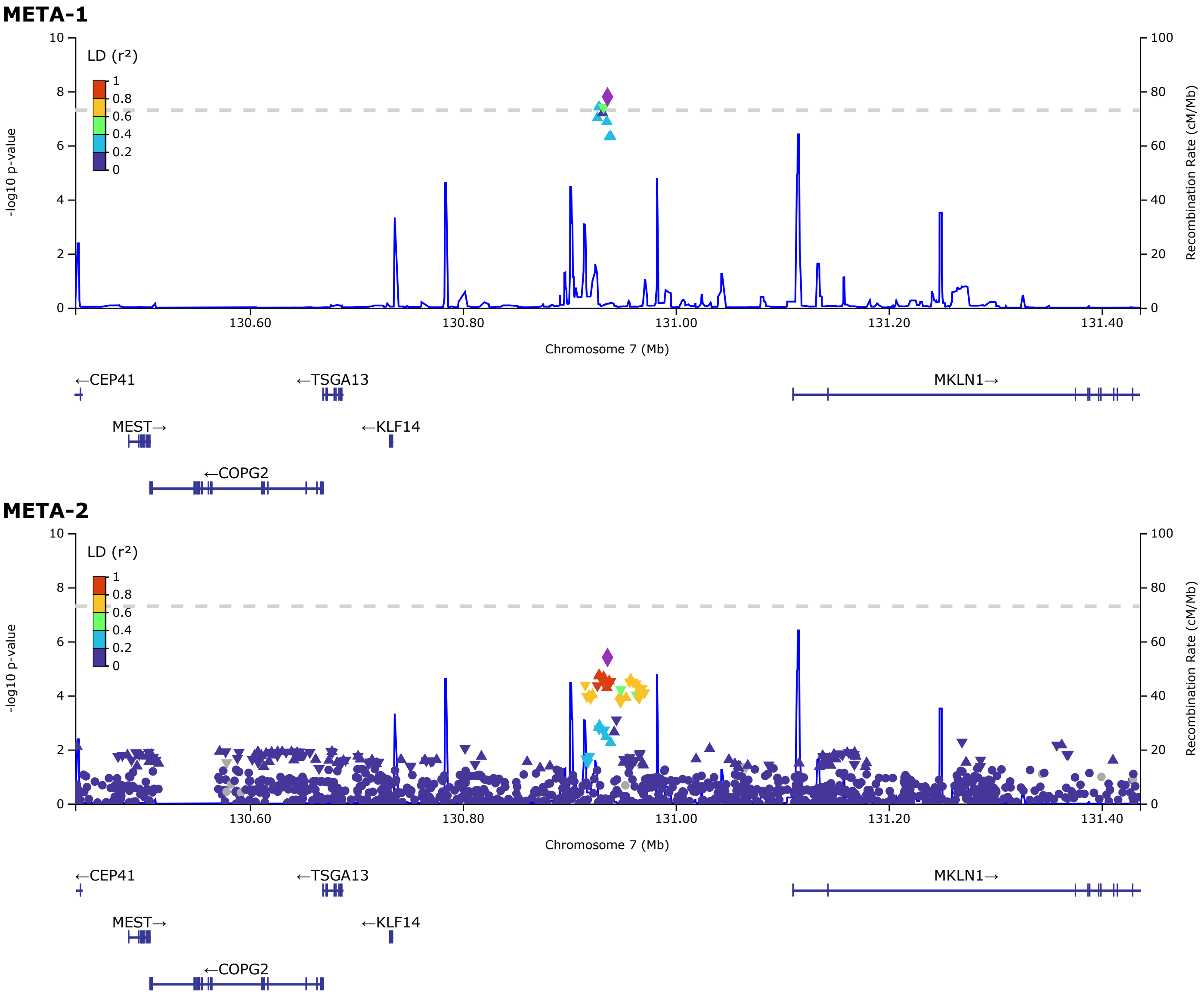

Figure S10. Regional plot of the novel UL association on chr7 near *LINC-PINT*.
The plot on the top shows the result in META-1, and the plot on the bottom shows the result in META-2.

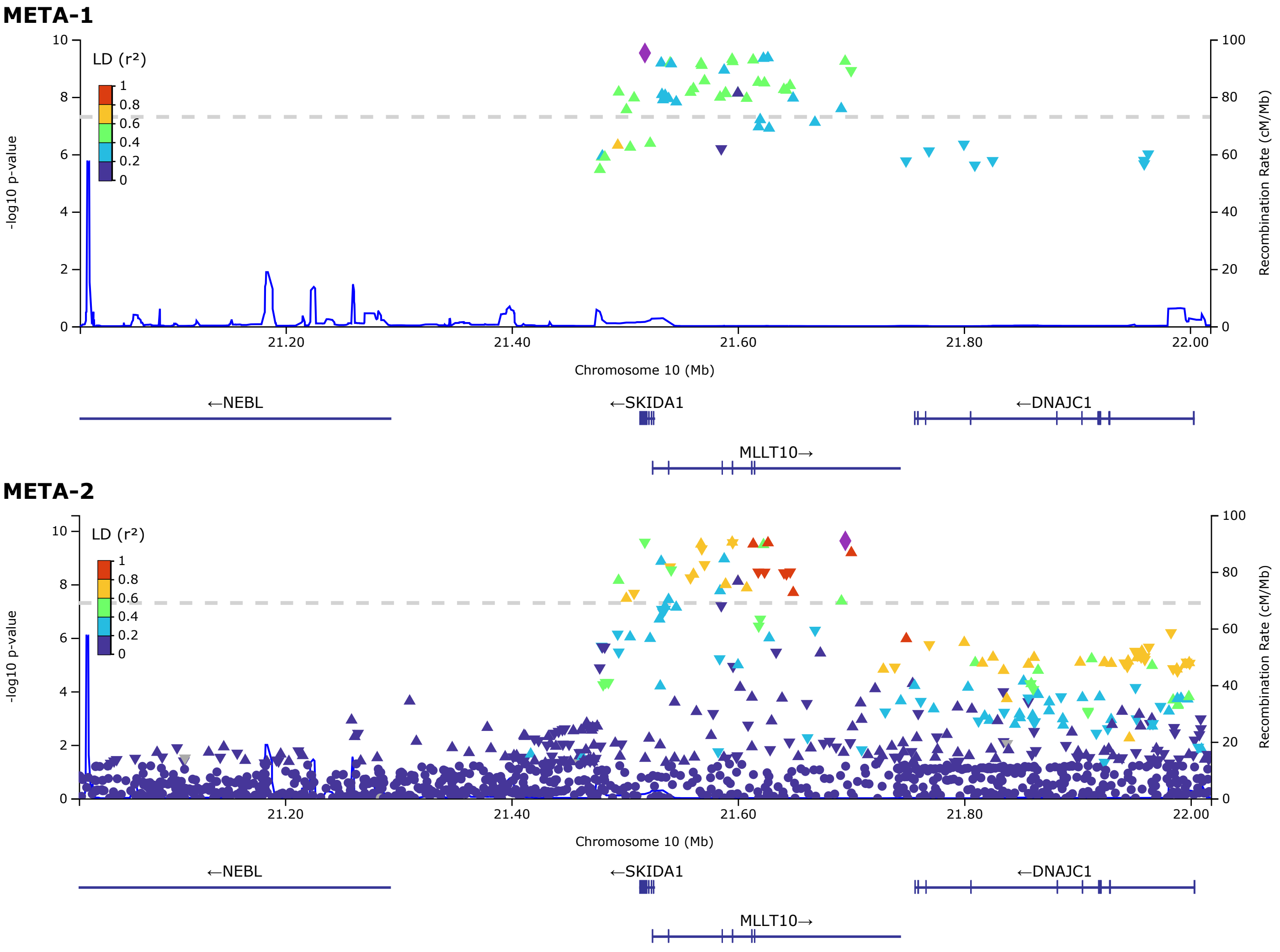

Figure S11. Regional plot of the novel UL association on chr10 near *SKIDA1* (*DNAJC1*).
The plot on the top shows the result in META-1, and the plot on the bottom shows the result in META-2.

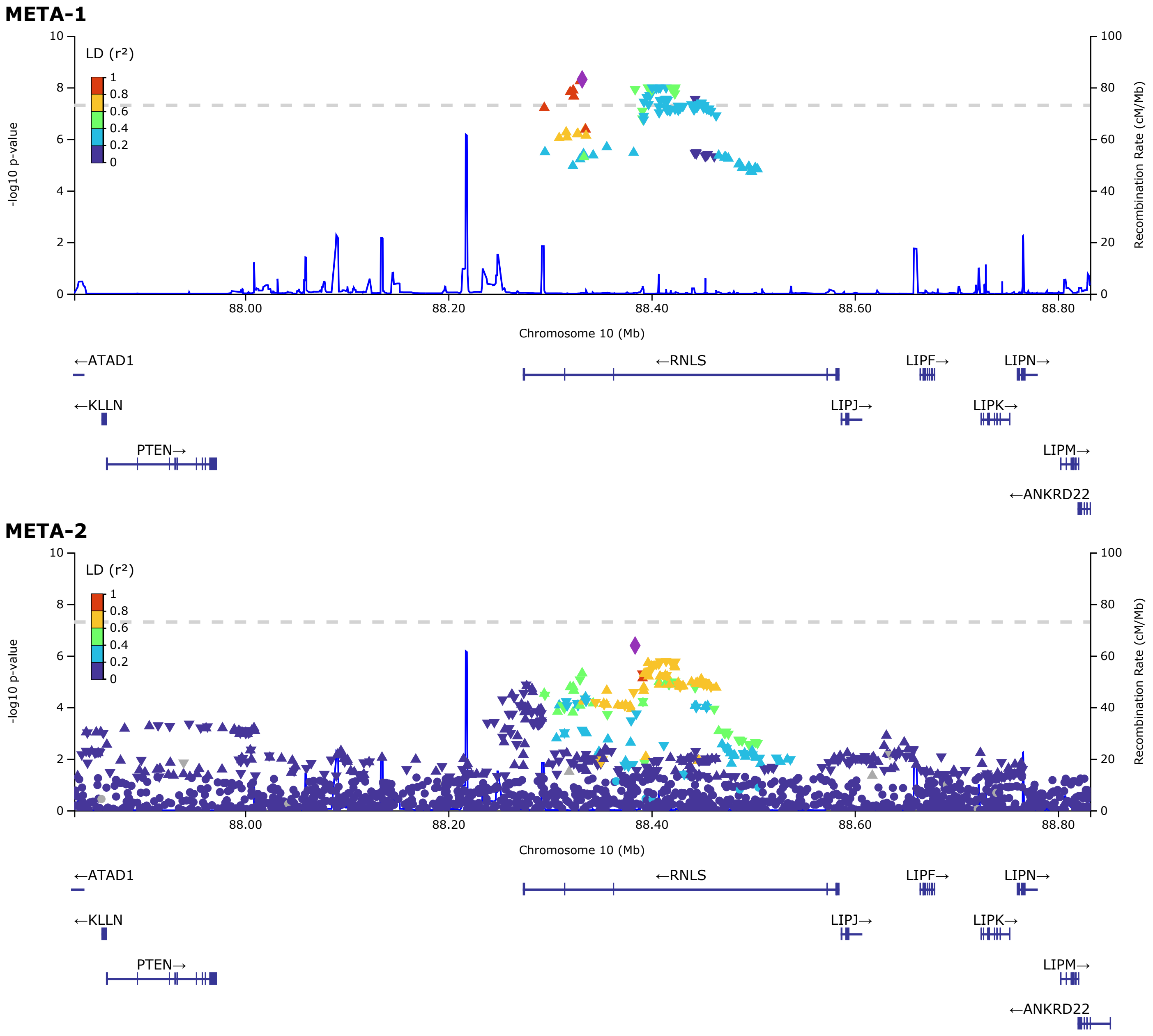

Figure S12. Regional plot of the novel UL association on chr10 near *RNLS*.
The plot on the top shows the result in META-1, and the plot on the bottom shows the result in META-2.

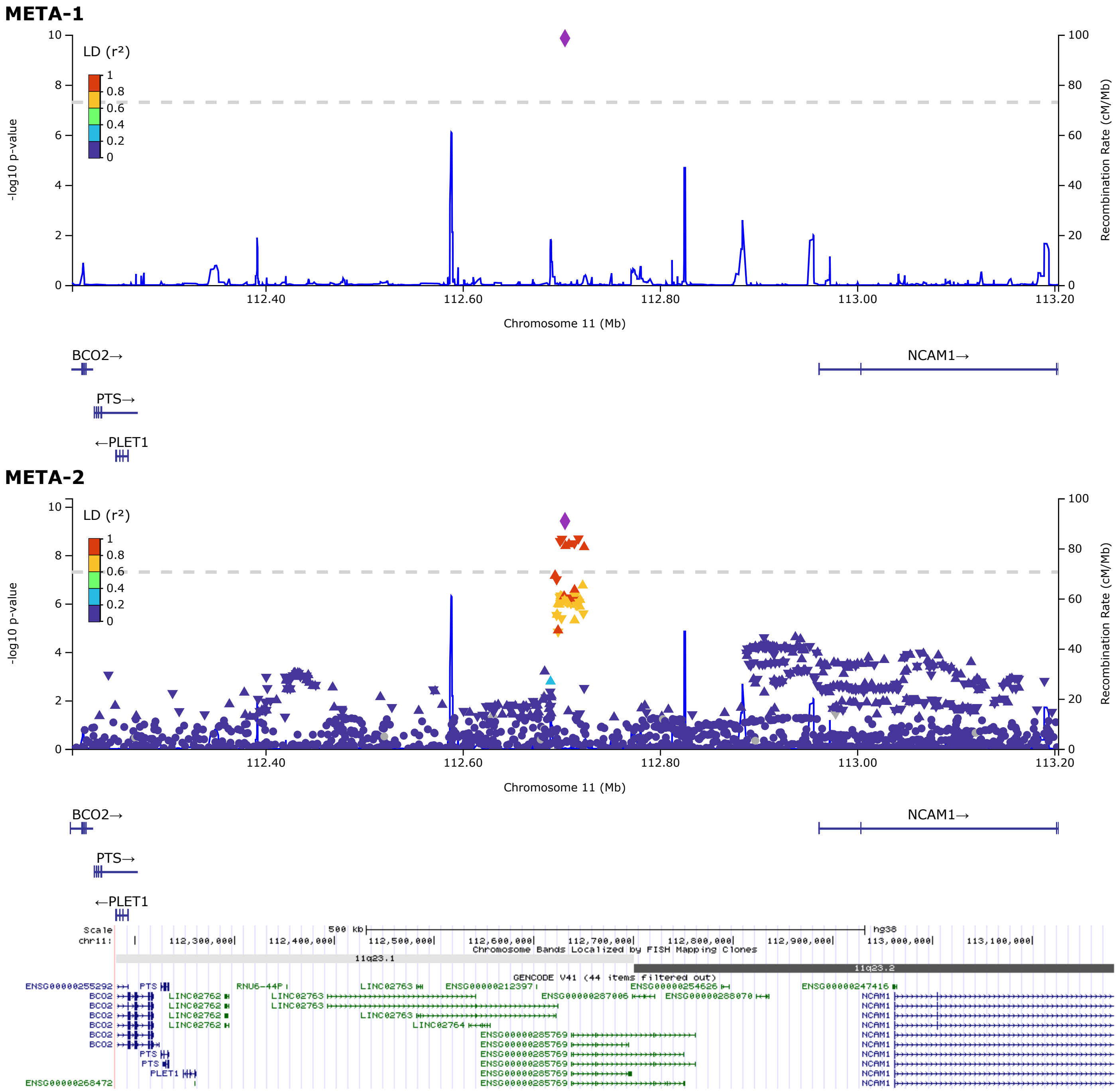

Figure S13. Regional plot of the novel UL association on chr11 near *ENSG00000285769*.
The plot on the top shows the result in META-1, and the plot in the middle shows the result in META-2. ENSG00000285769 location was unavailable for LocusZoom: it locates on chr11:112,637,324-112,761,375 which is ~200kb upstream from *NCAM1*, as indicated in the image extracted from UCSC Genome Browser (bottom).

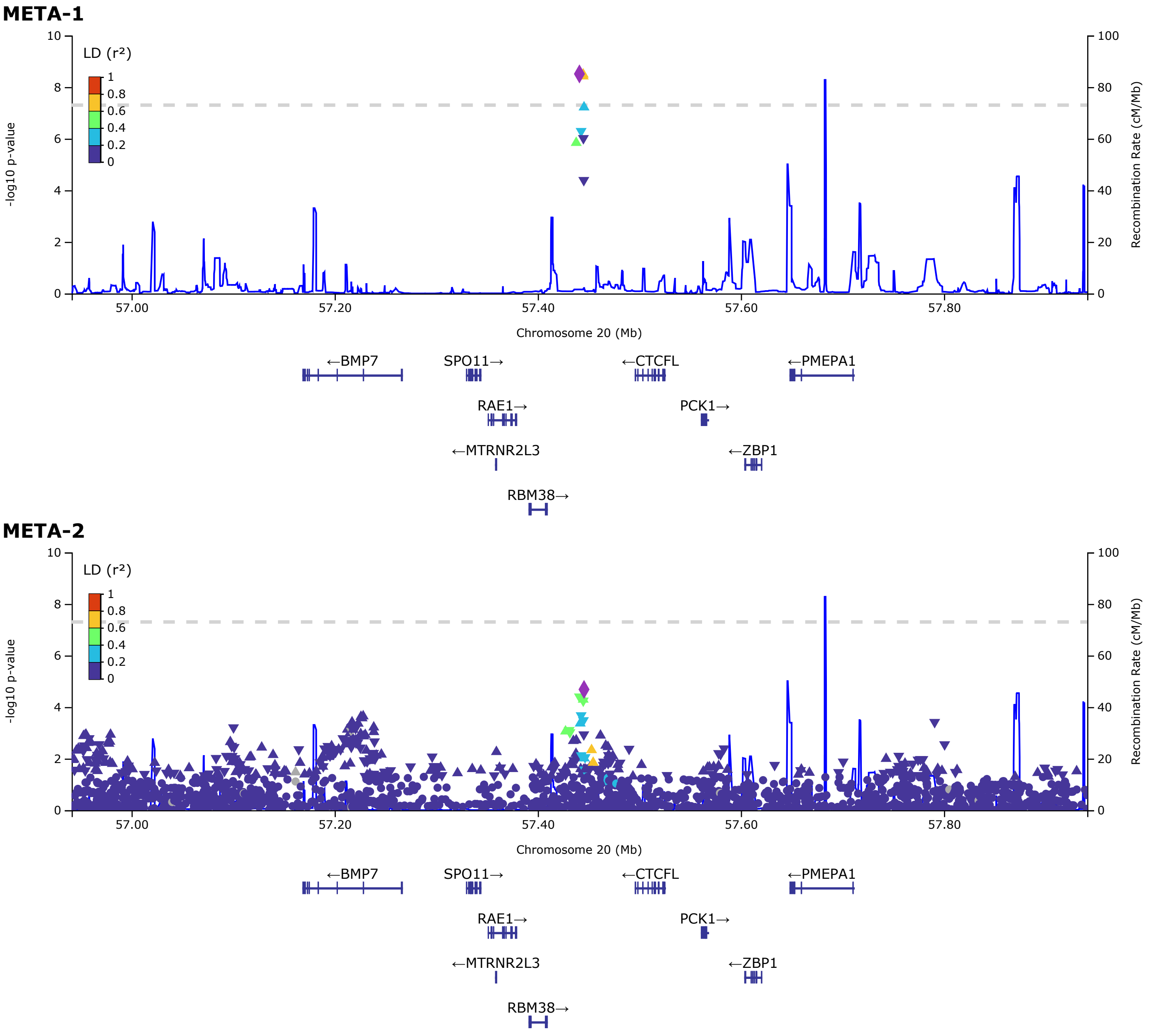

Figure S14. Regional plot of the novel UL association on chr20 near *CTCFL/RBM38*.
The plot on the top shows the result in META-1, and the plot on the bottom shows the result in META-2.

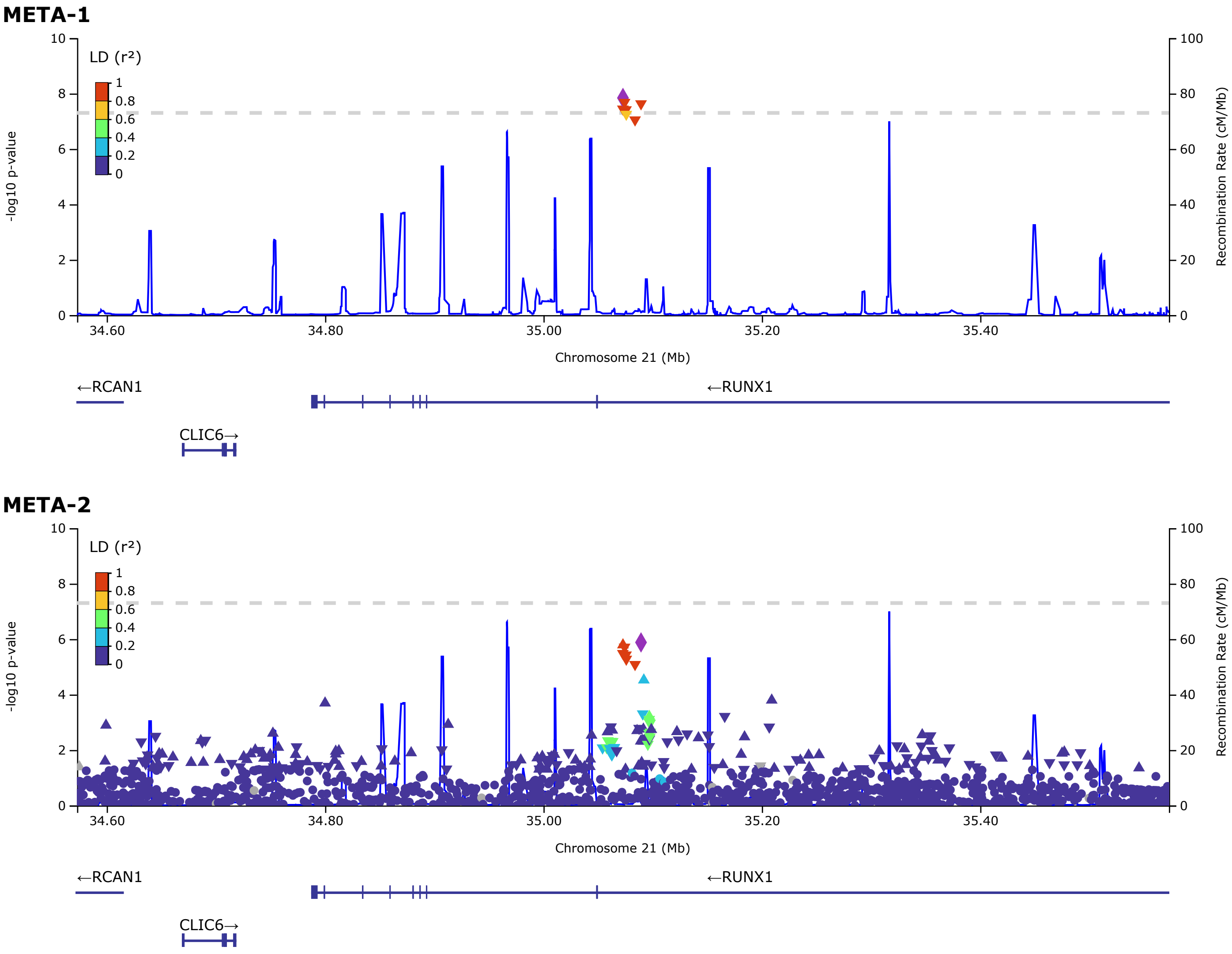

Figure S15. Regional plot of the novel UL association on chr21 near *RUNX1*.
The plot on the top shows the result in META-1, and the plot on the bottom shows the result in META-2.

META-1

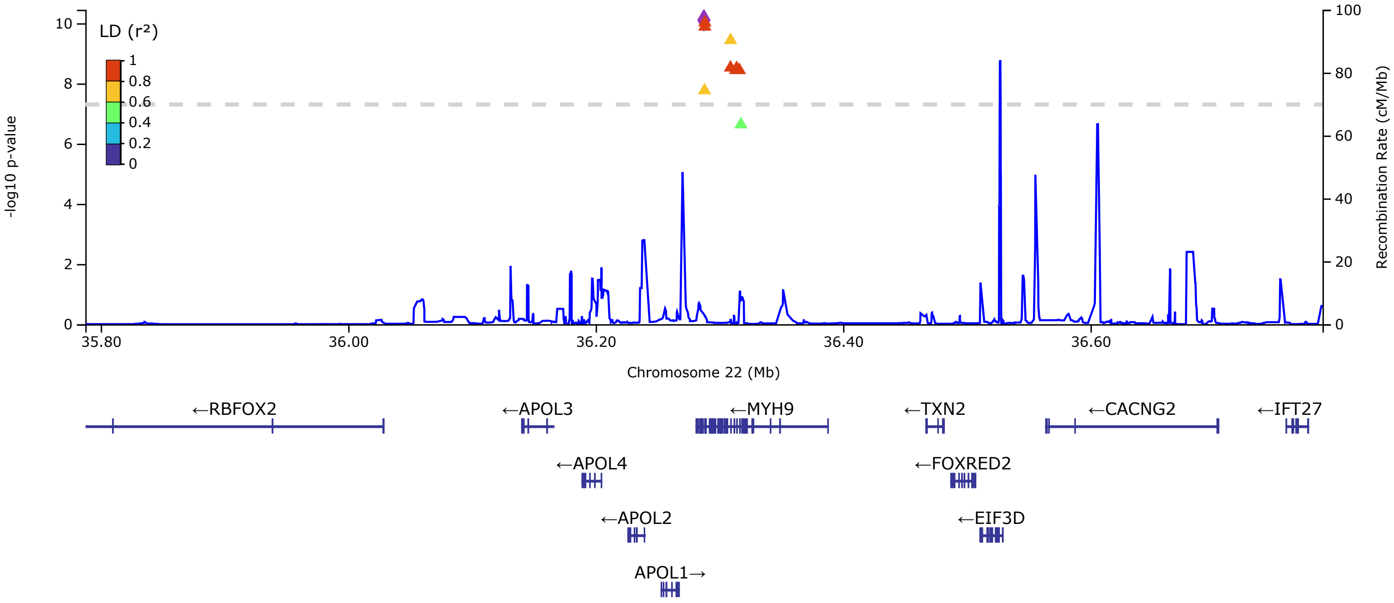

META-2

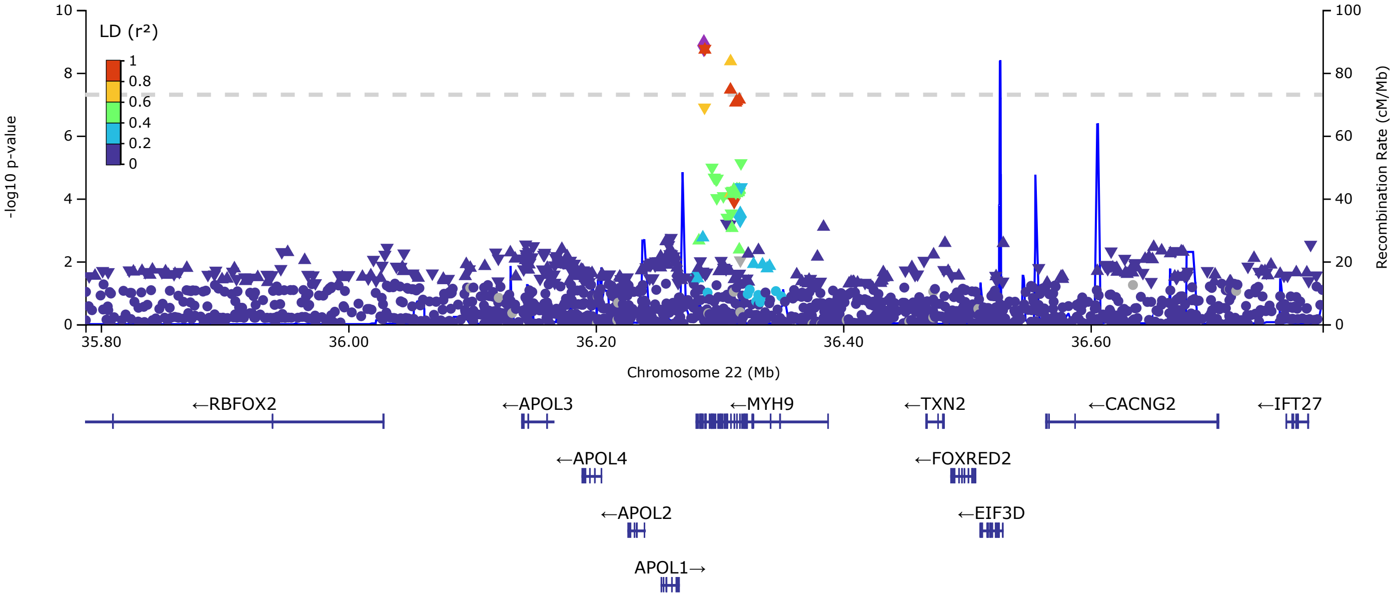

Figure S16. Regional plot of the novel UL association on chr22 near *MYH9*.
The plot on the top shows the result in META-1, and the plot on the bottom shows the result in META-2.

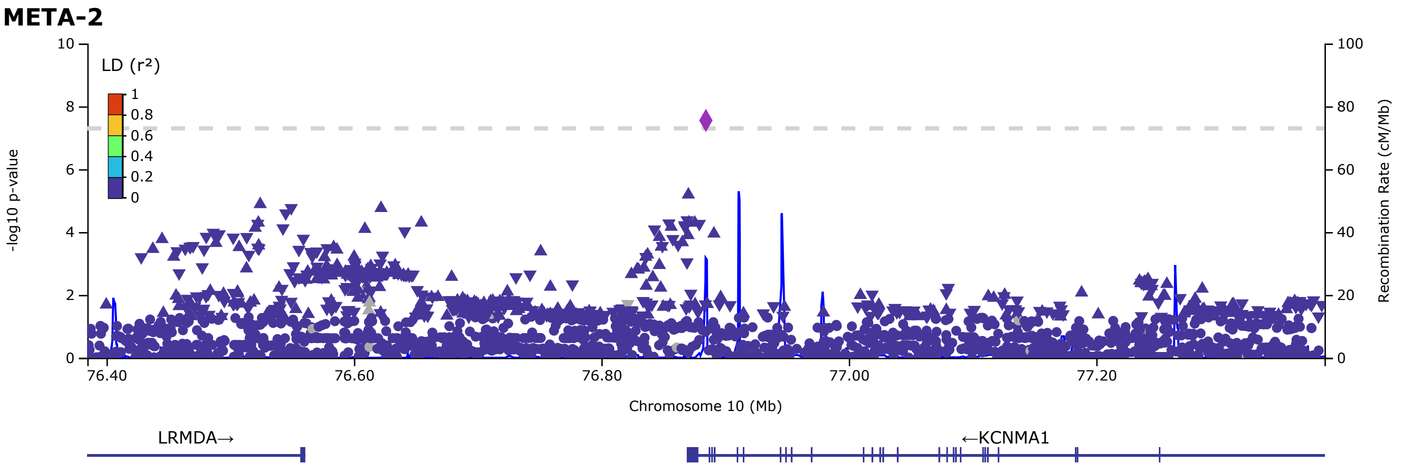

Figure S17. Regional plot of the novel UL association on chr10 near *KCNMA1* [META-2].

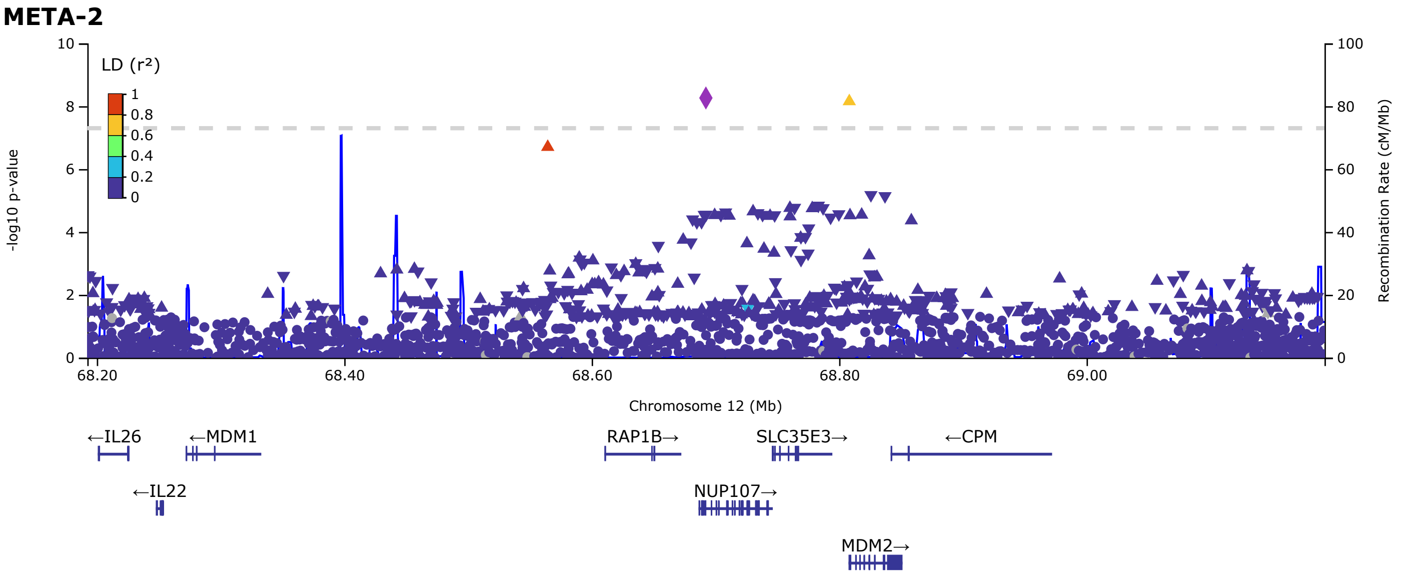

Figure S18. Regional plot of the novel UL association on chr12 near *NUP107* [META-2].

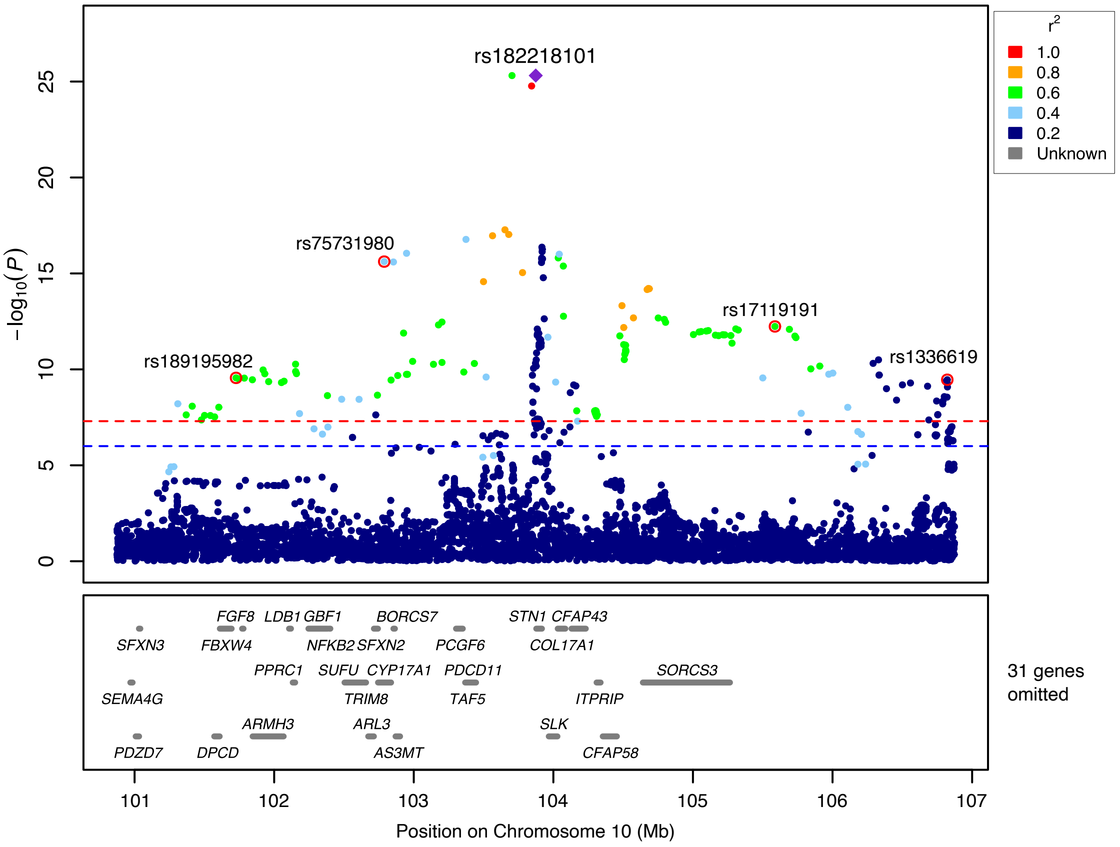

Figure S19. Regional plot of the association signal at 10q24.32 spanning to a 6MB region [META-2].

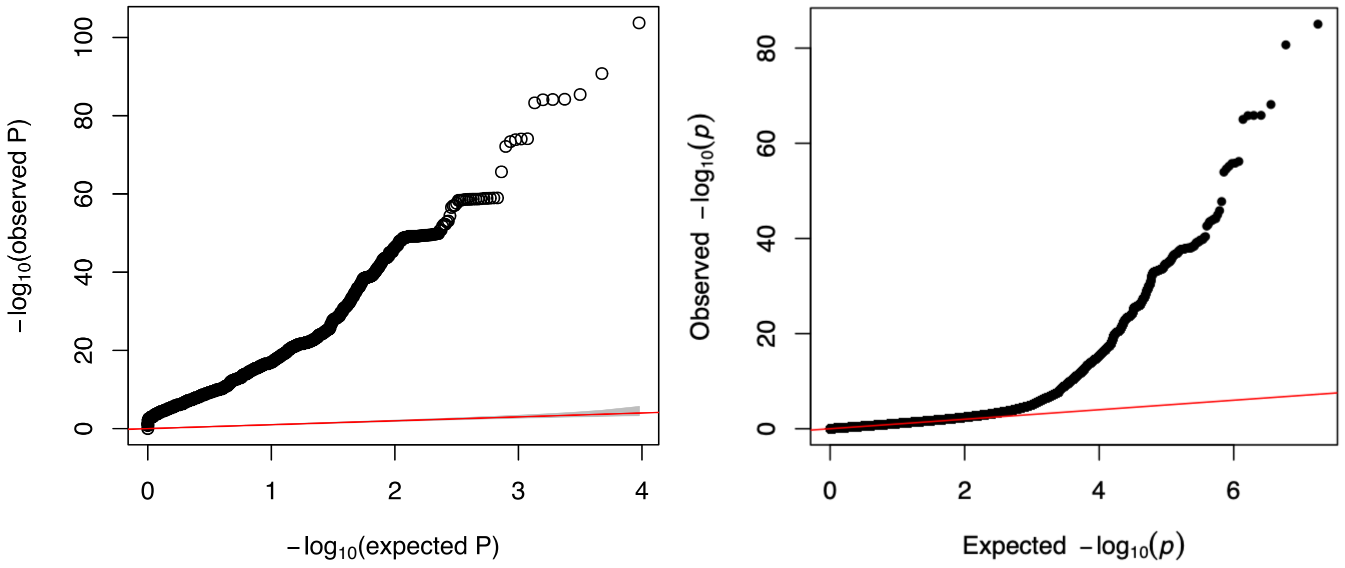

Figure S20. Quantile-quantile plots of the P-values.
We conducted two sets of meta-analyses with data from FinnGen and a previous UL GWAS[1]. META-1 was limited to the top 10,000 variants from the previous study resulting in an atypical distribution of p-values compared with standard genome-wide analyses (left). META-2 was conducted genome-widely (right). Genomic inflation factor obtained using LDSC software[2] and genome-wide results from META-2 was 1.105 suggesting minor inflation in the test statistics that was mostly accounted by a polygenic signal with the intercept being close to one (1.0066)[2].

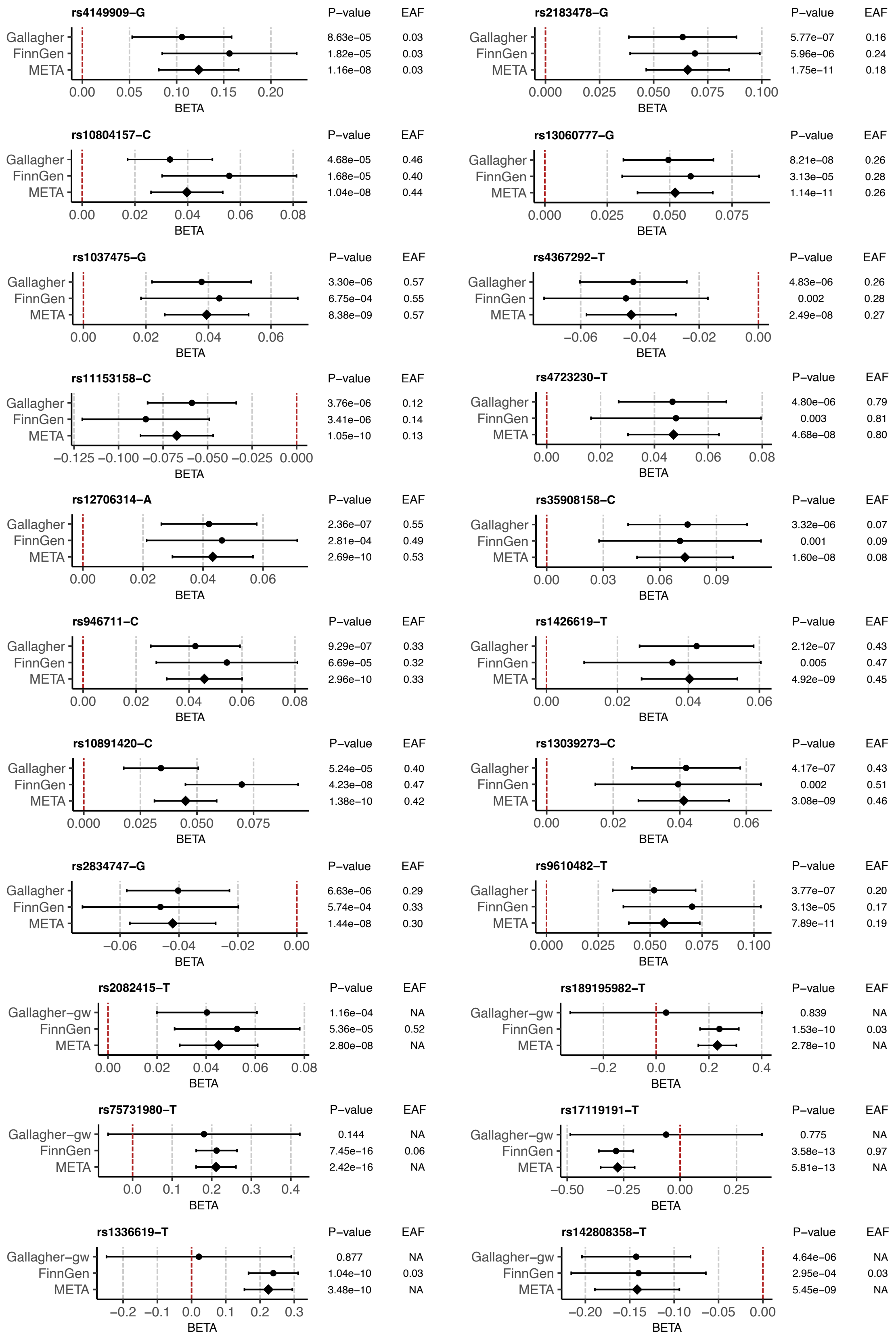

Figure S21. Forest plot of the effect estimates of the association lead variants in the novel loci.
The effect estimates and p-values are extracted from the original genetic association results (FinnGen, Gallagher top 10,000 [Gallagher], Gallagher genome-wide [Gallagher-gw]) and the corresponding meta-analyses. The horizontal lines represent the 95% confidence intervals of the effect estimates.
FinnGen, n=123,579; Gallagher, n=302,979; Gallagher genome-wide (gw), n=244,324; META (with Gallagher, i.e. META-1), n=426,558; META (with Gallagher-gw, i.e. META-2), n=367,903.

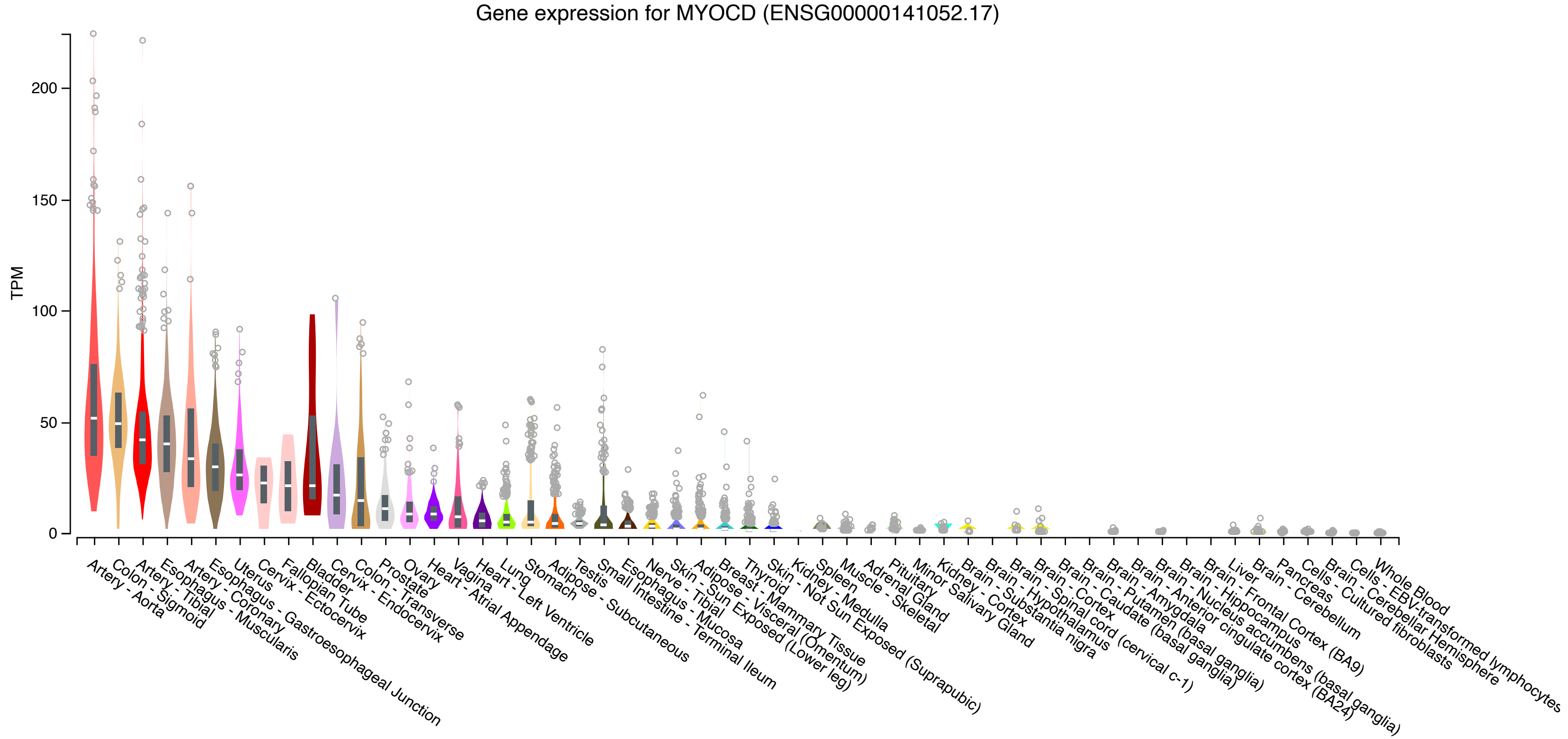

Figure S22. ***MYOCD*** **expression in tissues.**
The data was extracted from the GTEx Portal[3] on 02/10/2021. Expression values are shown in Transcripts Per Million (TPM); box plots are shown as median and 25th and 75th percentiles; points are displayed as outliers if they are above or below 1.5 times the interquartile range.
Artery – Aorta, n=432; Colon – Sigmoid, n=373; Artery – Tibial, n=663; Esophagus – Muscularis, n=515; Artery – Coronary, n=240; Esophagus – Gastroesophageal Junction, n=375; Uterus, n=142; Cervix – Ectocervix, n=9; Fallopian Tube, n=9; Bladder, n=21; Cervix – Endocervix, n=10; Colon – Transverse, n=406; Prostate, n=245; Ovary, n=180; Heart – Atrial Appendage, n=429; Vagina, n=156; Heart – Left Ventricle, n=423; Lung, n=578; Stomach, n=359; Adipose – Subcutaneous, n=663; Testis, n=361; Small Intestine – Terminal Ileum, n=187; Esophagus – Mucosa, n=555; Nerve – Tibial, n=619; Skin – Sun Exposed (lower leg), n=701; Adipose – Visceral (Omentum), n=541; Breast – Mammary Tissue, n=459; Thyroid, n=653; Skin – Not Sun Exposed (Suprapubic), n=604; Kidney – Medulla, n=4; Spleen, n=241; Muscle – Skeletal, n=803; Adrenal Gland, n=258; Pituitary, n=283; Minor Salivary Gland, n=162; Kidney – Cortex, n=85; Brain – Substantia nigra, n=139; Brain – Hypothalamus, n=202; Brain – Spinal cord (cervical c-1), n=159; Brain – Cortex, n=255; Brain – Caudate (basal ganglia), n=246; Brain – Putamen (basal ganglia), n=205; Brain – Amygdala, n=152; Brain – Anterior cingulate cortex (BA24), n=176; Brain – Nucleus accumbens (basal ganglia), n=246; Brain – Hippocampus, n=197; Brain – Frontal Cortex (BA9), n=209; Liver, n=226; Brain – Cerebellum, n=241; Pancreas, n=328; Cells – Cultured fibroblasts, n=504; Brain – Cerebellar Hemisphere, n=215; Cells – EBV-transformed lymphocytes, n=174; Whole Blood, n=755.

Tissue	Samples	NES	p-value	m-value
Kidney - Cortex	73	0.184	0.1	0.00
Artery - Coronary	213	0.174	3.7e-3	0.00
Artery - Aorta	387	0.101	0.01	0.00
Adrenal Gland	233	0.0778	0.2	0.00
Breast - Mammary Tissue	396	0.0751	0.03	0.00
Brain - Anterior cingulate cortex (BA24)	147	0.0730	0.1	0.00
Brain - Spinal cord (cervical c-1)	126	0.0681	0.4	0.00
Spleen	227	0.0600	0.3	0.00
Adipose - Visceral (Omentum)	469	0.0595	0.1	0.00
Brain - Hypothalamus	170	0.0519	0.3	0.00
Brain - Substantia nigra	114	0.0423	0.5	0.00
Muscle - Skeletal	706	0.0376	0.09	0.00
Nerve - Tibial	532	0.0364	0.3	0.00
Brain - Hippocampus	165	0.0304	0.6	0.00
Artery - Tibial	584	0.0277	0.3	0.00
Liver	208	0.0239	0.6	0.00
Minor Salivary Gland	144	0.0231	0.8	0.00900
Adipose - Subcutaneous	581	0.0228	0.5	0.00
Whole Blood	670	0.0227	0.2	0.00
Heart - Left Ventricle	386	0.0203	0.6	0.00
Brain - Cortex	205	0.0151	0.7	0.00
Testis	322	0.0120	0.6	0.00
Stomach	324	0.00944	0.8	0.00
Brain - Cerebellum	209	0.00199	1	0.00300
Brain - Caudate (basal ganglia)	194	0.000202	1	0.00
Brain - Frontal Cortex (BA9)	175	0.000182	1	0.00
Skin - Not Sun Exposed (Suprapubic)	517	-0.00388	0.9	0.00
Skin - Sun Exposed (Lower leg)	605	-0.0108	0.7	0.00
Brain - Putamen (basal ganglia)	170	-0.0131	0.7	0.00
Vagina	141	-0.0153	0.8	0.00
Pituitary	237	-0.0259	0.6	0.00
Colon - Sigmoid	318	-0.0261	0.3	0.00
Colon - Transverse	368	-0.0334	0.1	0.00400
Cells - Cultured fibroblasts	483	-0.0405	0.2	0.00700
Brain - Amygdala	129	-0.0451	0.5	0.0120
Small Intestine - Terminal Ileum	174	-0.0463	0.2	0.0260
Esophagus - Mucosa	497	-0.0490	0.1	0.0250
Prostate	221	-0.0507	0.2	0.0240
Brain - Nucleus accumbens (basal ganglia)	202	-0.0598	0.1	0.0390
Lung	515	-0.0600	0.05	0.0470
Esophagus - Muscularis	465	-0.0660	6.9e-4	0.0830
Thyroid	574	-0.0678	0.02	0.0350
Esophagus - Gastroesophageal Junction	330	-0.0776	1.5e-3	0.0960
Brain - Cerebellar Hemisphere	175	-0.0873	0.1	0.0450
Cells - EBV-transformed lymphocytes	147	-0.132	0.2	0.258
Heart - Atrial Appendage	372	-0.165	3.1e-7	1.00
Ovary	167	-0.226	8.7e-6	1.00
Uterus	129	-0.242	2.2e-3	0.908
Pancreas	305	-0.586	2.8e-16	1.00

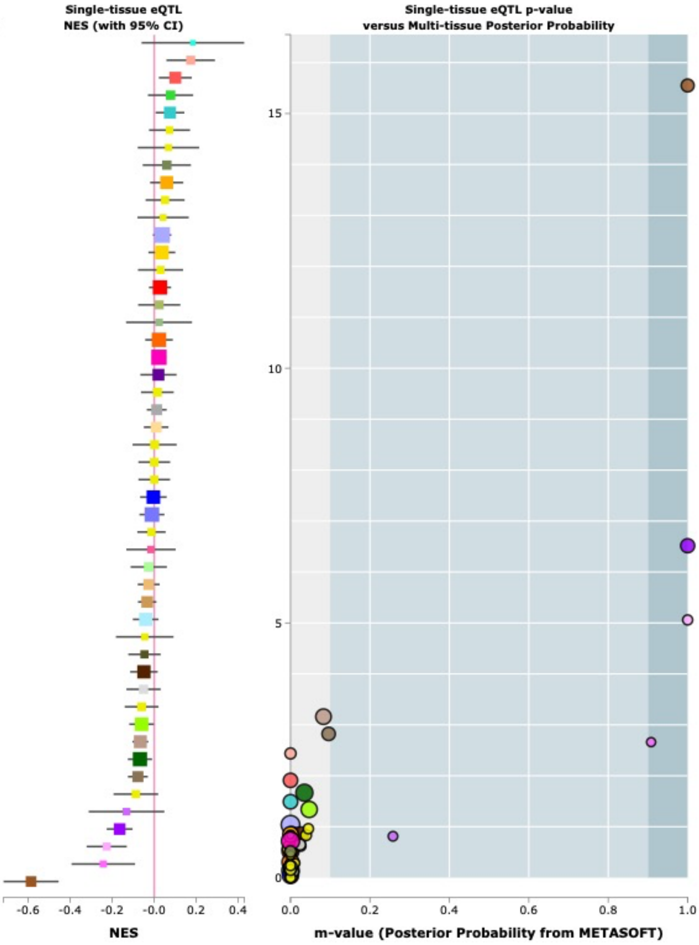

Figure S23. The association of rs13039273-C with *RBM38* expression.
'Samples' indicate the number of RNA-seq samples with genotype. Normalized effect size (NES) is the slope of the linear regression of the normalized data versus the three genotype categories using single-tissue expression quantitative trait (eQTL) analysis, representing eQTL effect size; the error bars represent the corresponding 95% confidence intervals. 'P-value' originates from a t-test that compares observed NES from single-tissue eQTL analysis to a null NES of 0 (p=1.38x10^{-19} for the meta-analyzed effects). 'M-value' indicates the posterior probability that an eQTL effect exists in each tissue tested in the cross-tissue meta-analysis. NES are given for the C allele that was the effect allele in the present study. The eQTL screening was considered exploratory and no multiple testing correction was applied.

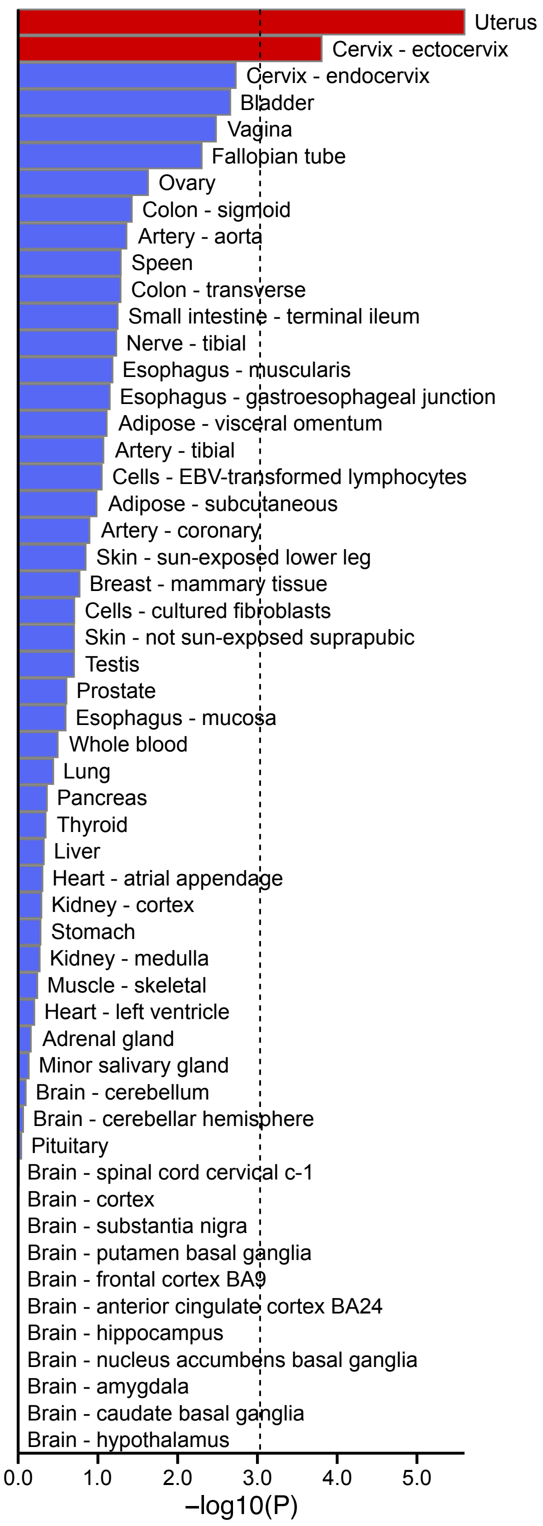

Figure S24. Results of MAGMA tissue expression analysis.
MAGMA[4] tissue expression analysis tests for a positive relationship between tissue-specific gene expression profiles and disease-gene associations. Tissue-specific gene expression data were from GTEx v8[3]. The analysis was conducted using FUMA[5].

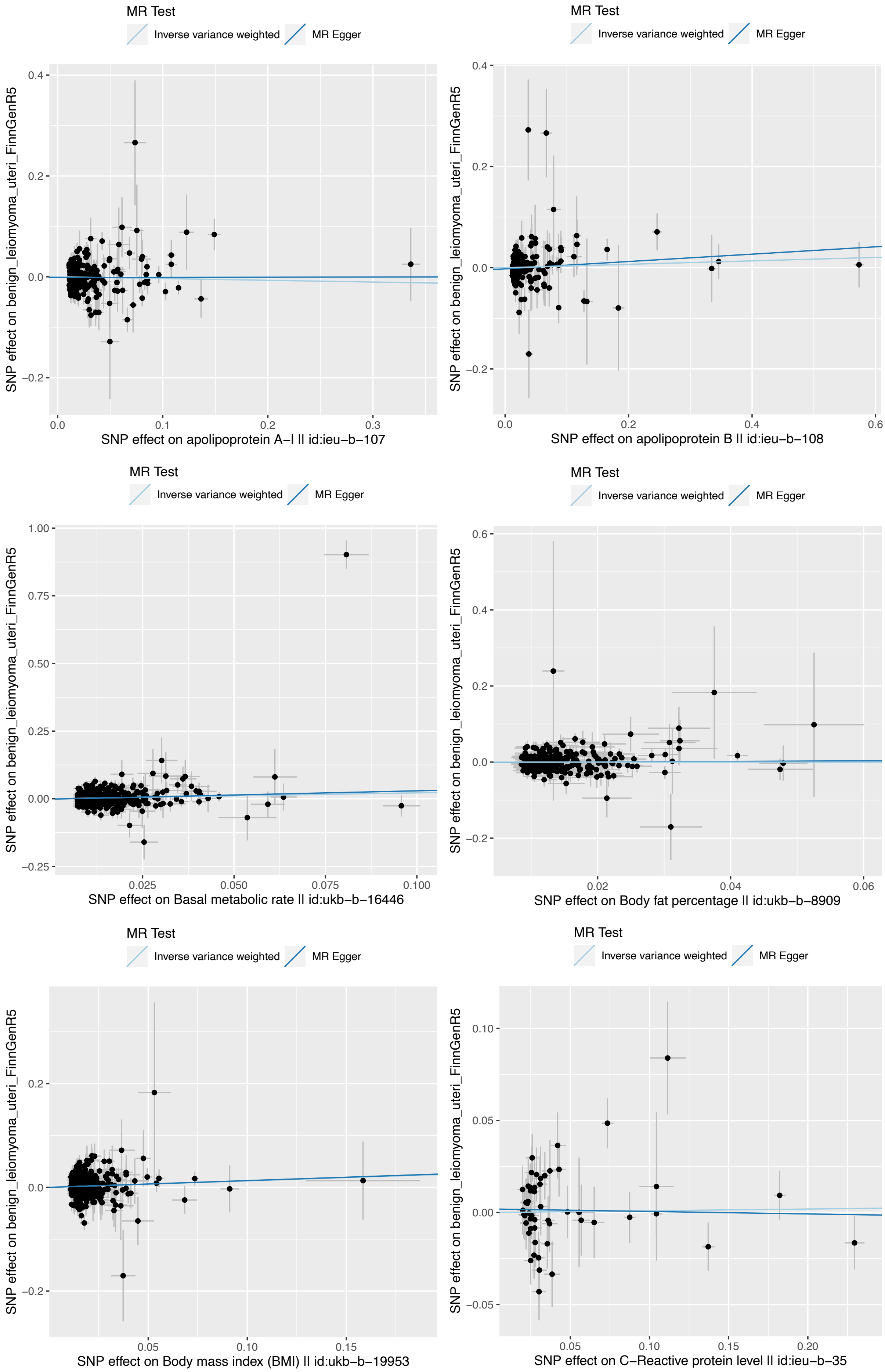

(Figure S25. Figure legend on page 26.)

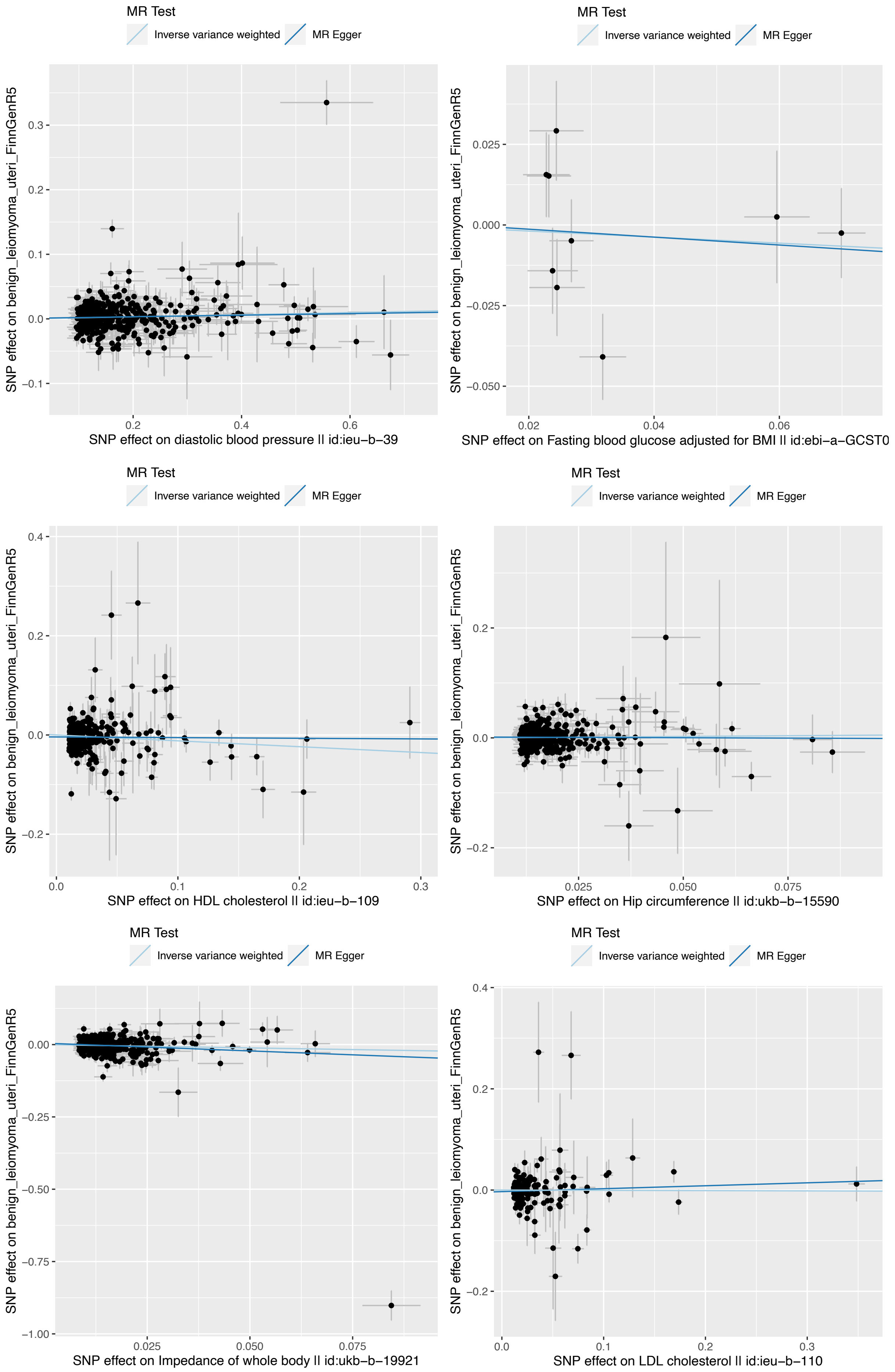

(Figure S25. Figure legend on page 26.)

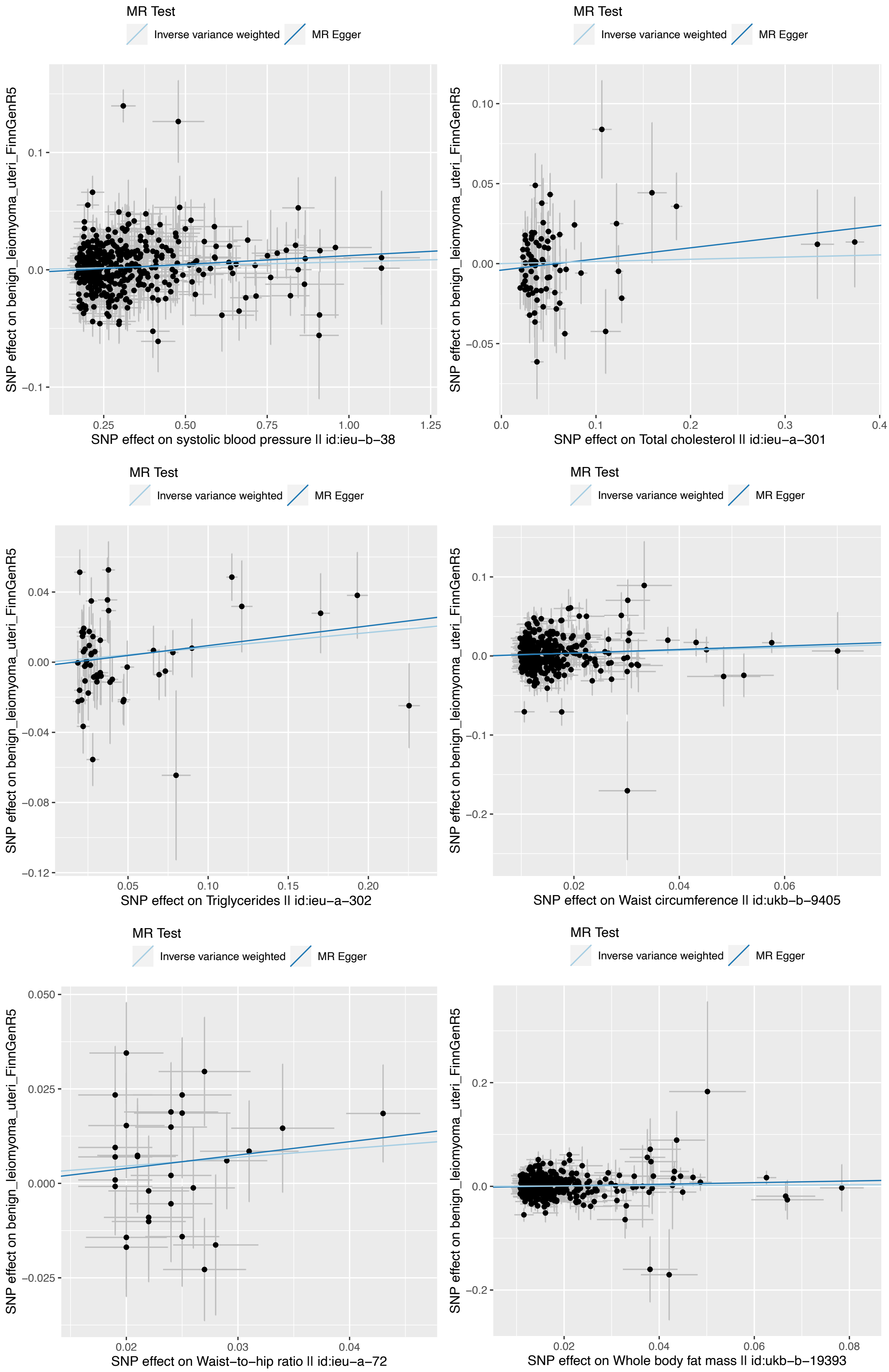

(Figure S25. Figure legend on page 26.)

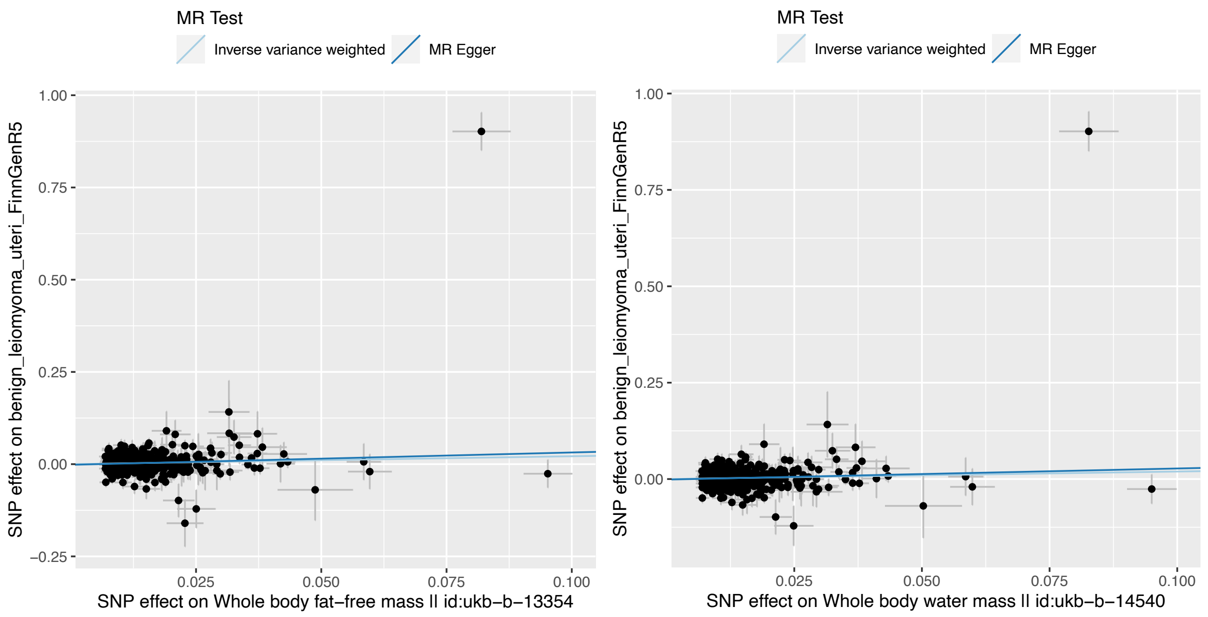

Figure S25. MR scatter plots: UL as outcome, other traits as exposure.
Genetic instruments for UL were extracted from the GWAS results obtained from FinnGen (n=123,579), and for other traits from the GWAS database provided by the MRC IEU as integrated in the TwoSampleMR R library (sample sizes are given below). LD pruning was completed using European population reference, threshold of r2=0.001, and clumping window of 10 kb. The error bars represent the 95% confidence intervals of the SNP effects.
Apolipoprotein A-I (ieu-b-107), n=393,193; Apolipoprotein B (ieu-b-108), n=439,214; Basal metabolic rate (ukb-b-16446), n=454,874; Body fat percentage (ukb-b-8909), n=454,633; Body mass index (BMI) (ukb-b-19953), n=461,460; C-reactive protein (bbj-a-14), n=75,391; Diastolic blood pressure (ieu-b-39), n=757,601; Fasting blood glucose adjusted for BMI (ebi-a-GCST007858), n=33,231, HDL cholesterol (ieu-b-1099, n=403,943; Hip circumference (ukb-b-15590), n=462,117; Impedance of whole body (ukb-b-19921), n=454,840; LDL cholesterol (ieu-b-110), n=440,546, Systolic blood pressure (ieu-b-38), n=757,601; Total cholesterol (ieu-a-301), n=187,365; Triglycerides (ieu-b-111), n=441,016; Waist circumference (ukb-b-9405), n=462,166; Waist-to-hip ratio (ieu-a-72), n=224,459; Whole body fat mass (ukb-b-19393), n=454,137; Whole body fat-free mass (ukb-b-13354), n=454,850, Whole body water mass (ukb-b-14540), n=454,888.

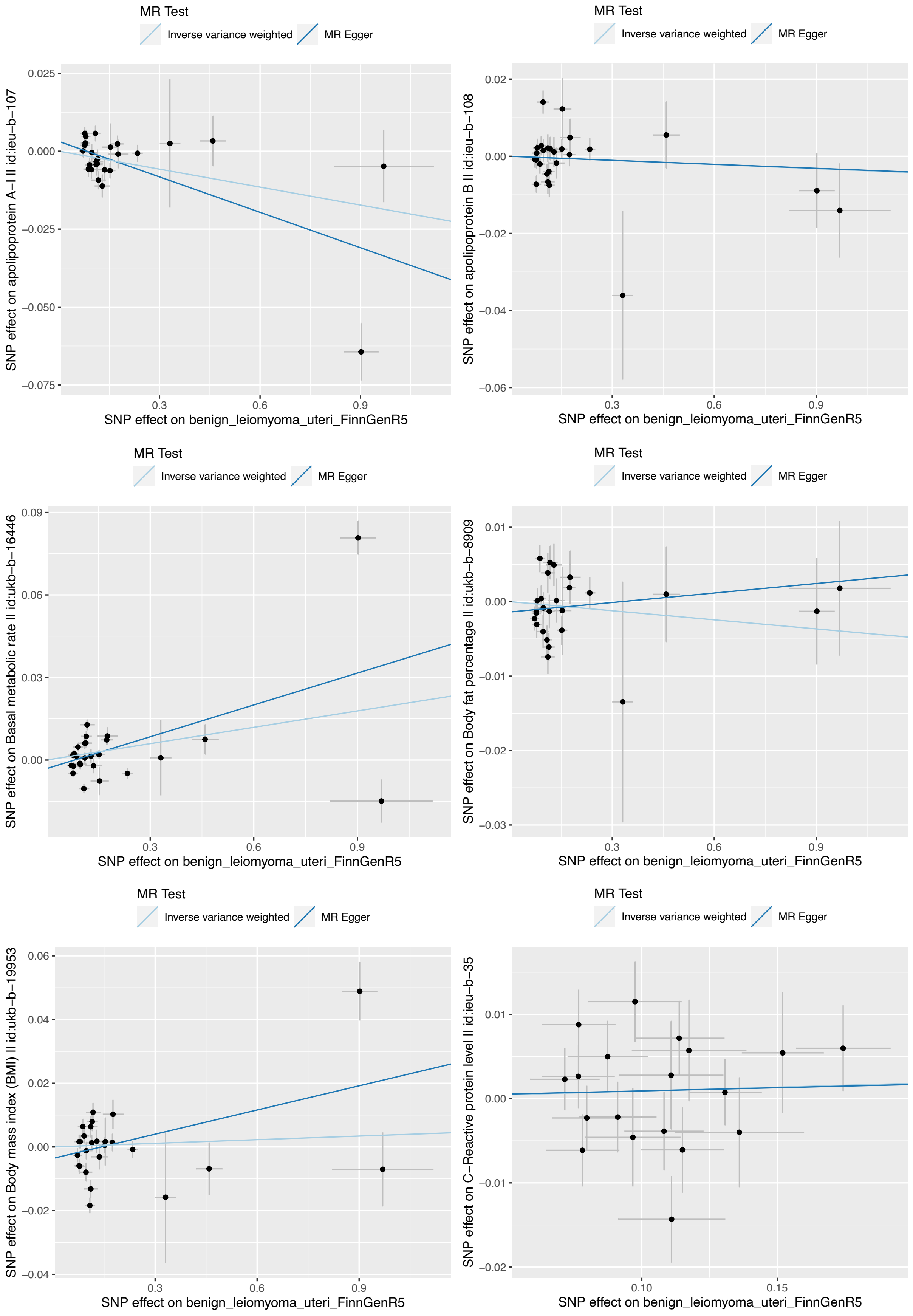

(Figure S26. Figure legend on page 30.)

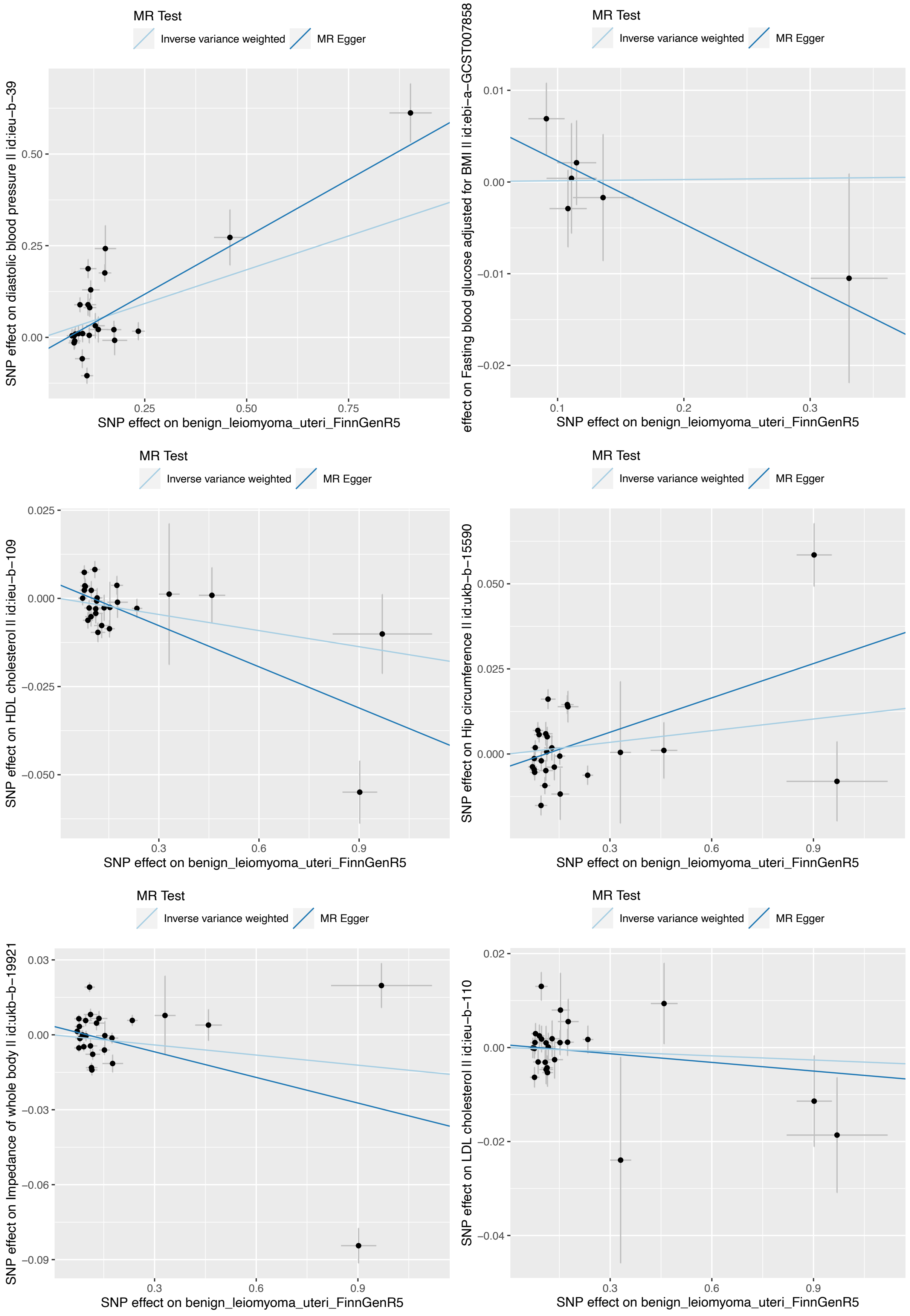

(Figure S26. Figure legend on page 30.)

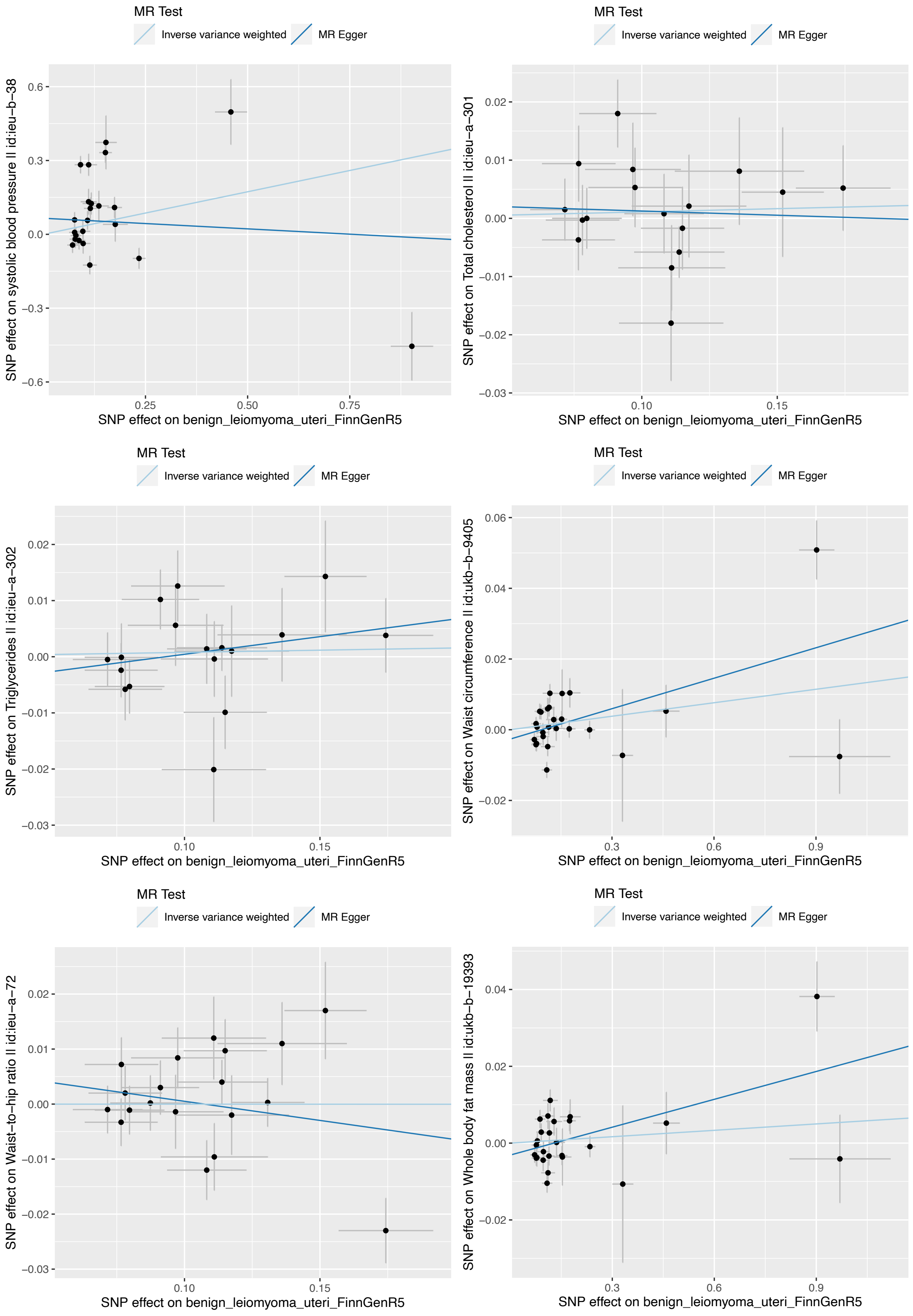

(Figure S26. Figure legend on page 30.)

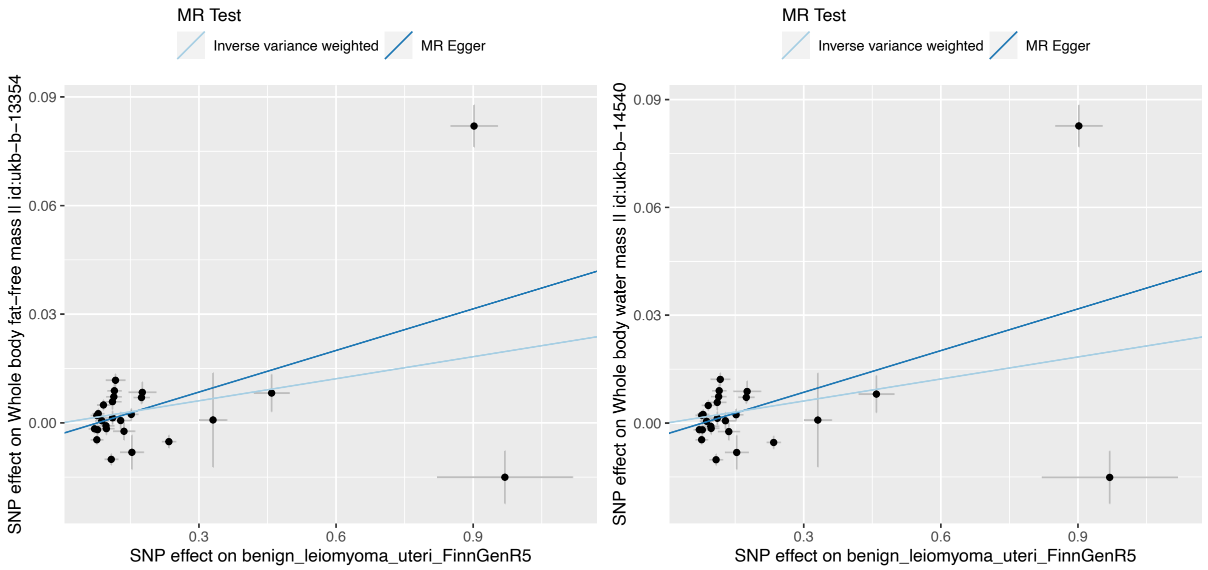

Figure S26. MR scatter plots: UL as exposure, other traits as outcome.
Genetic instruments for UL were extracted from the GWAS results obtained from FinnGen (n=123,579), and for other traits from the GWAS database provided by the MRC IEU as integrated in the TwoSampleMR R library (sample sizes are given below). LD pruning was completed using European population reference, threshold of r2=0.001, and clumping window of 10 kb. The error bars represent the 95% confidence intervals of the SNP effects.
Apolipoprotein A-I (ieu-b-107), n=393,193; Apolipoprotein B (ieu-b-108), n=439,214; Basal metabolic rate (ukb-b-16446), n=454,874; Body fat percentage (ukb-b-8909), n=454,633; Body mass index (BMI) (ukb-b-19953), n=461,460; C-reactive protein (bbj-a-14), n=75,391; Diastolic blood pressure (ieu-b-39), n=757,601; Fasting blood glucose adjusted for BMI (ebi-a-GCST007858), n=33,231, HDL cholesterol (ieu-b-1099, n=403,943; Hip circumference (ukb-b-15590), n=462,117; Impedance of whole body (ukb-b-19921), n=454,840; LDL cholesterol (ieu-b-110), n=440,546, Systolic blood pressure (ieu-b-38), n=757,601; Total cholesterol (ieu-a-301), n=187,365; Triglycerides (ieu-b-111), n=441,016; Waist circumference (ukb-b-9405), n=462,166; Waist-to-hip ratio (ieu-a-72), n=224,459; Whole body fat mass (ukb-b-19393), n=454,137; Whole body fat-free mass (ukb-b-13354), n=454,850, Whole body water mass (ukb-b-14540), n=454,888.

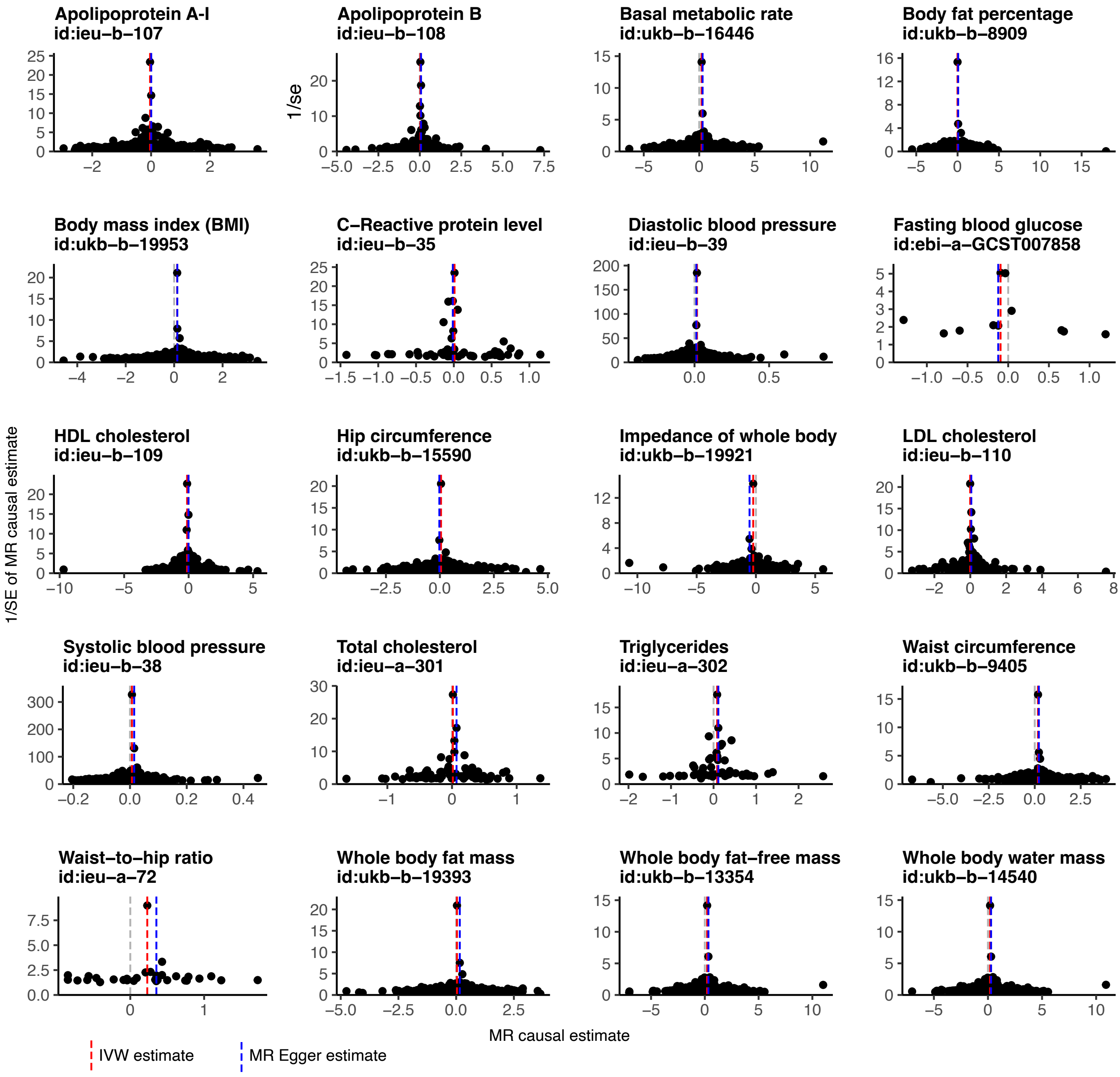

Figure S27. Funnel plots of MR causal estimates vs. their precision.
Each data point corresponds to an individual genetic variant. The x-axis corresponds to the coefficient of the variant's UL association divided by the coefficient of the variant's exposure association, *i.e.*, Wald ratio. Genetic instruments for UL were extracted from the GWAS results obtained from FinnGen, and for other traits from the GWAS database provided by the MRC IEU as integrated in the TwoSampleMR R library. LD pruning was completed using European population reference, threshold of r^2=0.001, and clumping window of 10 kb.

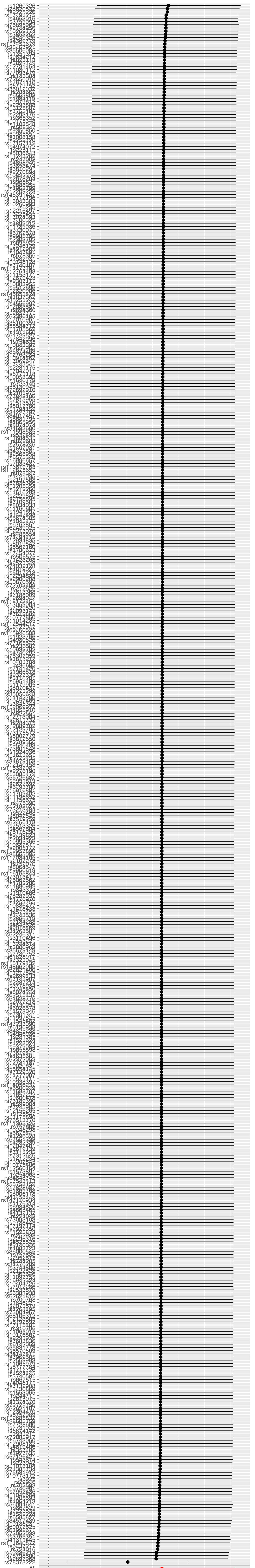

Figure S28. Leave-one-out: exposure basal metabolic rate, outcome UL.

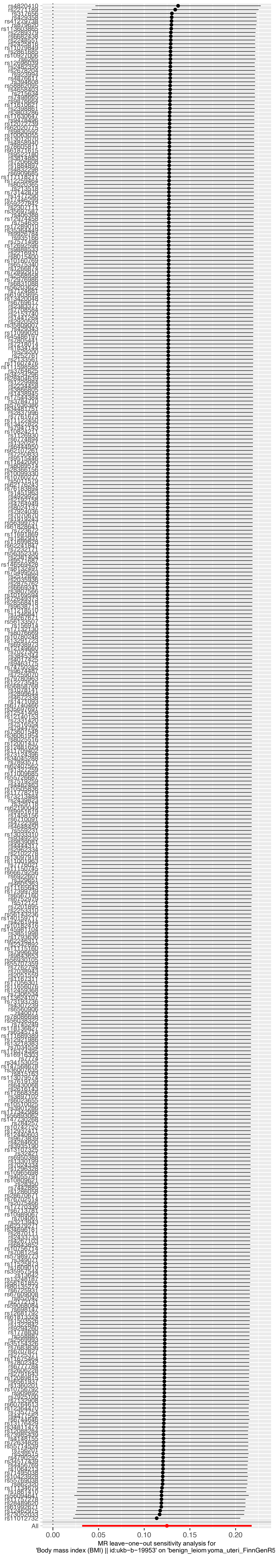

Figure S29. Leave-one-out: exposure body mass index (BMI), outcome UL.

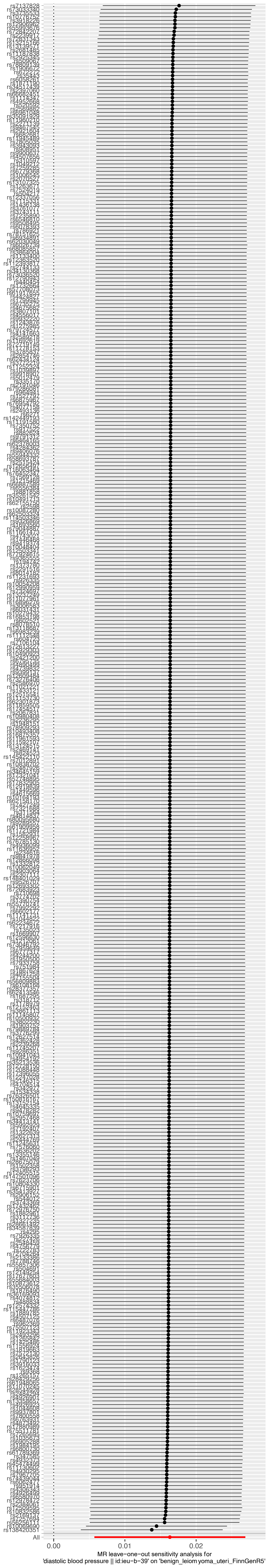

Figure S30. Leave-one-out: exposure diastolic blood pressure, outcome UL.

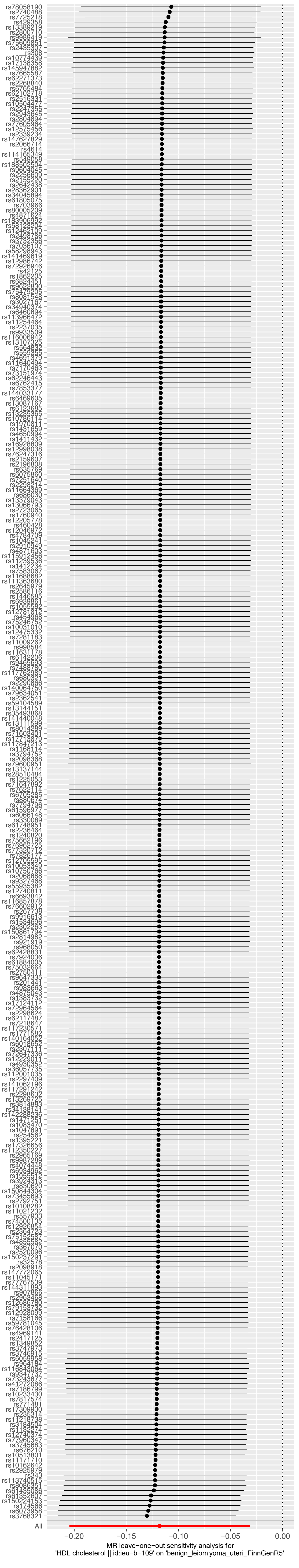

Figure S31: Leave-one-out: exposure HDL cholesterol, outcome UL.

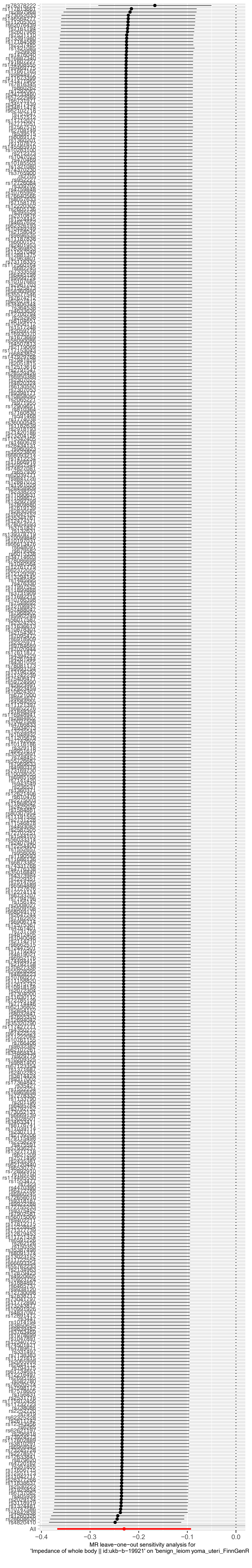

Figure S32: Leave-one-out: exposure impedance of whole body, outcome UL.

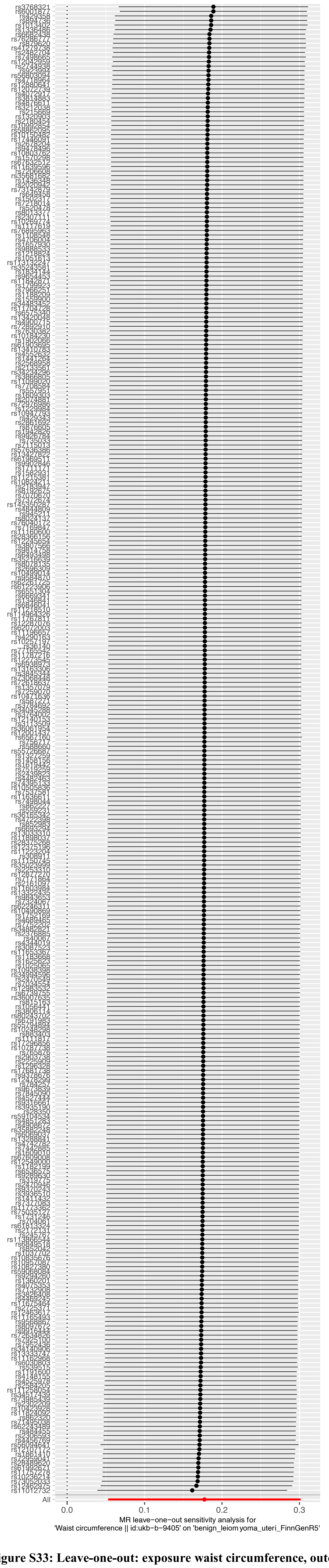

Figure S33: Leave-one-out: exposure waist circumference, outcome UL.

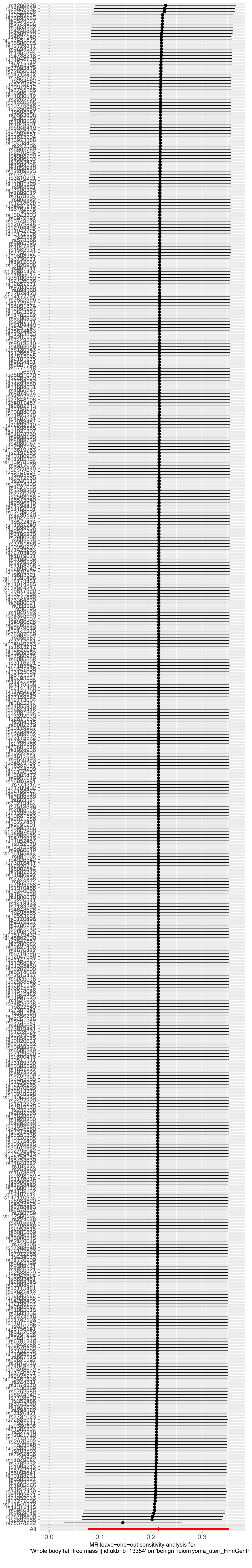

Figure S34: Leave-one-out: exposure whole body fat-free mass, outcome UL.

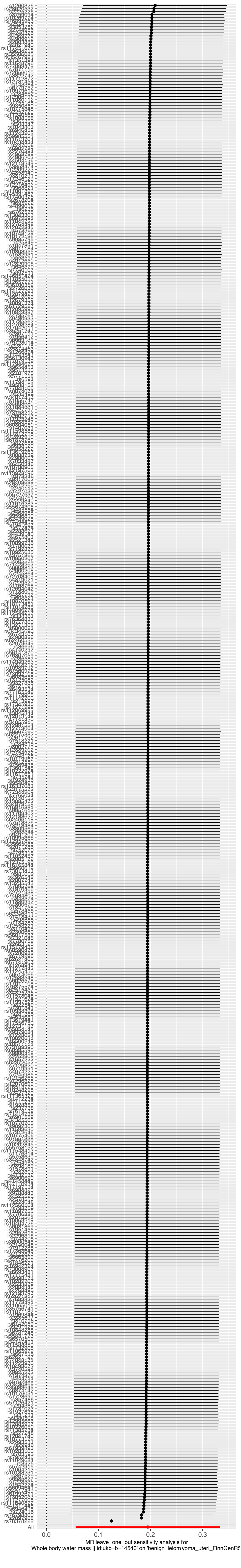

Figure S35: Leave-one-out: exposure whole body water mass, outcome UL.

Table S1. Genome-wide significant (p<5x10^{-8}) loci in META-1.

The table reports distinct loci (more than 1Mb apart) containing at least one variant associated with uterine leiomyomata (UL) at p<5x10^{-8}, *i.e.*, the standard threshold for genome-wide significance. META-1 was conducted in 53,534 UL cases and 373,024 female controls from FinnGen and a previous UL meta-analysis[1] using fixed-effect inverse variance-weighted method implemented in METAL. Due to data usage policies, META-1 was limited to the top 10,000 variants from the previous study.

Chr:Pos (hg38)	Nearest gene	Candidate gene	rsID	EA	NEA	EAF	OR (95% CI)	P-value	HetPVal
1:22141722	*WNT4*	*WNT4*	rs3820282	T	C	0.154	1.15 (1.13-1.17)	2.60E-49	0.016
1:172162145	*DNM3*	*DNM3*	rs61807787	T	C	0.290	1.04 (1.03-1.06)	3.89E-08	0.729
1:241860596	*EXO1*	*EXO1,FH*	rs4149909	G	A	0.033	1.13 (1.08-1.18)	1.16E-08	0.265
1:244151650	*ZBTB18,C1orf100*	*ZBTB18*	rs2183478	G	A	0.182	1.07 (1.05-1.09)	1.75E-11	0.774
1:248897507	*PGBD2*	*ZNF692*	rs4335411	A	G	0.760	1.06 (1.04-1.08)	4.12E-10	0.376
2:11540277	*GREB1*	*GREB1*	rs35417544	T	C	0.735	1.08 (1.07-1.10)	3.94E-24	0.716
2:28106534	*BABAM2*	*BABAM2*	rs74576866	G	A	0.080	1.09 (1.06-1.11)	7.74E-11	0.780
2:66863235	*MEIS1*	*MEIS1*	rs17631680	C	T	0.100	0.93 (0.91-0.95)	1.93E-10	0.127
2:99454113	*REV1*	*REV1*	rs13392042	G	A	0.577	1.05 (1.03-1.06)	4.21E-11	0.217
2:207258660	*MYOSLID,KLF7*	*MYOSLID*	rs10804157	C	T	0.443	1.04 (1.03-1.05)	1.04E-08	0.143
2:241720139	*ING5*	*ING5*	rs34766121	T	C	0.237	1.06 (1.04-1.08)	5.02E-10	0.278
3:4674530	*ITPR1*	*ITPR1*	rs3804984	C	T	0.384	0.95 (0.93-0.96)	8.06E-15	0.021
3:24213259	*THRB*	*THRB*	rs1010961	A	T	0.447	1.04 (1.03-1.06)	1.64E-10	0.109
3:27488262	*SLC4A7*	*NEK10*	rs35701251	A	T	0.243	1.05 (1.03-1.06)	1.59E-08	0.909
3:169768720	*ACTRT3,TERC*	*TERC*	rs35446936	A	G	0.252	0.94 (0.92-0.95)	1.01E-16	0.033
3:185807411	*IGF2BP2*	*IGF2BP2*	rs13060777	G	A	0.264	1.05 (1.04-1.07)	1.14E-11	0.595
4:53684007	*LNX1*	*LNX1*	rs62323682	C	T	0.064	1.16 (1.13-1.19)	5.67E-25	0.498
4:69735020	*SULT1B1*	*SULT1E1*	rs12640488	G	A	0.475	1.07 (1.05-1.08)	6.21E-21	0.511
4:94572095	*PDLIM5*	*BMPR1B*	rs2452597	G	A	0.306	1.05 (1.03-1.06)	9.42E-11	0.781
4:99031559	*METAP1,ADH5*	*ADH5*	rs1037475	G	A	0.566	1.04 (1.03-1.05)	8.38E-09	0.707
5:1279913	*TERT*	*TERT*	rs2242652	A	G	0.205	1.12 (1.11-1.14)	5.86E-41	0.005
5:133099880	*HSPA4*	*HSPA4*	rs4367292	T	C	0.266	0.96 (0.94-0.97)	2.49E-08	0.882
5:177023836	*ZNF346*	*UIMC1,FGFR4*	rs2456181	G	C	0.494	1.05 (1.04-1.07)	4.20E-12	0.059
6:34240996	*HMGA1*	*HMGA1*	rs41269026	A	C	0.041	1.11 (1.07-1.15)	4.02E-09	0.998
6:36653670	*CDKN1A*	*CDKN1A*	rs10456443	A	G	0.203	0.95 (0.93-0.96)	2.67E-10	0.039
6:109054915	*SESN1*	*SESN1*	rs11153158	C	T	0.126	0.93 (0.92-0.95)	1.05E-10	0.243
6:152241136	*SYNE1*	*ESR1*	rs58415480	G	C	0.178	1.22 (1.19-1.24)	1.86E-104	0.002
7:33008785	*FKBP9,NT5C3A*	*BBS9*	rs4723230	T	C	0.797	1.05 (1.03-1.07)	4.68E-08	0.946
7:117273513	*WNT2*	*WNT2*	rs2270206	A	C	0.153	1.06 (1.04-1.08)	1.24E-09	0.525
7:121132432	*CPED1,WNT16*	*WNT16*	rs12706314	A	G	0.531	1.04 (1.03-1.06)	2.69E-10	0.777
7:130935964	*LINC-PINT*	*LINC-PINT*	rs35908158	C	T	0.077	1.08 (1.05-1.10)	1.60E-08	0.883
8:30452819	*RBPMS*	*RBPMS*	rs13275869	C	T	0.486	0.96 (0.95-0.97)	8.64E-09	0.520
8:128506035	*LINC00824*	*LINC00824*	rs1516980	C	A	0.248	0.96 (0.94-0.97)	2.72E-08	0.987
9:680714	*KANK1,ANKRD15*	*KANK1*	rs10815466	A	G	0.168	1.10 (1.08-1.12)	9.51E-24	0.060
9:89639982	*GADD45G,SEMA4D*	*GADD45G*	rs28508285	G	A	0.091	1.07 (1.04-1.09)	1.49E-08	0.362
10:21517903	*SKIDA1*	*DNAJC1*	rs946711	C	A	0.331	1.05 (1.03-1.06)	2.96E-10	0.460
10:31678920	*ZEB1,ARHGAP12*	*ZEB1*	rs72784785	C	T	0.225	0.93 (0.92-0.95)	2.35E-16	0.598
10:88331783	*RNLS*	*RNLS*	rs1426619	T	C	0.445	1.04 (1.03-1.06)	4.92E-09	0.653
10:103918139	*STN1*	*SH3PXD2A*	rs4387287	C	A	0.842	0.91 (0.89-0.92)	5.61E-25	0.235
11:197557	*ODF3,BET1L*	*PKP3*	rs7103852	G	A	0.923	1.13 (1.10-1.16)	5.10E-21	0.575
11:30204981	*FSHB*	*FSHB*	rs11031006	A	G	0.150	0.91 (0.89-0.93)	1.62E-22	0.708
11:32342641	*WT1*	*WT1*	rs2057178	A	G	0.131	1.14 (1.12-1.17)	7.32E-41	3.23E-05
11:35062086	*PDHX*	*CD44*	rs2553773	G	C	0.561	1.07 (1.06-1.09)	1.19E-23	0.074
11:108444879	*C11orf65*	*ATM*	rs149934734	T	C	0.021	1.36 (1.29-1.42)	4.74E-36	0.018
11:112703765	*LOC105369496*	*LOC105369496*	rs10891420	C	T	0.421	1.05 (1.03-1.06)	1.38E-10	0.019
12:46402739	*SLC38A2*	*SLC38A2*	rs2131371	C	A	0.685	1.08 (1.06-1.09)	1.03E-23	0.462
12:70756878	*PTPRR*	*PTPRR*	rs11178393	C	T	0.102	0.92 (0.90-0.95)	7.46E-12	0.402
12:123379073	*KMT5A,PITPNM2*	*KMT5A*	rs28583837	A	G	0.207	0.95 (0.93-0.96)	9.67E-10	0.379
13:40149807	*FOXO1*	*FOXO1*	rs117245733	A	G	0.021	1.42 (1.35-1.49)	1.78E-41	0.000
15:67922458	*SKOR1,PIAS1*	*PIAS1*	rs12148374	C	T	0.442	0.96 (0.95-0.97)	1.37E-09	0.832
16:50059327	*HEATR3*	*BRD7*	rs12599260	A	G	0.728	1.05 (1.04-1.07)	1.09E-11	0.002
16:51447685	*AC007344.1*	*AC007344.1*	rs66998222	A	G	0.193	0.94 (0.93-0.96)	7.53E-12	0.345
17:7668434	*TP53*	*TP53*	rs78378222	G	T	0.014	1.81 (1.71-1.92)	3.88E-86	1.50E-13
17:12652500	*MYOCD*	*MYOCD*	rs12601765	T	G	0.299	1.04 (1.03-1.06)	3.08E-08	0.400
19:22032639	*ZNF257*	*ZNF257*	rs8105767	G	A	0.298	1.05 (1.03-1.06)	2.63E-09	0.143
20:5967581	*MCM8*	*MCM8*	rs16991615	A	G	0.052	1.12 (1.09-1.15)	5.15E-13	0.081
20:57441016	*CTCFL*	*RBM38*	rs13039273	C	T	0.456	1.04 (1.03-1.06)	3.08E-09	0.874
20:63638397	*STMN3*	*SLC2A4RG*	rs75691080	T	C	0.095	0.92 (0.90-0.95)	7.79E-11	0.044
21:35072824	*RUNX1*	*RUNX1*	rs2834747	G	T	0.302	0.96 (0.94-0.97)	1.44E-08	0.711
22:36287509	*MYH9,APOL1*	*MYH9*	rs9610482	T	C	0.189	1.06 (1.04-1.08)	7.89E-11	0.354
22:40269221	*TNRC6B*	*MKL1*	rs112251865	T	C	0.223	1.09 (1.07-1.11)	1.21E-26	0.157
X:70928740	*SLC7A3,MED12*	*FOXO4*	rs5936604	C	T	0.683	0.93 (0.91-0.94)	1.00E-17	0.008
X:132178061	*FRMD7,RAP2C*	*RAP2C*	rs5930554	C	T	0.312	1.16 (1.14-1.18)	1.04E-59	0.0007

Table S2. Genome-wide significant (p<5x10^{-8}) loci in META-2.

The table reports distinct loci (more than 1Mb apart) containing at least one variant associated with uterine leiomyomata (UL) at p<5x10^{-8}, *i.e.*, the standard threshold for genome-wide significance. META-2 was conducted genome-widely in 38,466 uterine leiomyomata (UL) cases and 329,437 female controls from FinnGen and a previous UL meta-analysis[1] using fixed-effect inverse variance-weighted method implemented in METAL. Indented rows indicate the lead variants of the independent association signals that were observed after conditioning the association tests on the locus-specific lead variant on the preceding row. The loci spanning over the ±1Mb locus definition are marked with asterisk (*).

Chr:Pos (hg38)	Nearest gene	Candidate gene	rsID	EA	OA	$EAF_{FinnGen}$	OR (95% CI)	P.value	HetPVal
1:22141722	*WNT4*	*WNT4*	rs3820282	t	c	0.157	1.16 (1.13-1.18)	4.11E-41	0.02952
1:172162145	*DNM3*	*DNM3*	rs61807787	t	c	0.258	1.05 (1.03-1.07)	8.45E-09	0.2702
1:241860596	*EXO1*	*EXO1,FH*	rs4149909	a	g	0.968	0.87 (0.83-0.91)	1.12E-08	0.4515
1:244151650	*ZBTB18,C1orf100*	*ZBTB18*	rs2183478	a	g	0.764	0.93 (0.91-0.95)	7.58E-12	0.4729
1:248897507	*PGBD2*	*ZNF692*	rs4335411	a	g	0.752	1.07 (1.04-1.09)	3.56E-09	0.1961
2:11562535	*GREB1*	*GREB1*	rs10929757	a	c	0.338	0.93 (0.92-0.95)	5.53E-17	0.4803
2:11515558	*GREB1*	*GREB1*	rs13407702	t	g	0.115	1.07 (1.04-1.09)	2.19E-08	n.a.
2:28213441	*BABAM2*	*BABAM2*	rs4637064	a	g	0.707	0.95 (0.93-0.97)	2.78E-09	0.1366
2:66863235	*MEIS1*	*MEIS1*	rs17631680	t	c	0.893	1.09 (1.06-1.12)	1.46E-10	0.3255
2:99447821	*REV1*	*REV1*	rs1451246	a	c	0.445	1.05 (1.03-1.07)	5.32E-10	0.2963
2:207258660	*MYOSLID,KLF7*	*MYOSLID*	rs10804157	t	c	0.595	0.95 (0.94-0.97)	3.50E-09	0.3833
2:207848303	*PLEKHM3*	*MYOSLID*	rs7584910	a	g	0.443	0.96 (0.94-0.97)	4.75E-08	n.a.
2:241710378	*ING5*	*ING5*	rs6437284	c	g	0.727	0.94 (0.92-0.96)	2.07E-09	0.2493
3:4674530	*ITPR1*	*ITPR1*	rs3804984	t	c	0.532	1.06 (1.04-1.08)	5.01E-13	0.0314
3:24213261	*THRB*	*THRB*	rs2017200	a	t	0.444	1.05 (1.03-1.07)	5.50E-10	0.2165
3:27488262	*SLC4A7*	*NEK10*	rs35701251	a	t	0.202	1.05 (1.04-1.07)	8.45E-09	0.6348
3:169768720	*ACTRT3,TERC*	*TERC*	rs35446936	a	g	0.272	0.93 (0.91-0.94)	1.33E-16	0.1353
3:185805582	*IGF2BP2*	*IFG2BP2*	rs66513933	t	c	0.709	0.95 (0.94-0.97)	3.40E-08	0.3404
4:53634371	*LNX1*	*LNX1*	rs4864806	a	g	0.051	1.17 (1.13-1.20)	5.00E-21	0.6683
4:52992781	*SCFD2*	*LNX1*	rs11735529	c	g	0.608	0.95 (0.94-0.97)	6.61E-09	n.a.
4:53301527	*SCFD2*	*LNX1*	rs188378292	a	g	0.002	1.97 (1.55-2.51)	2.98E-08	n.a.
4:69658940	*UGT2A1*	*SULT1E1*	rs4694227	a	t	0.687	1.07 (1.06-1.09)	3.99E-17	0.1984
4:69750098	*SULT1B1*	*SULT1E1*	rs41292311	t	c	0.016	0.80 (0.75-0.85)	6.82E-12	n.a.
4:94572095	*PDLIM5*	*BMPR1B*	rs2452597	a	g	0.717	0.95 (0.94-0.97)	5.05E-09	0.8824
5:1279675	*TERT*	*TERT*	rs10069690	t	c	0.296	1.12 (1.10-1.14)	1.60E-34	0.0054
5:177023836	*ZNF346*	*UIMC1,FGFR4*	rs2456181	c	g	0.499	0.95 (0.94-0.97)	2.38E-09	0.0382
6:108976313	*ARMC2*	*SESN1*	rs12196819	a	g	0.150	0.94 (0.91-0.96)	2.62E-08	0.1554
6:152241136	*SYNE1*	*SYNE1,ESR1*	rs58415480	c	g	0.769	0.82 (0.81-0.84)	2.08E-81	0.0004
6:152551137	*SYNE1*	*SYNE1,ESR1*	rs9397523	a	g	0.542	0.94 (0.93-0.96)	1.33E-12	n.a.
6:152245912	*SYNE1*	*SYNE1,ESR1*	rs6901631	t	c	0.909	1.07 (1.04-1.10)	4.02E-08	n.a.
7:120807406	*TSPAN12*	*WNT16*	rs4730982	t	g	0.674	0.95 (0.94-0.97)	3.41E-08	0.0842
8:30444949	*RBPMS*	*RBPMS*	rs4545054	t	c	0.536	1.05 (1.03-1.06)	6.29E-09	0.9012
8:129467485	*GSDMC*	*LINC00824*	rs11786929	t	c	0.743	0.95 (0.94-0.97)	5.12E-09	0.0224
9:680714	*KANK1,ANKRD15*	*DMRT1,KANK1*	rs10815466	a	g	0.211	1.11 (1.09-1.14)	3.76E-25	0.4772
9:806912	*DMRT1*	*DMRT1,KANK1*	rs35469085	c	g	0.523	0.93 (0.91-0.94)	3.31E-20	n.a.
9:854375	*DMRT1*	*DMRT1,KANK1*	rs7856727	t	g	0.581	0.95 (0.94-0.97)	4.75E-08	n.a.
10:21695031	*MLLT10*	*DNAJC1*	rs1243192	a	c	0.302	1.06 (1.04-1.07)	2.39E-10	0.4954
10:31666087	*ZEB1,ARHGAP12*	*ZEB1*	rs7090544	a	g	0.728	1.07 (1.05-1.09)	3.64E-13	0.5477
10:76884502	*KCNMA1*	*KCNMA1*	rs2082415	t	g	0.520	1.05 (1.03-1.06)	2.80E-08	0.4600
*10:101726828	*FGF8*	*SLK*	rs189195982	t	c	0.030	1.26 (1.17-1.36)	2.78E-10	0.2851
*10:102788270	*WBP1L*	*SLK*	rs75731980	t	c	0.064	1.23 (1.17-1.30)	2.42E-16	0.8007
*10:103873824	*OBFC1*	*SLK*	rs182218101	t	c	0.972	0.66 (0.61-0.72)	4.86E-26	0.3644
10:103918139	*OBFC1*	*SLK*	rs4387287	a	c	0.137	1.10 (1.08-1.12)	2.44E-18	n.a.
10:103895645	*OBFC1*	*SLK*	rs188848367	t	c	0.019	1.31 (1.20-1.43)	3.92E-09	n.a.
*10:105587387	*SORCS3*	*SLK*	rs17119191	t	g	0.972	0.76 (0.70-0.82)	5.81E-13	0.3142
*10:106822067	*SORCS1*	*SLK*	rs1336619	t	c	0.030	1.25 (1.17-1.34)	3.48E-10	0.1272
11:197557	*ODF3,BET1L*	*PKP3*	rs7103852	a	g	0.078	0.87 (0.85-0.90)	2.02E-19	0.9974
11:224063	*SIRT3*	*PKP3*	rs12222188	a	g	0.023	0.86 (0.83-0.90)	1.99E-12	n.a.
11:30204981	*FSHB*	*FSHB*	rs11031006	a	g	0.165	0.92 (0.90-0.94)	1.32E-15	0.3740
11:32343884	*WT1*	*WT1*	rs11031731	a	g	0.074	1.16 (1.14-1.19)	1.24E-37	0.0005
11:32347198	*WT1*	*WT1*	rs2207548	a	c	0.336	1.07 (1.05-1.08)	3.46E-15	n.a.
11:32325691	*WT1*	*WT1*	rs2038318	t	g	0.707	1.06 (1.04-1.08)	2.15E-11	n.a.
11:35063906	*PDHX*	*CD44*	rs2553772	t	g	0.472	0.94 (0.92-0.95)	9.85E-18	0.0799
11:108444879	*C11orf65*	*ATM*	rs149934734	t	c	0.009	1.38 (1.30-1.46)	1.74E-27	0.0308
11:108177255	*NPAT*	*ATM*	rs1519074	a	g	0.775	1.11 (1.09-1.13)	3.75E-23	n.a.

Chr:Pos (hg38)	Nearest gene	Candidate gene	rsID	EA	OA	$EAF_{FinnGen}$	OR (95% CI)	P.value	HetPVal
11:112703765	*LOC105369496*	*LOC105369496*	rs10891420	t	c	0.531	0.95 (0.94-0.97)	3.93E-10	0.0477
12:46410392	*SLC38A2*	*SLC38A2*	rs1472178	t	c	0.340	0.94 (0.92-0.95)	7.03E-15	0.9303
12:68692314	*NUP107*	*MDM2*	rs142808358	t	c	0.028	0.87 (0.83-0.91)	5.45E-09	0.9583
12:70756878	*PTPRR*	*PTPRR*	rs11178393	t	c	0.916	1.08 (1.05-1.11)	2.82E-09	0.3833
12:123405729	*KMT5A*	*KMT5A*	rs28576953	t	c	0.211	0.94 (0.92-0.96)	9.46E-10	0.1098
*13:38939164	*STOML3*	*FOXO1*	rs149775438	a	c	0.980	0.76 (0.70-0.83)	1.10E-09	0.8152
13:39726408	*COG6*	*FOXO1*	rs9548898	a	g	0.537	1.05 (1.04-1.07)	1.05E-11	n.a.
*13:40149807	*FOXO1*	*FOXO1*	rs117245733	a	g	0.030	1.46 (1.38-1.54)	9.16E-39	0.0013
13:40605661	*FOXO1*	*FOXO1*	rs7986407	a	g	0.642	0.93 (0.91-0.95)	1.79E-18	n.a.
13:39726408	*COG6*	*FOXO1*	rs9548898	a	g	0.537	1.05 (1.04-1.07)	1.45E-11	n.a.
13:40162152	*FOXO1*	*FOXO1*	rs6563799	t	c	0.245	1.06 (1.04-1.08)	1.04E-09	n.a.
13:39898446	*COG6*	*FOXO1*	rs9576914	t	c	0.554	0.95 (0.94-0.97)	2.84E-09	n.a.
15:68335825	*ITGA11*	*PIAS1*	rs2306022	t	c	0.088	1.09 (1.06-1.12)	1.68E-09	0.4266
16:50056732	*HEATR3*	*HEATR3,BRD7*	rs62033024	c	g	0.181	0.93 (0.92-0.95)	1.81E-12	0.0009
16:50048520	*CNEP1R1*	*HEATR3,BRD7*	rs2058814	t	g	0.198	0.94 (0.92-0.96)	2.05E-10	n.a.
16:51447685	*AC007344.1*	*AC007344.1*	rs66998222	a	g	0.153	0.93 (0.91-0.95)	2.12E-12	0.824
*17:7668434	*TP53*	*TP53*	rs78378222	t	g	0.983	0.51 (0.47-0.54)	9.36E-86	4.19E-09
*17:8767403	*SPDYE4*	*TP53*	rs74876186	a	g	0.013	1.40 (1.26-1.56)	1.10E-09	0.5213
17:7796745	*DNAH2*	*TP53*	rs138420351	t	c	0.039	1.38 (1.31-1.46)	1.15E-30	n.a.
17:12656880	*MYOCD*	*MYOCD*	rs12449987	t	c	0.560	0.96 (0.94-0.97)	2.05E-08	0.1577
19:22032639	*ZNF257*	*ZNF257*	rs8105767	a	g	0.682	0.95 (0.94-0.97)	9.89E-09	0.2546
20:5967581	*MCM8*	*MCM8*	rs16991615	a	g	0.021	1.14 (1.09-1.18)	9.96E-12	0.1533
20:63638397	*STMN3*	*SLC2A4RG*	rs75691080	t	c	0.114	0.93 (0.90-0.95)	2.38E-08	0.0181
22:28779069	*CCDC117*	*?? Sakaue*	rs71758825	t	tctc	0.096	1.13 (1.08-1.18)	1.07E-08	0.5253
22:36287509	*MYH9,APOL1*	*MYH9*	rs9610482	t	c	0.172	1.06 (1.04-1.08)	1.37E-09	0.5066
22:40269221	*TNRC6B*	*MKL1*	rs112251865	t	c	0.252	1.10 (1.08-1.12)	1.20E-23	0.3355
X:70762863	*TEX11*	*FOXO4*	rs5936980	a	g	0.424	1.09 (1.07-1.11)	1.52E-15	0.0201
X:132178061	*FRMD7,RAP2C*	*RAPC2*	rs5930554	t	c	0.684	0.87 (0.85-0.89)	8.36E-39	7.58E-06

n.a. = not available

Table S3. Fine-mapping results of META-1.

We applied the FinnGen finemapping pipeline (available at https://github.com/FINNGEN/finemapping-pipeline, accessed on 2/2/2022) with default parameters. In brief, the pipeline calculates linkage disequilibrium within the regions of interest with LDstore2[6] using FinnGen samples, generates 99 % credible sets using the SUm of Single Effects (SuSiE)[7] and provides a summary for the results.

rsid	position	nearest gene	credible set size	interval (bp)	start (bp)	end (bp)
rs3820282	1:22141722	*WNT4*	6	45495	22096228	22141722
rs61807787	1:172162145	*DNM3*	106	248185	171970150	172218334
rs4149909	1:241860596	*EXO1*	13	18808	241848042	241866849
rs2183478	1:244151650	*ZBTB18*	2	61549	244151650	244213198
rs4335411	1:248897507	*PGBD2*	4	35798	248861710	248897507
rs35417544	2:11540277	*GREB1*	24	23381	11522052	11545432
rs74576866	2:28106534	*BABAM2*	20	142094	28077447	28219540
rs17631680	2:66863235	*MEIS1*	1	1	66863235	66863235
rs13392042	2:99454113	*REV1*	138	204163	99314447	99518609
rs10804157	2:207258660	*MYOSLID*	7	608387	207257277	207865663
rs34766121	2:241720139	*ING5*	2	6294	241720139	241726432
rs3804984	3:4674530	*ITPR1*	1	1	4674530	4674530
rs1010961	3:24213259	*THRB*	7	9101	24209772	24218872
rs35701251	3:27488262	*SLC4A7*	107	133939	27377371	27511309
rs35446936	3:169768720	*ACTRT3*	27	51018	169759718	169810735
rs13060777	3:185807411	*IGF2BP2*	58	52911	185770515	185823425
rs62323682	4:53684007	*LNX1*	14	49637	53634371	53684007
rs12640488	4:69735020	*SULT1B1*	32	123121	69658940	69782060
rs2452597	4:94572095	*PDLIM5*	41	136859	94537299	94674157
rs1037475	4:99031559	*METAP1*	32	95051	98961982	99057032
rs2242652	5:1279913	*TERT*	2	239	1279675	1279913
rs4367292	5:133099880	*HSPA4*	83	70426	133039643	133110068
rs2456181	5:177023836	*ZNF346*	55	170805	176896297	177067101
rs41269026	6:34240996	*HMGA1*	57	196791	34197574	34394364
rs10456443	6:36653670	*CDKN1A*	16	9344	36650363	36659706
rs11153158	6:109054915	*SESN1*	57	95237	108960208	109055444
rs58415480	6:152241136	*SYNE1*	1	1	152241136	152241136
rs4723230	7:33008785	*FKBP9*	103	81254	33006946	33088199
rs2270206	7:117273513	*WNT2*	3	5825	117267689	117273513
rs12706314	7:121132432	*CPED1*	60	407085	120818924	121226008
rs35908158	7:130935964	*LINC-PINT*	9	12539	130926493	130939031
rs13275869	8:30452819	*RBPMS*	25	353697	30196778	30550474
rs1516980	8:128506035	*LINC00824*	134	220942	128298931	128519872
rs10815466	9:680714	*KANK1*	13	138136	667680	805815
rs28508285	9:89639982	*GADD45G*	3	138334	89501649	89639982
rs946711	10:21517903	*SKIDA1*	35	205612	21494705	21700316
rs72784785	10:31678920	*ZEB1*	34	56512	31624962	31681473
rs1426619	10:88331783	*RNLS*	71	169136	88294511	88463646
rs4387287	10:103918139	*STN1*	7	7443	103915096	103922538
rs7103852	11:197557	*ODF3,BET1L*	16	67925	197557	265481
rs11031006	11:30204981	*FSHB*	18	118370	30204809	30323178
rs2057178	11:32342641	*WT1*	9	23547	32325288	32348834
rs2553773	11:35062086	*PDHX*	27	14634	35062086	35076719
rs149934734	11:108444879	*C11orf65*	4	213682	108272729	108486410
rs10891420	11:112703765	*LOC105369496*	1	1	112703765	112703765
rs2131371	12:46402739	*SLC38A2*	68	73721	46389870	46463590
rs11178393	12:70756878	*PTPRR*	10	12875	70751752	70764626
rs28583837	12:123379073	*KMT5A*	110	1265069	123129018	124394086
rs117245733	13:40149807	*FOXO1*	1	1	40149807	40149807
rs12148374	15:67922458	*SKOR1,PIAS1*	38	445721	67890105	68335825
rs12599260	16:50059327	*HEATR3*	46	75529	50028661	50104189
rs66998222	16:51447685	*AC007344.1*	51	1425558	50028661	51454218
rs78378222	17:7668434	*TP53*	1	1	7668434	7668434
rs12601765	17:12652500	*MYOCD*	7	14865	12643623	12658487
rs8105767	19:22032639	*ZNF257*	173	538899	21938656	22477554
rs16991615	20:5967581	*MCM8*	1	1	5967581	5967581
rs13039273	20:57441016	*CTCFL*	4	4528	57441016	57445543
rs75691080	20:63638397	*STMN3*	1	1	63638397	63638397
rs2834747	21:35072824	*RUNX1*	7	16610	35072753	35089362
rs9610482	22:36287509	*MYH9*	8	28715	36287509	36316223
rs112251865	22:40269221	*TNRC6B*	18	186456	40135641	40322096
rs5936604	23:70889039	*SLC7A3*	6	38152	70888397	70926548
rs5930554	23:132178061	*FRMD7*	16	80267	132117298	132197564

Table S4. Fine-mapping results of META-2.

We applied the FinnGen finemapping pipeline (available at https://github.com/FINNGEN/finemapping-pipeline, accessed on 2/2/2022) with default parameters. In brief, the pipeline calculates linkage disequilibrium within the regions of interest with LDstore2[6] using FinnGen samples, generates 99 % credible sets using the SUm of Single Effects (SuSiE)[7] and provides a summary for the results.

rsid	position	nearest gene	credible set size	interval (bp)	start (bp)	end (bp)
rs3820282	1:22141722	*WNT4*	6	45495	22096228	22141722
rs61807787	1:172162145	*DNM3*	73	215801	172002534	172218334
rs4149909	1:241860596	*EXO1*	2	10366	241860596	241870961
rs2183478	1:244151650	*ZBTB18*	2	93	244151558	244151650
rs4335411	1:248897507	*PGBD2*	4	45077	248861710	248906786
rs10929757	2:11562535	*GREB1*	1	1	11562535	11562535
rs4637064	2:28213441	*BABAM2*	61	1181058	27156503	28337560
rs17631680	2:66863235	*MEIS1*	1	1	66863235	66863235
rs1451246	2:99447821	*REV1*	69	178093	99314447	99492539
rs10804157	2:207258660	*MYOSLID*	8	8720	207257277	207265996
rs6437284	2:241710378	*ING5*	37	61383	241667096	241728478
rs3804984	3:4674530	*ITPR1*	1	1	4674530	4674530
rs2017200	3:24213261	*THRB*	10	33694	24185179	24218872
rs35701251	3:27488262	*NEK10*	99	133939	27377371	27511309
rs35446936	3:169768720	*TERC*	26	51018	169759718	169810735
rs66513933	3:185805582	*IFG2BP2*	64	59017	185770515	185829531
rs4864806	4:53634371	*LNX1*	28	49637	53634371	53684007
rs4694227	4:69658940	*SULT1B1*	23	123121	69658940	69782060
rs2452597	4:94572095	*PDLIM5*	42	126001	94548157	94674157
rs10069690	5:1279675	*TERT*	1	1	1279675	1279675
rs2456181	5:177023836	*ZNF346*	62	170805	176896297	177067101
rs12196819	6:108976313	*ARMC2*	160	98740	108956705	109055444
rs58415480	6:152241136	*SYNE1*	1	1	152241136	152241136
rs4730982	7:120807406	*TSPAN12*	41	109929	120770147	120880075
rs4545054	8:30444949	*RBPMS*	24	107076	30443399	30550474
rs11786929	8:129467485	*GSDMC*	9	18602	129448884	129467485
rs10815466	9:680714	*KANK1*	3	2717	680714	683430
rs1243192	10:21695031	*MLLT10*	30	205612	21494705	21700316
rs7090544	10:31666087	*ZEB1*	36	61057	31624962	31686018
rs2082415	10:76884502	*KCNMA1*	4410	2999032	75385239	78384270
rs189195982	10:101726828	*FGF8*	12	433026	101726828	102159853
rs75731980	10:102788270	*WBP1L*	3	160004	102788270	102948273
rs182218101	10:103873824	*OBFC1*	2	29588	103844237	103873824
rs17119191	10:105587387	*SORCS3*	15	642130	105095632	105737761
rs1336619	10:106822067	*SORCS1*	24	582458	106329275	106911732
rs7103852	11:197557	*ODF3*	16	67925	197557	265481
rs11031006	11:30204981	*FSHB*	18	118370	30204809	30323178
rs11031731	11:32343884	*WT1*	2	14004	32329881	32343884
rs2553772	11:35063906	*PDHX*	26	20058	35056662	35076719
rs149934734	11:108444879	*C11orf65*	4	213682	108272729	108486410
rs10891420	11:112703765	*LOC105369496*	28	29522	112693680	112723201
rs1472178	12:46410392	*SLC38A2*	79	73721	46389870	46463590
rs142808358	12:68692314	*NUP107*	3	243755	68564481	68808235
rs11178393	12:70756878	*PTPRR*	18	27874	70751752	70779625
rs28576953	12:123405729	*KMT5A*	136	320052	123108835	123428886
rs149775438	13:38939164	*STOML3*	2	399980	38939164	39339143
rs117245733	13:40149807	*FOXO1*	1	1	40149807	40149807
rs2306022	15:68335825	*ITGA11*	13	17113	68318713	68335825
rs62033024	16:50056732	*HEATR3*	67	112294	49991896	50104189
rs66998222	16:51447685	*AC007344.1*	9	21780	51432439	51454218
rs78378222	17:7668434	*TP53*	1	1	7668434	7668434
rs74876186	17:8767403	*SPDYE4*	2	130623	8636781	8767403
rs12449987	17:12656880	*MYOCD*	27	15266	12643623	12658888
rs8105767	19:22032639	*ZNF257*	216	570782	21906773	22477554
rs16991615	20:5967581	*MCM8*	1	1	5967581	5967581
rs75691080	20:63638397	*STMN3*	16	61305	63577093	63638397
rs71758825	22:28779069	*CCDC117*	124	874010	28224596	29098605
rs9610482	22:36287509	*MYH9*	8	28715	36287509	36316223
rs112251865	22:40269221	*TNRC6B*	15	186456	40135641	40322096
rs5936980	X:70762863	*TEX11*	8	163686	70762863	70926548
rs5930554	X:132140742	*FRMD7*	19	80267	132117298	132197564

cs_size: the size of the credible set

Table S5. Fine-mapping results of the secondary signals.

We applied the FinnGen finemapping pipeline (available at https://github.com/FINNGEN/finemapping-pipeline, accessed on 2/2/2022) with default parameters. In brief, the pipeline calculates linkage disequilibrium within the regions of interest with LDstore2[6] using FinnGen samples, generates 99 % credible sets using the SUm of Single Effects (SuSiE)[7] and provides a summary for the results.

cond.	rsid	position	nearest gene	credible set size	interval (bp)	start (bp)	end (bp)
1	rs13407702	2:11515558	*GREB1*	17	58841	11475067	11533908
1	rs7584910	2:207848303	*PLEKHM3*	48	138584	207819617	207958200
1	rs11735529	4:52992781	*SCFD2*	16	35728	52957054	52992781
2	rs188378292	4:53301527	*SCFD2*	1	1	53301527	53301527
1	rs41292311	4:69750098	*SULT1B1*	3	35313	69738255	69773567
1	rs9397523	6:152551137	*SYNE1*	1	1	152551137	152551137
2	rs6901631	6:152245912	*SYNE1*	11	28999	152217850	152246848
1	rs35469085	9:806912	*DMRT1*	14	9155	804231	813385
2	rs7856727	9:854375	*DMRT1*	1	1	854375	854375
1	rs4387287	10:103918139	*OBFC1*	6	6351	103916188	103922538
2	rs188848367	10:103895645	*OBFC1*	16	1515494	102560691	104076184
1	rs12222188	11:224063	*SIRT3*	17	22829	202017	224845
2	rs2038318	11:32325691	*WT1*	1	1	32325691	32325691
1	rs2207548	11:32347198	*WT1*	3	8523	32338676	32347198
1	rs1519074	11:108177255	*NPAT*	27	382360	108112369	108494728
1	rs7986407	13:40605661	*FOXO1*	13	74954	40573021	40647975
2	rs9548898	13:39726408	*COG6*	68	116924	39661542	39778465
3	rs6563799	13:40162152	*FOXO1*	3	4292	40160593	40164884
4	rs9576914	13:39898446	*COG6*	45	29226	39898446	39927671
1	rs2058814	16:50048520	*CNEP1R1*	1	1	50048520	50048520
1	rs138420351	17:7796745	*DNAH2*	1	1	7796745	7796745

cond: the number of variants the signal is conditioned for

Table S6. RegulomeDB annotation of the association lead variants near *MYOCD*.
The RegulomeDB probability score is ranging from 0 to 1, with 1 being most likely to be a regulatory variant. 'Ranking' refers to following evidence: 1a, eQTL + TF binding + matched TF motif + matched DNase Footprint + DNase peak; 1b, eQTL + TF binding + any motif + DNase Footprint + DNase peak; 1c, eQTL + TF binding + matched TF motif + DNase peak; 1d, eQTL + TF binding + any motif + DNase peak; 1e, eQTL + TF binding + matched TF motif; 1f, eQTL + TF binding / DNase peak; 2a, TF binding + matched TF motif + matched DNase Footprint + DNase peak; 2b, TF binding + any motif + DNase Footprint + DNase peak; 2c, TF binding + matched TF motif + DNase peak; 3a, TF binding + any motif + DNase peak; 3b, TF binding + matched TF motif; 4, TF binding + DNase peak; 5, TF binding or DNase peak; 6, Motif hit; 7, Other. RegulomeDB ranking < 3 and/or probability score > 0.8 can be considered as evidence for altered regulatory consequences. Data was downloaded from RegulomeDB 02/10/2021.

Rsids	Probability	Ranking
rs11871444	0.60906	4
rs12601765	0.0	5
rs12601724	0.13454	5
rs4792271	0.00167	6
rs4792272	0.13454	5
rs11870377	0.58955	5
rs72811248	0.58955	5

Table S7. RegulomeDB annotation of the association lead variants near *MYOSLID*.
The RegulomeDB probability score is ranging from 0 to 1, with 1 being most likely to be a regulatory variant. ‘Ranking’ refers to following evidence: 1a, eQTL + TF binding + matched TF motif + matched DNase Footprint + DNase peak; 1b, eQTL + TF binding + any motif + DNase Footprint + DNase peak; 1c, eQTL + TF binding + matched TF motif + DNase peak; 1d, eQTL + TF binding + any motif + DNase peak; 1e, eQTL + TF binding + matched TF motif; 1f, eQTL + TF binding / DNase peak; 2a, TF binding + matched TF motif + matched DNase Footprint + DNase peak; 2b, TF binding + any motif + DNase Footprint + DNase peak; 2c, TF binding + matched TF motif + DNase peak; 3a, TF binding + any motif + DNase peak; 3b, TF binding + matched TF motif; 4, TF binding + DNase peak; 5, TF binding or DNase peak; 6, Motif hit; 7, Other. RegulomeDB ranking < 3 and/or probability score > 0.8 can be considered as evidence for altered regulatory consequences. Data was downloaded from RegulomeDB 02/10/2021.

Rsids	Probability	Ranking
rs6758102	0.60906	4
rs10804157	1.0	2b
rs6709320	0.18412	7
rs2111595	0.60906	4
rs11694764	0.18412	7
rs56167178	0.13454	5

Table S8. ChIP-seq data of rs10804157.
The variant rs10804157 showed the highest probability (1.0) of being a regulatory variant among the variants showing genome-wide significant ($p<5x10^{-8}$) association with UL at 2q33.3. ChIP-seq data for this variant was extracted from RegulomeDB on 02/10/2021.

Method	Peak location	Biosample	Targets	Organ	Dataset	File	Value	Strand
ChIP-seq	chr2:208123084..208123749	A549	SP1	lung	ENCSR000BPE	ENCFF348RKC	326.58420	-
ChIP-seq	chr2:208123107..208123712	A549	EP300	lung	ENCSR000BPW	ENCFF002CFV	287.620948909819	-
ChIP-seq	chr2:208123262..208123522	A549	NR3C1	lung	ENCSR000BHF	ENCFF648ZNE	244.46238	-
ChIP-seq	chr2:208123094..208123709	A549	TCF12	lung	ENCSR000BQQ	ENCFF672FQU	221.07448	-
ChIP-seq	chr2:208123378..208123656	MCF-7	FOS	mammary gland	ENCSR569XNP	ENCFF965AIZ	206.29520	-
ChIP-seq	chr2:208123332..208123669	A549	FOSL2	lung	ENCSR000BQO	ENCFF374ZCG	187.18388	-
ChIP-seq	chr2:208123364..208123572	A549	FOXA1	lung	ENCSR000BRD	ENCFF755AXA	170.22291	-
ChIP-seq	chr2:208123171..208123507	MCF-7	ESRRA	mammary gland	ENCSR954WVZ	ENCFF044NEB	156.03072	-
ChIP-seq	chr2:208123287..208123667	A549	MAFK	lung	ENCSR541WQI	ENCFF530ZTE	139.13663	-
ChIP-seq	chr2:208123361..208123595	HEK293	ZBTB17	kidney	ENCSR631WAA	ENCFF047LUI	114.24409	-
ChIP-seq	chr2:208123179..208123669	MCF-7	ZNF217	mammary gland	ENCSR465XQW	ENCFF567MGV	99.76437	-
ChIP-seq	chr2:208123231..208123661	MCF-7	ZNF579	mammary gland	ENCSR018MQH	ENCFF854CXA	98.09751	-
ChIP-seq	chr2:208123265..208123545	A549	NR3C1	lung	ENCSR000BHG	ENCFF913WFD	97.82031	-
ChIP-seq	chr2:208123181..208123801	A549	SIN3A	lung	ENCSR513XQX	ENCFF172VGB	94.53579	-
ChIP-seq	chr2:208123210..208123666	MCF-7	MNT	mammary gland	ENCSR663ZZZ	ENCFF628SBD	84.80959	-
ChIP-seq	chr2:208123135..208123675	A549	BCL3	lung	ENCSR000BQH	ENCFF325WAV	84.05348	-
ChIP-seq	chr2:208123311..208123581	IMR-90	MAFK	lung	ENCSR000EFH	ENCFF593MEB	83.58383	-
ChIP-seq	chr2:208123232..208123622	MCF-7	FOXA1	mammary gland	ENCSR126YEB	ENCFF596OJV	82.25613	-
ChIP-seq	chr2:208123208..208123618	MCF-7	CTBP1	mammary gland	ENCSR636EYA	ENCFF785ZQF	81.59685	-
ChIP-seq	chr2:208123147..208123591	liver	RXRA	liver	ENCSR098XMN	ENCFF189HPG	75.54675	-
ChIP-seq	chr2:208123227..208123697	A549	ZBTB33	lung	ENCSR000BPZ	ENCFF639NGJ	68.14892	-
ChIP-seq	chr2:208123237..208123541	A549	NR3C1	lung	ENCSR000BJR	ENCFF080RMP	63.97341	-
ChIP-seq	chr2:208123188..208123618	HEK293	KLF1	kidney	ENCSR859BMR	ENCFF620ZDF	61.18339	-
ChIP-seq	chr2:208123153..208123417	A549	ESRRA	lung	ENCSR473SUA	ENCFF266ORV	60.61061	-
ChIP-seq	chr2:208123318..208123702	MCF-7	MAZ	mammary gland	ENCSR288IJC	ENCFF492DZG	58.62570	-
ChIP-seq	chr2:208123059..208123459	endothelial cell of umbilical vein	FOS	vein, vasculature, epithelium, blood vessel	ENCSR000EVU	ENCFF390MXI	56.59256	-
ChIP-seq	chr2:208123235..208123645	MCF-7	NFIB	mammary gland	ENCSR702BYX	ENCFF817RST	56.19887	-
ChIP-seq	chr2:208123294..208123578	IMR-90	BHLHE40	lung	ENCSR957KYB	ENCFF942FBW	53.93919	-
ChIP-seq	chr2:208122889..208123613	K562	FOSL1	blood, bodily fluid	ENCSR239ZLZ	ENCFF692PVV	52.28088	-
ChIP-seq	chr2:208123124..208123544	liver	NR2F2	liver	ENCSR338MMB	ENCFF266KLW	50.28680	-
ChIP-seq	chr2:208123248..208123738	A549	REST	lung	ENCSR000BQP	ENCFF136RBA	49.93059	-
ChIP-seq	chr2:208123340..208123630	A549	ZFP36	lung	ENCSR294JWV	ENCFF418DRY	49.62579	-
ChIP-seq	chr2:208123096..208123416	SK-N-SH	JUND	brain	ENCSR000BSK	ENCFF598ZLV	49.00620	-
ChIP-seq	chr2:208123233..208123757	A549	YY1	lung	ENCSR000BPM	ENCFF713ISJ	48.03768	-
ChIP-seq	chr2:208123332..208124008	22Rv1	CTCF	prostate gland	ENCSR857PBV	ENCFF030BPR	44.66346	-
ChIP-seq	chr2:208123056..208123476	liver	JUND	liver	ENCSR837GTK	ENCFF804UDG	44.66249	-
ChIP-seq	chr2:208123048..208123638	MCF-7	FOSL2	mammary gland	ENCSR546KCN	ENCFF331KNS	43.40140	-
ChIP-seq	chr2:208123246..208123730	A549	ATF3	lung	ENCSR000BPS	ENCFF913MUA	43.04867	-

Method	Peak location	Biosample	Targets	Organ	Dataset	File	Value	Strand
ChIP-seq	chr2:208123340..208124040	22Rv1	CTCF	prostate gland	ENCSR847XGE	ENCFF259XYR	41.50506	-
ChIP-seq	chr2:208123167..208123671	liver	SP1	liver	ENCSR386YIH	ENCFF017YUI	41.36437	-
ChIP-seq	chr2:208123257..208123587	T47D	EP300	mammary gland	ENCSR000BLM	ENCFF002CNZ	39.636132175516	-
ChIP-seq	chr2:208123176..208123640	liver	RXRA	liver	ENCSR352QSB	ENCFF651LIG	39.78583	-
ChIP-seq	chr2:208123138..208123628	liver	NR2F2	liver	ENCSR168SMX	ENCFF168INE	38.88749	-
ChIP-seq	chr2:208123233..208123877	HEK293	TRIM28	kidney	ENCSR618HNF	ENCFF034GYQ	37.81934	-
ChIP-seq	chr2:208123305..208123735	MCF-7	ZNF687	mammary gland	ENCSR899BKM	ENCFF955YVT	37.18623	-
ChIP-seq	chr2:208123250..208123560	MCF-7	MYC	mammary gland	ENCSR000DMQ	ENCFF695IQU	37.15063	-
ChIP-seq	chr2:208123277..208123697	A549	ELF1	lung	ENCSR000BPT	ENCFF533NIV	37.00036	-
ChIP-seq	chr2:208122967..208123727	MCF-7	KLF4	mammary gland	ENCSR265WJC	ENCFF463ZWH	34.95441	-
ChIP-seq	chr2:208123205..208123815	IMR-90	POLR2A	lung	ENCSR000EFK	ENCFF002CVJ	33.0223703871219	-
ChIP-seq	chr2:208123269..208123669	endothelial cell of umbilical vein	FOS	vein, vasculature, epithelium, blood vessel	ENCSR000EVU	ENCFF390MXI	32.41105	-
ChIP-seq	chr2:208123383..208123623	IMR-90	NFE2L2	lung	ENCSR197WGI	ENCFF641RNZ	31.19285	-
ChIP-seq	chr2:208123247..208123678	Caco-2	HNF4A	intestine, large intestine	TSTSR342350	TSTFF265946	30	-
ChIP-seq	chr2:208123293..208123583	T47D	GATA3	mammary gland	ENCSR000BMX	ENCFF058XAD	29.56353	-
ChIP-seq	chr2:208123281..208123601	A549	NR3C1	lung	ENCSR000BJT	ENCFF495YRW	29.31897	-
ChIP-seq	chr2:208123261..208123531	A549	NR3C1	lung	ENCSR000BHE	ENCFF841IFL	29.14627	-
ChIP-seq	chr2:208123248..208123838	MCF-7	FOSL2	mammary gland	ENCSR546KCN	ENCFF331KNS	29.01817	-
ChIP-seq	chr2:208123298..208123598	MCF 10A	MYC	mammary gland	ENCSR000DOM	ENCFF002CZA	24.1612783298454	-
ChIP-seq	chr2:208123121..208123685	IMR-90	CHD1	lung	ENCSR000EFC	ENCFF441IFB	24.73066	-
ChIP-seq	chr2:208123247..208123517	A549	USF1	lung	ENCSR000BHX	ENCFF002CGC	23.8430557308026	-
ChIP-seq	chr2:208123084..208123749	A549	SP1	lung	ENCSR000BPE	ENCFF348RKC	23.40951	-
ChIP-seq	chr2:208123079..208123399	HeLa-S3	JUND	uterus	ENCSR000EDH	ENCFF002CSP	22.3246370112007	-
ChIP-seq	chr2:208123359..208123703	GM12878	BCL3	blood, bodily fluid	ENCSR000BNQ	ENCFF625SHY	20.58068	-
ChIP-seq	chr2:208123138..208123394	HeLa-S3	FOS	uterus	ENCSR000EZE	ENCFF002CSB	19.8552135147245	-
ChIP-seq	chr2:208123318..208123638	SK-N-SH	JUND	brain	ENCSR000BSK	ENCFF598ZLV	19.05498	-
ChIP-seq	chr2:208123375..208123631	A549	USF1	lung	ENCSR000BJB	ENCFF002CGD	18.2821813113775	-
ChIP-seq	chr2:208123323..208123739	Caco-2	CDX2	intestine, large intestine	TSTSR586454	TSTFF910843	18	-
ChIP-seq	chr2:208123114..208123530	liver	ATF3	liver	ENCSR480LIS	ENCFF975PTM	17.59478	-
ChIP-seq	chr2:208122865..208123405	MCF 10A	POLR2A	mammary gland	ENCSR000DPA	ENCFF755ZCF	17.14568	-
ChIP-seq	chr2:208123302..208123558	MCF 10A	STAT3	mammary gland	ENCSR000DOZ	ENCFF807JAC	14.95877	-
ChIP-seq	chr2:208123094..208123714	A549	TCF12	lung	ENCSR000BQQ	ENCFF672FQU	11.69204	-
ChIP-seq	chr2:208123257..208123677	liver	JUND	liver	ENCSR837GTK	ENCFF804UDG	10.18840	-

Table S9. RegulomeDB annotation of the association lead variants near *CDKN1A*.

The RegulomeDB probability score is ranging from 0 to 1, with 1 being most likely to be a regulatory variant. 'Ranking' refers to following evidence: 1a, eQTL + TF binding + matched TF motif + matched DNase Footprint + DNase peak; 1b, eQTL + TF binding + any motif + DNase Footprint + DNase peak; 1c, eQTL + TF binding + matched TF motif + DNase peak; 1d, eQTL + TF binding + any motif + DNase peak; 1e, eQTL + TF binding + matched TF motif; 1f, eQTL + TF binding / DNase peak; 2a, TF binding + matched TF motif + matched DNase Footprint + DNase peak; 2b, TF binding + any motif + DNase Footprint + DNase peak; 2c, TF binding + matched TF motif + DNase peak; 3a, TF binding + any motif + DNase peak; 3b, TF binding + matched TF motif; 4, TF binding + DNase peak; 5, TF binding or DNase peak; 6, Motif hit; 7, Other. RegulomeDB ranking < 3 and/or probability score > 0.8 can be considered as evidence for altered regulatory consequences. Data was downloaded from RegulomeDB 02/10/2021.

Rsids	Probability	Ranking
rs6457932	0.60906	4
rs9349003	0.49417	3a
rs9366912	0.55436	1f
rs112297876	0.58955	5
rs12524435	0.38284	5
rs10947616	0.58955	5
rs4713993	0.13454	5
rs6915170	0.30943	6
rs4464789	0.6893	3a
rs10456441	0.49737	3a
rs10456442	0.60906	4
rs10456443	0.61652	2b
rs12210094	0.60906	4
rs12528913	0.08	5
rs12530170	0.84573	2b
rs12203953	0.96944	1a
rs6920453	0.60906	4
rs75420281	0.60906	4
rs3176348	0.86655	2b
rs150499251	0.58955	5
rs9357241	0.04578	5
rs6921165	0.60906	4
rs9394413	0.98162	5
rs7741267	0.60906	4
rs7751412	0.30476	3a

Table S10. Results of MAGMA enrichment analysis.

We conducted MAGMA[4] gene-set enrichment analysis as implemented in FUMA[5] using genome-wide summary statistics from META-2. Gene-based p-values were computed for protein-coding genes by mapping variants to genes with default parameters, 1000 Genomes European population, and SNP-wide mean model, and subsequent enrichment analyses were performed for the significant genes using 4728 curated gene sets and 6166 GO terms as reported in MsigDB[8]. The table shows the results for the gene sets significant after false discovery rate (FDR) correction (p_{FDR}<0.05).

Gene_set	N_{genes}	BETA	SE	P	P_{FDR}
GO_bp:go_regulation_of_male_gonad_development	8	2.34	0.35	1.15E-11	1.78E-07
GO_cc:go_telomere_cap_complex	13	1.43	0.26	1.39E-08	0.00011
GO_bp:go_regulation_of_nuclear_transcribed_mrna_catabolic_process_deadenylation_dependent_decay	20	1.15	0.22	1.03E-07	0.00053
GO_bp:go_regulation_of_gonad_development	18	1.10	0.22	4.52E-07	0.0017
GO_bp:go_sex_determination	21	0.86	0.18	1.73E-06	0.0048
Curated_gene_sets:pid_angiopoietin_receptor_pathway	49	0.60	0.13	1.85E-06	0.0048
GO_bp:go_positive_regulation_of_heart_growth	37	0.74	0.16	2.33E-06	0.0052
GO_mf:go_translation_regulator_activity	132	0.32	0.07	3.48E-06	0.0063
Curated_gene_sets:nikolsky_breast_cancer_5p15_amplicon	26	1.08	0.24	3.80E-06	0.0063
Curated_gene_sets:pid_telomerase_pathway	68	0.49	0.11	4.57E-06	0.0063
GO_bp:go_respiratory_system_development	195	0.31	0.07	4.87E-06	0.0063
GO_bp:go_kidney_mesenchyme_development	19	1.06	0.24	4.91E-06	0.0063
GO_bp:go_metanephric_mesenchyme_development	15	1.18	0.27	5.81E-06	0.0065
GO_bp:go_positive_regulation_of_gonad_development	10	1.50	0.34	5.89E-06	0.0065
GO_bp:go_posttranscriptional_regulation_of_gene_expression	581	0.16	0.04	1.03E-05	0.0097
GO_bp:go_type_i_pneumocyte_differentiation	5	2.09	0.49	1.04E-05	0.0097
GO_bp:go_diaphragm_development	9	1.43	0.34	1.06E-05	0.0097
Curated_gene_sets:wu_hbx_targets_3_dn	13	1.05	0.25	1.58E-05	0.013
Curated_gene_sets:tsai_dnajb4_targets_dn	6	1.72	0.41	1.60E-05	0.013
GO_bp:go_regulation_of_cell_cycle	1151	0.10	0.03	3.82E-05	0.028
GO_bp:go_positive_regulation_of_organ_growth	48	0.55	0.14	3.91E-05	0.028
Curated_gene_sets:pid_plk1_pathway	44	0.53	0.13	3.99E-05	0.028
GO_bp:go_cell_proliferation_involved_in_metanephros_development	10	1.34	0.34	4.28E-05	0.028
Curated_gene_sets:lindvall_immortalized_by_tert_up	65	0.46	0.12	4.32E-05	0.028
GO_bp:go_connective_tissue_development	266	0.22	0.06	4.81E-05	0.030
Curated_gene_sets:reactome_cellular_senescence	171	0.27	0.07	5.20E-05	0.031
GO_bp:go_chondroblast_differentiation	5	1.44	0.37	5.45E-05	0.031
GO_bp:go_regulation_of_biomineral_tissue_development	86	0.40	0.10	5.60E-05	0.031
Curated_gene_sets:biocarta_egfr_smrte_pathway	11	1.17	0.30	5.75E-05	0.031
GO_bp:go_regulation_of_nuclear_transcribed_mrna_poly_a_tail_shortening	14	1.06	0.28	5.95E-05	0.031
Curated_gene_sets:pilon_klf1_targets_dn	1980	0.08	0.02	6.12E-05	0.031
GO_bp:go_positive_regulation_of_cell_cycle	380	0.17	0.04	7.11E-05	0.034
GO_bp:go_cellular_response_to_starvation	146	0.27	0.07	7.71E-05	0.036
GO_bp:go_regulation_of_epidermis_development	82	0.41	0.11	8.89E-05	0.040
GO_bp:go_regulation_of_heart_growth	63	0.44	0.12	9.49E-05	0.041
GO_bp:go_regulation_of_cellular_amide_metabolic_process	375	0.17	0.04	9.65E-05	0.041
GO_bp:go_positive_regulation_of_mrna_catabolic_process	47	0.47	0.13	9.82E-05	0.041
GO_bp:go_negative_regulation_of_gonad_development	6	1.38	0.37	0.00010	0.042
Curated_gene_sets:biocarta_rna_pathway	9	0.84	0.23	0.00011	0.042
GO_bp:go_cellular_response_to_extracellular_stimulus	259	0.20	0.05	0.00012	0.047
GO_bp:go_cell_cycle	1766	0.08	0.02	0.00013	0.048
Curated_gene_sets:abdelmohsen_elavl4_targets	16	0.79	0.22	0.00013	0.049
Curated_gene_sets:reactome_runx3_regulates_cdkn1a_transcription	7	1.45	0.40	0.00014	0.049
GO_bp:go_adrenal_gland_development	24	0.74	0.20	0.00014	0.049
GO_bp:go_negative_regulation_of_telomere_maintenance_via_telomerase	20	0.78	0.21	0.00014	0.049
GO_mf:go_dna_binding_transcription_factor_activity	1661	0.09	0.02	0.00015	0.049
GO_bp:go_male_sex_determination	13	0.89	0.25	0.00015	0.049
GO_bp:go_positive_regulation_of_cardiac_muscle_cell_proliferation	25	0.72	0.20	0.00015	0.049
GO_bp:go_stress_induced_premature_senescence	8	1.06	0.29	0.00016	0.049
GO_bp:go_organ_growth	176	0.25	0.07	0.00016	0.049

Table S11. Genetic correlations of UL with 20 metabolic and anthropometric traits.
We used LDSC software[2] to estimate genetic correlations (r_g) of UL with 20 metabolic and anthropometric traits extracted from the GWAS database provided by the MRC Integrative Epidemiology Unit (https://gwas.mrcieu.ac.uk/). For UL, genome-wide data from META-2 was used in the r_g estimation. We run LDSC using standard settings described at https://github.com/bulik/ldsc/wiki/Heritability-and-Genetic-Correlation. Genetic correlations passing false discovery rate (fdr) correction were deemed significant (p_{fdr}<0.05).

Trait	Trait ID	Sample size	N SNPs	r_g	se	p	p_{fdr}
Triglycerides	ieu-b-111	441016	12321875	0.1609	0.0276	5.52E-09	1.10E-07
Waist circumference	ukb-b-9405	462166	9851867	0.1012	0.0243	3.08E-05	1.23E-04
Diastolic blood pressure	ieu-b-39	757601	7160619	0.0978	0.0248	8.27E-05	2.76E-04
Waist-to-hip ratio	ieu-a-72	224459	2562516	0.0953	0.0381	0.012	0.020
Body mass index (BMI)	ukb-b-19953	461460	9851867	0.0912	0.0250	0.0003	7.63E-04
Basal metabolic rate	ukb-b-16446	454874	9851867	0.0841	0.0251	0.0008	0.002
Whole body water mass	ukb-b-14540	454888	9851867	0.0832	0.0253	0.001	0.002
Whole body fat-free mass	ukb-b-13354	454850	9851867	0.0810	0.0252	0.001	0.003
Systolic blood pressure	ieu-b-38	757601	7088083	0.0605	0.0239	0.011	0.020
Whole body fat mass	ukb-b-19393	454137	9851867	0.0566	0.0238	0.017	0.026
Hip circumference	ukb-b-15590	462117	9851867	0.0513	0.0242	0.034	0.048
Total cholesterol	ieu-a-301	187365	2446982	0.0416	0.0399	0.297	0.371
Body fat percentage	ukb-b-8909	454633	9851867	0.0346	0.0242	0.153	0.204
Apolipoprotein B	ieu-b-108	439214	12321875	0.0126	0.0332	0.705	0.720
LDL cholesterol	ieu-b-110	440546	12321875	-0.0201	0.0367	0.584	0.649
C-reactive protein	bbj-a-14	75391	6108953	-0.0445	0.1240	0.720	0.720
Fasting blood glucose adjusted for BMI	ebi-a-GCST007858	33231	105585	-0.0882	0.1187	0.457	0.538
Apolipoprotein A-I	ieu-b-107	393193	12321875	-0.1100	0.0260	2.26E-05	1.13E-04
Impedance of whole body	ukb-b-19921	454840	9851867	-0.1299	0.0270	1.56E-06	1.04E-05
HDL cholesterol	ieu-b-109	403943	12321875	-0.1386	0.0245	1.48E-08	1.48E-07

Table S12. Results of the bi-directional two-sample Mendelian randomization.

Bi-directional two-sample Mendelian randomization of UL risk and 20 metabolic and anthropometric traits was completed using TwoSampleMR R library[12]. To avoid possible bias from overlapping samples, we extracted genetic instruments for UL from the GWAS results obtained in FinnGen, and for other, mostly UKBB-based traits, from the GWAS database provided by the MRC IEU and integrated in TwoSampleMR. LD pruning was completed using European population reference, threshold of r^2=0.001, and clumping window of 10 kb. We considered inverse variance weighted (IVW) method as the primary analysis for which false discovery rate (fdr) correction was applied to adjust for multiple comparisons. MR Egger analysis was considered exploratory and multiple testing correction was not applied.

Direction	Trait	Method	N_{SNP}	Causal estimate scale	Causal estimate	CI95L	CI95U	P	P_{FDR}	$P_{heterogeneity}$	Egger intercept	$P_{pleiotropy}$
Trait -> UL	apolipoprotein A-I || id:ieu-b-107	IVW	250	OR	0.97	0.89	1.05	0.4178	0.6693	3.70E-11		
Trait -> UL	apolipoprotein A-I || id:ieu-b-107	MR Egger	250	OR	1.00	0.88	1.15	0.9580		3.40E-11	-0.0015	0.4731
UL -> Trait	apolipoprotein A-I || id:ieu-b-107	IVW	26	beta	-0.0192	-0.0348	-0.0036	0.0156	0.0626	1.04E-10		
UL -> Trait	apolipoprotein A-I || id:ieu-b-107	MR Egger	26	beta	-0.0379	-0.0647	-0.0110	0.0107		2.40E-09	0.0031	0.1124
Trait -> UL	apolipoprotein B || id:ieu-b-108	IVW	167	OR	1.03	0.96	1.12	0.3912	0.6693	8.15E-05		
Trait -> UL	apolipoprotein B || id:ieu-b-108	MR Egger	167	OR	1.08	0.97	1.19	0.1758		8.87E-05	-0.0023	0.2829
UL -> Trait	apolipoprotein B || id:ieu-b-108	IVW	26	beta	-0.0036	-0.0169	0.0097	0.5970	0.7701	2.89E-05		
UL -> Trait	apolipoprotein B || id:ieu-b-108	MR Egger	26	beta	-0.0035	-0.0275	0.0206	0.7810		1.71E-05	-2.07E-05	0.9904
Trait -> UL	Basal metabolic rate || id:ukb-b-16446	IVW	487	OR	1.24	1.08	1.43	0.0022	0.0205	2.00E-39		
Trait -> UL	Basal metabolic rate || id:ukb-b-16446	MR Egger	487	OR	1.36	0.98	1.89	0.0673		1.67E-39	-0.0014	0.5563
UL -> Trait	Basal metabolic rate || id:ukb-b-16446	IVW	26	beta	0.0198	0.0009	0.0388	0.0401	0.1070	5.49E-55		
UL -> Trait	Basal metabolic rate || id:ukb-b-16446	MR Egger	26	beta	0.0386	0.0055	0.0717	0.0316		6.66E-51	-0.0031	0.1921
Trait -> UL	Body fat percentage || id:ukb-b-8909	IVW	351	OR	0.99	0.88	1.13	0.9379	0.9619	0.00066		
Trait -> UL	Body fat percentage || id:ukb-b-8909	MR Egger	351	OR	1.07	0.71	1.63	0.7370		0.00060	-0.0011	0.7052
UL -> Trait	Body fat percentage || id:ukb-b-8909	IVW	26	beta	-0.0041	-0.0139	0.0058	0.4183	0.6693	2.86E-05		
UL -> Trait	Body fat percentage || id:ukb-b-8909	MR Egger	26	beta	0.0042	-0.0132	0.0216	0.6369		4.97E-05	-0.0014	0.2686
Trait -> UL	Body mass index (BMI) || id:ukb-b-19953	IVW	399	OR	1.13	1.03	1.24	0.0084	0.0373	3.42E-06		
Trait -> UL	Body mass index (BMI) || id:ukb-b-19953	MR Egger	399	OR	1.14	0.89	1.46	0.3014		2.92E-06	-0.00011	0.9615
UL -> Trait	Body mass index (BMI) || id:ukb-b-19953	IVW	26	beta	0.0038	-0.0171	0.0246	0.7217	0.8248	2.15E-24		
UL -> Trait	Body mass index (BMI) || id:ukb-b-19953	MR Egger	26	beta	0.0253	-0.0111	0.0617	0.1852		2.49E-22	-0.0036	0.1734

Direction	Trait	Method	N_{SNP}	Causal estimate scale	Causal estimate	CI95L	CI95U	P	P_{FDR}	$P_{heterogeneity}$	Egger intercept	$P_{pleiotropy}$
Trait -> UL	C-Reactive protein level || id:ieu-b-35	IVW	52	OR	1.01	0.93	1.10	0.8251	0.9168	0.0024		
Trait -> UL	C-Reactive protein level || id:ieu-b-35	MR Egger	52	OR	0.99	0.87	1.11	0.8305		0.0020	0.0019	0.6141
UL -> Trait	C-Reactive protein level || id:ieu-b-35	IVW	19	beta	0.0090	-0.0173	0.0354	0.5016	0.7166	0.0275		
UL -> Trait	C-Reactive protein level || id:ieu-b-35	MR Egger	19	beta	0.0076	-0.1014	0.1166	0.8932		0.0190	0.0002	0.9786
Trait -> UL	diastolic blood pressure || id:ieu-b-39	IVW	415	OR	1.02	1.01	1.03	0.0026	0.0205	4.75E-21		
Trait -> UL	diastolic blood pressure || id:ieu-b-39	MR Egger	415	OR	1.01	0.99	1.04	0.3482		3.70E-21	0.00081	0.7317
UL -> Trait	diastolic blood pressure || id:ieu-b-39	IVW	24	beta	0.3687	0.1493	0.5882	0.0010	0.0205	2.08E-30		
UL -> Trait	diastolic blood pressure || id:ieu-b-39	MR Egger	24	beta	0.6254	0.2022	1.0486	0.0084		9.05E-28	-0.0386	0.1813
Trait -> UL	Fasting blood glucose adjusted for BMI || id:ebi-a-GCST007858	IVW	9	OR	0.91	0.62	1.34	0.6353	0.7701	0.019		
Trait -> UL	Fasting blood glucose adjusted for BMI || id:ebi-a-GCST007858	MR Egger	9	OR	0.88	0.34	2.28	0.8067		0.010	0.0011	0.9490
UL -> Trait	Fasting blood glucose adjusted for BMI || id:ebi-a-GCST007858	IVW	6	beta	0.0014	-0.0322	0.0349	0.9371	0.9619	0.4507		
UL -> Trait	Fasting blood glucose adjusted for BMI || id:ebi-a-GCST007858	MR Egger	6	beta	-0.0686	-0.1656	0.0284	0.2381		0.6528	0.0091	0.2065
Trait -> UL	HDL cholesterol || id:ieu-b-109	IVW	298	OR	0.89	0.82	0.97	0.0075	0.0373	3.21E-18		
Trait -> UL	HDL cholesterol || id:ieu-b-109	MR Egger	298	OR	0.99	0.86	1.13	0.8460		1.46E-17	-0.0040	0.0420
UL -> Trait	HDL cholesterol || id:ieu-b-109	IVW	26	beta	-0.0152	-0.0310	0.0005	0.0584	0.1461	3.85E-12		
UL -> Trait	HDL cholesterol || id:ieu-b-109	MR Egger	26	beta	-0.0389	-0.0652	-0.0126	0.0078		1.43E-09	0.0039	0.0431
Trait -> UL	Hip circumference || id:ukb-b-15590	IVW	377	OR	1.05	0.96	1.16	0.2888	0.5624	1.31E-07		
Trait -> UL	Hip circumference || id:ukb-b-15590	MR Egger	377	OR	0.97	0.75	1.26	0.8429		1.19E-07	0.0016	0.5255
UL -> Trait	Hip circumference || id:ukb-b-15590	IVW	26	beta	0.0114	-0.0106	0.0334	0.3093	0.5624	1.35E-27		
UL -> Trait	Hip circumference || id:ukb-b-15590	MR Egger	26	beta	0.0336	-0.0048	0.0721	0.0994		2.14E-25	-0.0037	0.1836
Trait -> UL	Impedance of whole body || id:ukb-b-19921	IVW	463	OR	0.79	0.69	0.91	0.0010	0.0205	3.80E-44		
Trait -> UL	Impedance of whole body || id:ukb-b-19921	MR Egger	463	OR	0.58	0.41	0.84	0.0034		1.89E-43	0.0049	0.0691
UL -> Trait	Impedance of whole body || id:ukb-b-19921	IVW	26	beta	-0.0135	-0.0389	0.0119	0.2970	0.5624	1.62E-77		
UL -> Trait	Impedance of whole body || id:ukb-b-19921	MR Egger	26	beta	-0.0342	-0.0791	0.0107	0.1487		7.37E-74	0.0034	0.2856
Trait -> UL	LDL cholesterol || id:ieu-b-110	IVW	144	OR	0.99	0.90	1.09	0.9099	0.9619	3.25E-06		
Trait -> UL	LDL cholesterol || id:ieu-b-110	MR Egger	144	OR	1.06	0.92	1.22	0.4124		4.13E-06	-0.0031	0.2217

Direction	Trait	Method	N_{SNP}	Causal estimate scale	Causal estimate	CI95L	CI95U	P	P_{FDR}	$P_{heterogeneity}$	Egger intercept	$P_{pleiotropy}$
UL -> Trait	LDL cholesterol || id:ieu-b-110	IVW	26	beta	-0.0029	-0.0149	0.0090	0.6296	0.7701	0.0013		
UL -> Trait	LDL cholesterol || id:ieu-b-110	MR Egger	26	beta	-0.0061	-0.0278	0.0155	0.5831		0.0009	0.0005	0.7289
Trait -> UL	systolic blood pressure || id:ieu-b-38	IVW	405	OR	1.01	1.00	1.01	0.0261	0.0948	1.34E-14		
Trait -> UL	systolic blood pressure || id:ieu-b-38	MR Egger	405	OR	1.01	1.00	1.03	0.0543		1.58E-14	-0.0027	0.2574
UL -> Trait	systolic blood pressure || id:ieu-b-38	IVW	23	beta	0.3458	-0.0854	0.7771	0.1160	0.2578	1.41E-38		
UL -> Trait	systolic blood pressure || id:ieu-b-38	MR Egger	23	beta	-0.0863	-0.9173	0.7446	0.8406		4.29E-36	0.0653	0.2479
Trait -> UL	Total cholesterol || id:ieu-a-301	IVW	79	OR	1.01	0.94	1.09	0.7106	0.8248	0.015		
Trait -> UL	Total cholesterol || id:ieu-a-301	MR Egger	79	OR	1.07	0.96	1.20	0.2351		0.018	-0.0041	0.2204
UL -> Trait	Total cholesterol || id:ieu-a-301	IVW	17	beta	0.0111	-0.0238	0.0460	0.5333	0.7356	0.1610		
UL -> Trait	Total cholesterol || id:ieu-a-301	MR Egger	17	beta	-0.0145	-0.1602	0.1312	0.8477		0.1276	0.0027	0.7272
Trait -> UL	Triglycerides || id:ieu-a-302	IVW	53	OR	1.09	0.97	1.22	0.1395	0.2936	2.17E-05		
Trait -> UL	Triglycerides || id:ieu-a-302	MR Egger	53	OR	1.12	0.94	1.34	0.2214		1.63E-05	-0.0018	0.6903
UL -> Trait	Triglycerides || id:ieu-a-302	IVW	17	beta	0.0078	-0.0234	0.0390	0.6239	0.7701	0.2008		
UL -> Trait	Triglycerides || id:ieu-a-302	MR Egger	17	beta	0.0628	-0.0637	0.1894	0.3460		0.1944	-0.0058	0.3929
Trait -> UL	Waist circumference || id:ukb-b-9405	IVW	326	OR	1.19	1.05	1.35	0.0052	0.0334	2.61E-09		
Trait -> UL	Waist circumference || id:ukb-b-9405	MR Egger	326	OR	1.25	0.88	1.77	0.2118		2.14E-09	-0.00077	0.7821
UL -> Trait	Waist circumference || id:ukb-b-9405	IVW	26	beta	0.0127	-0.0028	0.0282	0.1085	0.2554	4.49E-14		
UL -> Trait	Waist circumference || id:ukb-b-9405	MR Egger	26	beta	0.0287	0.0016	0.0558	0.0486		7.41E-13	-0.0027	0.1741
Trait -> UL	Waist-to-hip ratio || id:ieu-a-72	IVW	29	OR	1.26	1.01	1.57	0.0388	0.1070	0.320		
Trait -> UL	Waist-to-hip ratio || id:ieu-a-72	MR Egger	29	OR	1.42	0.52	3.89	0.4966		0.277	-0.0031	0.8070
UL -> Trait	Waist-to-hip ratio || id:ieu-a-72	IVW	19	beta	-0.0001	-0.0344	0.0342	0.9946	0.9946	0.0016		
UL -> Trait	Waist-to-hip ratio || id:ieu-a-72	MR Egger	19	beta	-0.0695	-0.2096	0.0705	0.3443		0.0021	0.0075	0.3306
Trait -> UL	Whole body fat mass || id:ukb-b-19393	IVW	387	OR	1.04	0.94	1.14	0.4634	0.7120	5.68E-05		
Trait -> UL	Whole body fat mass || id:ukb-b-19393	MR Egger	387	OR	1.17	0.90	1.52	0.2382		5.77E-05	-0.0023	0.3258
UL -> Trait	Whole body fat mass || id:ukb-b-19393	IVW	26	beta	0.0056	-0.0099	0.0210	0.4806	0.7120	1.41E-10		
UL -> Trait	Whole body fat mass || id:ukb-b-19393	MR Egger	26	beta	0.0242	-0.0023	0.0507	0.0862		3.41E-09	-0.0031	0.1084

Direction	Trait	Method	N_{SNP}	Causal estimate scale	Causal estimate	CI95L	CI95U	P	P_{FDR}	$P_{heterogeneity}$	Egger intercept	$P_{pleiotropy}$
Trait -> UL	Whole body fat-free mass || id:ukb-b-13354	IVW	486	OR	1.24	1.08	1.42	0.0024	0.0205	6.68E-33		
Trait -> UL	Whole body fat-free mass || id:ukb-b-13354	MR Egger	486	OR	1.40	1.01	1.93	0.0422		6.53E-33	-0.0018	0.4187
UL -> Trait	Whole body fat-free mass || id:ukb-b-13354	IVW	26	beta	0.0203	0.0014	0.0392	0.0353	0.1070	7.97E-62		
UL -> Trait	Whole body fat-free mass || id:ukb-b-13354	MR Egger	26	beta	0.0383	0.0052	0.0715	0.0328		1.26E-57	-0.0030	0.2097
Trait -> UL	Whole body water mass || id:ukb-b-14540	IVW	500	OR	1.22	1.06	1.40	0.0058	0.0334	3.19E-35		
Trait -> UL	Whole body water mass || id:ukb-b-14540	MR Egger	500	OR	1.33	0.97	1.84	0.0814		2.74E-35	-0.0014	0.5321
UL -> Trait	Whole body water mass || id:ukb-b-14540	IVW	26	beta	0.0204	0.0013	0.0396	0.0367	0.1070	1.13E-63		
UL -> Trait	Whole body water mass || id:ukb-b-14540	MR Egger	26	beta	0.0387	0.0050	0.0723	0.0336		2.24E-59	-0.0030	0.2108

Table S13. Results of multivariable MR.

The multivariable effects of whole-body fat-free mass, whole-body fat mass, and estradiol level on the risk of uterine leiomyomata (UL) were evaluated using TwoSampleMR R library[12]. We extracted genetic instruments for UL from the GWAS results obtained in FinnGen, for whole-body fat-free mass and fat mass from the UKBB as provided by the MRC IEU and integrated in TwoSampleMR, and for estradiol from a study by Ruth *et al*[13]. LD pruning was completed using European population reference, threshold of r^2=0.001, and clumping window of 10 kb.

exposure	outcome	n_{snp}	b	se	pval
Estradiol	Uterine leiomyomata	13	0.616	0.520	0.235
Whole body fat-free mass || id:ukb-b-13354	Uterine leiomyomata	415	0.271	0.114	0.018
Whole body fat mass || id:ukb-b-19393	Uterine leiomyomata	207	-0.061	0.106	0.567

Table S14. Results of outlier-corrected MR-PRESSO.

We derived causal estimates using outlier-corrected MR-PRESSO[14] for the traits showing a significant IVW-based causal effect on the risk of uterine leiomyomata. As in the primary analyses, we extracted genetic instruments from the GWAS completed in FinnGen and for other traits from the MRC-IEU database. Also, to ensure comparability and the usage of the same genetic instruments, LD pruning was completed as previously, *i.e.*, using European population reference, threshold of r^2=0.001, and clumping window of 10 kb. The analysis was considered exploratory and no multiple testing correction was applied.

Causal Estimate	Sd	T-stat	P-value	pheno	id.exposure
0.1634	0.0548	2.9802	0.0030	Basal metabolic rate	ukb-b-16446
0.1143	0.0430	2.6599	0.0081	BMI	ukb-b-19953
0.0112	0.0045	2.5060	0.0126	Diastolic BP	ieu-b-39
0.1685	0.0553	3.0460	0.0024	Fat-free mass	ukb-b-13354
-0.1244	0.0394	-3.1564	0.0018	HDL-C	ieu-b-109
-0.1610	0.0524	-3.0729	0.0022	Impedance	ukb-b-19921
0.1870	0.0581	3.2209	0.0014	Waist circumference	ukb-b-9405
0.1409	0.0562	2.5085	0.0124	Water mass	ukb-b-14540

Table S15. Results of MRMix.

We derived causal estimates using MRMix[15] method for the traits showing a significant IVW-based causal effect on the risk of uterine leiomyomata. As in the primary analyses, we extracted genetic instruments from the GWAS completed in FinnGen and for other traits from the MRC-IEU database. Also, to ensure comparability and the usage of the same genetic instruments, LD pruning was completed as previously, *i.e.*, using European population reference, threshold of r^2=0.001, and clumping window of 10 kb. The analysis was considered exploratory and no multiple testing correction was applied.

**theta**	**setheta**	**p**	**trait**	**Id.exposure**
0.11	0.089	0.217	Basal metabolic rate	ukb-b-16446
0.11	0.067	0.101	BMI	ukb-b-19953
0.08	0.110	0.468	Diastolic BP	ieu-b-39
0.13	0.111	0.240	Fat-free mass	ukb-b-13354
-0.12	0.054	0.027	HDL-C	ieu-b-109
-0.13	0.108	0.227	Impedance	ukb-b-19921
0.12	0.094	0.200	Waist circumference	ukb-b-9405
0.13	0.090	0.149	Water mass	ukb-b-14540

Supplementary References

1. Gallagher, C. S. *et al.* Genome-wide association and epidemiological analyses reveal common genetic origins between uterine leiomyomata and endometriosis. *Nat. Commun.* **10**, 1–11 (2019).
2. Bulik-Sullivan, B. *et al.* LD score regression distinguishes confounding from polygenicity in genome-wide association studies. *Nat. Genet.* **47**, 291–295 (2015).
3. Lonsdale, J. *et al.* The Genotype-Tissue Expression (GTEx) project. *Nat. Genet.* **45**, 580–5 (2013).
4. de Leeuw, C. A., Mooij, J. M., Heskes, T. & Posthuma, D. MAGMA: Generalized Gene-Set Analysis of GWAS Data. *PLoS Comput. Biol.* **11**, 1–19 (2015).
5. Watanabe, K., Taskesen, E., Van Bochoven, A. & Posthuma, D. Functional mapping and annotation of genetic associations with FUMA. *Nat. Commun.* **8**, 1–11 (2017).
6. Benner, C. *et al.* Prospects of Fine-Mapping Trait-Associated Genomic Regions by Using Summary Statistics from Genome-wide Association Studies. *Am. J. Hum. Genet.* **101**, 539–551 (2017).
7. Wang, G., Sarkar, A., Carbonetto, P. & Stephens, M. A simple new approach to variable selection in regression, with application to genetic fine mapping. *J. R. Stat. Soc. Ser. B Stat. Methodol.* **82**, 1273–1300 (2020).
8. Liberzon, A. *et al.* Molecular signatures database (MSigDB) 3.0. *Bioinformatics* **27**, 1739–1740 (2011).
9. Giambartolomei, C. *et al.* Bayesian Test for Colocalisation between Pairs of Genetic Association Studies Using Summary Statistics. *PLoS Genet.* **10**, (2014).
10. Zhu, Z. *et al.* Integration of summary data from GWAS and eQTL studies predicts complex trait gene targets. *Nat. Genet.* **48**, 481–487 (2016).
11. Cuellar-Partida, G. *et al.* Complex-Traits Genetics Virtual Lab: A community-driven web platform for post-GWAS analyses. *bioRxiv* 518027 (2019). doi:10.1101/518027
12. Hemani, G. *et al.* The MR-base platform supports systematic causal inference across the human phenome. *Elife* **7**, 1–29 (2018).
13. Ruth, K. S. *et al.* Using human genetics to understand the disease impacts of testosterone in men and women. *Nat. Med.* **26**, 252–258 (2020).
14. Verbanck, M., Chen, C. Y., Neale, B. & Do, R. Detection of widespread horizontal pleiotropy in causal relationships inferred from Mendelian randomization between complex traits and diseases. *Nat. Genet.* **50**, 693–698 (2018).
15. Qi, G. & Chatterjee, N. Mendelian randomization analysis using mixture models for robust and efficient estimation of causal effects. *Nat. Commun.* **10**, 1–10 (2019).
