# Supplementary material for: Evidence of a causal effect of genetic tendency to gain muscle mass on uterine leiomyomata
Source: Nat Commun. 2023 Feb 1;14:542. doi: 10.1038/s41467-023-35974-7 (PMC9892568; doi:10.1038/s41467-023-35974-7)
Supplement: Supplementary file 2 — Description of Additional Supplementary Files [file 41467_2023_35974_MOESM2_ESM.pdf]

Description of Additional Supplementary Files

Supplementary Data 1: Source Data File

Supplementary Data 2: Meta-analysis summary statistics from META-1 (limited to the top 10,000 variants from the previous UL meta-GWAS)

Supplementary Data 3: Meta-analysis summary statistics from META-2 (limited to the top 10,000 variants)

Supplementary Data 4: Results of colocalization of UL association signals and gene expression in GTEx v8 (cultured fibroblasts, skeletal muscle, uterus, and whole blood)

Supplementary Data 5: Results of SMR to test if gene expression mediates UL association (uterus)

Supplementary Data 6: Results of SMR to test if gene expression mediates UL association (whole blood)

Supplementary Data 7: Results of SMR to test if gene expression mediates UL association (skeletal muscle)

Supplementary Data 8: Results of SMR to test if gene expression mediates UL association (cultured fibroblasts)
