# Supplementary material for: Evidence of a causal effect of genetic tendency to gain muscle mass on uterine leiomyomata
Source: Nat Commun. 2023 Feb 1;14:542. doi: 10.1038/s41467-023-35974-7 (PMC9892568; doi:10.1038/s41467-023-35974-7)
Supplement: Supplementary file 11 — Reporting Summary [file 41467_2023_35974_MOESM11_ESM.pdf]

nature portfolio

Corresponding author(s): Eeva Sliz

Last updated by author(s): Oct 6, 2022

Reporting Summary

Nature Portfolio wishes to improve the reproducibility of the work that we publish. This form provides structure for consistency and transparency in reporting. For further information on Nature Portfolio policies, see our Editorial Policies and the Editorial Policy Checklist.

Statistics

For all statistical analyses, confirm that the following items are present in the figure legend, table legend, main text, or Methods section.

n/a	Confirmed	
☐	☒	The exact sample size (*n*) for each experimental group/condition, given as a discrete number and unit of measurement
☒	☐	A statement on whether measurements were taken from distinct samples or whether the same sample was measured repeatedly
☐	☒	The statistical test(s) used AND whether they are one- or two-sided<br>*Only common tests should be described solely by name; describe more complex techniques in the Methods section.*
☐	☒	A description of all covariates tested
☐	☒	A description of any assumptions or corrections, such as tests of normality and adjustment for multiple comparisons
☐	☒	A full description of the statistical parameters including central tendency (e.g. means) or other basic estimates (e.g. regression coefficient) AND variation (e.g. standard deviation) or associated estimates of uncertainty (e.g. confidence intervals)
☐	☒	For null hypothesis testing, the test statistic (e.g. *F*, *t*, *r*) with confidence intervals, effect sizes, degrees of freedom and *P* value noted<br>*Give P values as exact values whenever suitable.*
☐	☒	For Bayesian analysis, information on the choice of priors and Markov chain Monte Carlo settings
☒	☐	For hierarchical and complex designs, identification of the appropriate level for tests and full reporting of outcomes
☒	☐	Estimates of effect sizes (e.g. Cohen's *d*, Pearson's *r*), indicating how they were calculated

Our web collection on statistics for biologists contains articles on many of the points above.

Software and code

Policy information about availability of computer code

Data collection	In FinnGen, genotyping of the samples was performed using Illumina and Affymetrix arrays. Chip genotyped samples were pre-phased with Eagle 2.3.5. Genotype imputation was carried out by using the Finnish population-specific SISu v3 reference panel with Beagle 4.1.
Data analysis	In FinnGen, the GWAS analysis was completed using the Scalable and Accurate Implementation of 511 Generalized (SAIGE) software version 0.36.3.2. The analysis software to perform meta-analyses was METAL (V.2011-03-25). The SNP-based heritability, genetic correlations, and genomic inflation factor were estimated using LDSC regression implemented in LDSC software (v1.0.1). The SNP-based heritability was additionally estimated using SumHer (ldak5.2.linux). Conditional analyses were conducted using GCTA (1.93.0 beta Linux) and fine-mapping was performed with FinnGen finemapping pipeline using SuSiE (https://github.com/FINNGEN/finemapping-pipeline, accessed on 2/2/2022). The functional annotations of the GWAS results were completed using a web-based platform FUMA (accessed on 05/18/2022). Gene expression colocalization and mediation analyses were conducted using coloc R library (5.1.0.1) and summary statistics Mendelian randomization implemented in Complex Traits Genetics Virtual Lab (CTG-VL; beta-0.4), respectively. RegulomeDB (2.0.3) was used to identify regulatory elements overlapping with the GWAS signals. To test for causal inferences between UL and traits genetically correlated with UL, we performed bi-directional two-sample Mendelian randomization using TwoSampleMR (0.5.6), MR-PRESSO (1.0), and MRMix (0.1.0) R libraries.

For manuscripts utilizing custom algorithms or software that are central to the research but not yet described in published literature, software must be made available to editors and reviewers. We strongly encourage code deposition in a community repository (e.g. GitHub). See the Nature Portfolio guidelines for submitting code & software for further information.

Data

Policy information about availability of data

All manuscripts must include a data availability statement. This statement should provide the following information, where applicable:

- Accession codes, unique identifiers, or web links for publicly available datasets
- A description of any restrictions on data availability
- For clinical datasets or third party data, please ensure that the statement adheres to our policy

The individual-level data are available under restricted access for legal and ethical reasons; access can be obtained by application (please see https://www.finngen.fi/en/access_results for more details; access to FinnGen GWAS summary statistics can be applied by this online form; access to individual-level data and genotype data is managed by the Finnish Biobank Cooperative at the Fingenious portal).

The genome-wide association data generated in this study (META-2) have been deposited in the NHGRI-EBI GWAS Catalog database. The results of META-1 (UL associations limited to the top 10,000 variants from the previous study) are provided in the Supplementary Data. The summary-level data other than the genetic associations generated in this study are provided in the Supplementary Information.

The genome-wide data from the previous UL-GWAS by Gallagher et al. used in this study are available in the NHGRI-EBI GWAS Catalog database under accession code GCST009158. The genome-wide data of the 20 metabolic and anthropometric traits used in calculating genetic correlations and causal inferences are available at the MRC-IEU GWAS database (the trait-specific data can be extracted using the trait IDs listed in Table S16).

Human research participants

Policy information about studies involving human research participants and Sex and Gender in Research.

Reporting on sex and gender	Uterine leiomyomata are tumours of female genital tract and, thus, our analyses are limited to female sex only. This has been reported in multiple parts of the manuscript text.
Population characteristics	Cases were required to have an entry of ICD-10: D25, ICD-9: 218, or ICD-8: 21899, and participants who had no records of these entries were deemed as controls. The mean age at the diagnosis was 46.8 years. Additional phenotype information (e.g. BMI) is not available for most FinnGen participants.
Recruitment	We have used registry-based data from FinnGen: the FinnGen project utilizes genome information from a nationwide network of Finnish biobanks that are linked with digital health records from national hospital discharge (available from 1968), death (1969-), cancer (1953-), and medication reimbursement (1995-) registries.
Ethics oversight	The Coordinating Ethics Committee of the Hospital District of Helsinki and Uusimaa (Helsingin ja Uudenmaan Sairaanhoitopiiri, HUS) approved the FinnGen study protocol Nr HUS/990/2017. The FinnGen study is approved by Finnish Institute for Health and Welfare (Terveyden ja hyvinvoinnin laitos, THL), approval number THL/2031/6.02.00/2017, amendments THL/1101/5.05.00/2017, THL/341/6.02.00/2018, THL/2222/6.02.00/2018, THL/283/6.02.00/2019, THL/1721/5.05.00/2019, Digital and population data service agency VRK43431/2017-3, VRK/6909/2018-3, VRK/4415/2019-3 the Social Insurance Institution (Kansaneläkelaitos, KELA) KELA 58/522/2017, KELA 131/522/2018, KELA 70/522/2019, KELA 98/522/2019, and Statistics Finland TK-53-1041-17. The Biobank Access Decisions for FinnGen samples and data utilized in FinnGen Data Freeze 5 include: THL Biobank BB2017_55, BB2017_111, BB2018_19, BB_2018_34, BB_2018_67, BB2018_71, BB2019_7, BB2019_8, BB2019_26, Finnish Red Cross Blood Service Biobank 7.12.2017, Helsinki Biobank HUS/359/2017, Auria Biobank AB17-5154, Biobank Borealis of Northern Finland_2017_1013, Biobank of Eastern Finland 1186/2018, Finnish Clinical Biobank Tampere MH0004, Central Finland Biobank 1-2017, and Terveystalo Biobank STB 2018001.

Note that full information on the approval of the study protocol must also be provided in the manuscript.

Field-specific reporting

Please select the one below that is the best fit for your research. If you are not sure, read the appropriate sections before making your selection.

[x] Life sciences   [ ] Behavioural & social sciences   [ ] Ecological, evolutionary & environmental sciences

For a reference copy of the document with all sections, see nature.com/documents/nr-reporting-summary-flat.pdf

Life sciences study design

All studies must disclose on these points even when the disclosure is negative.

Sample size	We used the maximal sample size available for our study. The case figure was nearly doubled in our study compared with the previous UL GWAS and, thus, we had more power to detect novel loci.
Data exclusions	In FinnGen, sample quality control (QC) was performed to exclude individuals with high genotype missingness (>5%), ambiguous gender, excess heterozygosity (4SD) and non-Finnish ancestry. Regarding variant QC, all variants with low Hardy-Weinberg equilibrium (HWE) p-value

	(<1e-6), high missingness (>2%) and minor allele count (MAC)<3 were excluded. In post-imputation QC, variants with imputation info<0.6 were excluded.
Replication	We report meta-analyzed GWAS findings that were obtained using data from two sources, namely FinnGen and the previous UL meta-GWAS. We found that the results are consistent in the studied data sets with very little to no heterogeneity, indicative of a successful replication.
Randomization	In most part, not relevant to our study - this is a genetic association study conducted in a case-control setting and randomization is not applicable to this methodology.<br>Regarding Mendelian randomization analyses, the random distribution of genotypes at meiosis is considered comparable to exposure randomization in RTC according to the principles of the methodology.
Blinding	Not relevant to our study - this is a genetic association study conducted using registry-based health data and blinding is not applicable to this methodology.

Reporting for specific materials, systems and methods

We require information from authors about some types of materials, experimental systems and methods used in many studies. Here, indicate whether each material, system or method listed is relevant to your study. If you are not sure if a list item applies to your research, read the appropriate section before selecting a response.

Materials & experimental systems

n/a	Involved in the study	
☒	☐	Antibodies
☒	☐	Eukaryotic cell lines
☒	☐	Palaeontology and archaeology
☒	☐	Animals and other organisms
☒	☐	Clinical data
☒	☐	Dual use research of concern

Methods

n/a	Involved in the study	
☒	☐	ChIP-seq
☒	☐	Flow cytometry
☒	☐	MRI-based neuroimaging
